# Mapping human vulnerability to climate change in the Brazilian Amazon: The construction of a municipal vulnerability index

**DOI:** 10.1371/journal.pone.0190808

**Published:** 2018-02-14

**Authors:** Júlia Alves Menezes, Ulisses Confalonieri, Ana Paula Madureira, Isabela de Brito Duval, Rhavena Barbosa dos Santos, Carina Margonari

**Affiliations:** 1 Grupo de Estudos Transdisciplinares em Saúde e Ambiente, Instituto René Rachou, Fundação Oswaldo Cruz, Belo Horizonte, Minas Gerais, Brasil; 2 Departamento de Engenharia de Biossistemas, Universidade Federal de São João del-Rei, São João del-Rei, Minas Gerais, Brasil; 3 Grupo de Estudos em Leishmanioses, Instituto René Rachou, Fundação Oswaldo Cruz, Belo Horizonte, Minas Gerais, Brasil; Universidade de Brasilia, BRAZIL

## Abstract

Vulnerability, understood as the propensity to be adversely affected, has attained importance in the context of climate change by helping to understand what makes populations and territories predisposed to its impacts. Conditions of vulnerability may vary depending on the characteristics of each territory studied—social, environmental, infrastructural, public policies, among others. Thus, the present study aimed to evaluate what makes the municipalities of the state of Amazonas, Brazil, vulnerable to climate change in the context of the largest tropical forest in the world, and which regions of the State are the most susceptible. A Municipal Vulnerability Index was developed, which was used to associate current socio-environmental characteristics of municipalities with climate change scenarios in order to identify those that may be most affected by climate change. The results showed that poor adaptive capacity and poverty had the most influence on current vulnerability of the municipalities of Amazonas with the most vulnerable areas being the southern, northern, and eastern regions of the state. When current vulnerability was related to future climate change projections, the most vulnerable areas were the northern, northeastern, extreme southern, and southwestern regions. From a socio-environmental and climatic point of view, these regions should be a priority for public policy efforts to reduce their vulnerability and prepare them to cope with the adverse aspects of climate change.

## Introduction

The Intergovernmental Panel on Climate Change (IPCC) defines vulnerability as “the propensity or predisposition to be adversely affected” [1], which encompasses the basic components of exposure, sensitivity and adaptive capacity. These three components are capable of influencing vulnerability and may increase or decrease it according to characteristics inherent to the human or natural system of interest. In recent years, vulnerability analysis has followed a multifactorial approach that incorporates distinct social, political, economic and environmental conditions, including future climate change scenarios [2–4]. In this sense, vulnerability assessments have been effective tools for enabling, among other uses, vulnerability mapping [2,4–7]. Among the advantages that understanding the geography of vulnerability to climate change provides are enhanced disaster risk management; reduced exposure of human and ecological assets; and identification of particularly vulnerable populations [5].

Indicators have been commonly used as tools for assessing the vulnerability of populations and territories. They not only allow comparisons to be made among the evaluated systems, but also facilitate the visualization of information about what needs to be adapted in a simple and objective way [4,8–11]. This method has been applied in different ways by various authors, either on a regional or national scale [12–16]. Using a quantitative approach, Brooks et al. [17] and Moss et al. [18] established a series of key indicators for assessing the vulnerability and adaptive capacity of countries throughout the world. On a regional scale, Sullivan & Meigh [19] demonstrated the applicability of the Climate Vulnerability Index for identifying populations that are most susceptible to climate impacts; Yusuf & Francisco [7] combined climate risk assessments, human and ecological sensitivity, and adaptive capacity to produce maps of vulnerability to climate change for Southeast Asia; and Thornton et al [20] mapped vulnerability and human poverty in Africa by identifying key elements to included in their vulnerability indicator.

In Brazil, the indicator method has also been applied to assessing vulnerability to climate change. Studies have related the impacts of climate change to increased vulnerability to hydrometeorological events at the national level [21], the demographic and migration processes in various regions of the country [22–24], and to the socioeconomic conditions of Brazilian municipalities that create socio-climatic hotspots [25]. A methodology involving synthetic indicators was developed and refined by the Oswaldo Cruz Foundation since the 2000s, which began with the construction of an indicator of vulnerability of the Brazilian population to climate impacts on health [26]. Development of this methodology included the study of social, environmental and health vulnerabilities of the Northeast Region of Brazil [27], of the municipalities of the state of Rio de Janeiro [2] and, more recently, of the state of Minas Gerais [28].

The Amazon Region has been facing challenges inherent to the development projects taking place in the countries that comprise it, such as combining the need to promote social and economic progress with environmental conservation, which includes climatic issues. On the one hand, ecosystems of the Amazon are important strongholds of biodiversity and natural resources and play an active role in biosphere-atmosphere exchange on both regional and global scales, and thus serve an essential function in climatic balance [29–31]. On the other hand, the socioeconomic conditions of human populations in the Amazon are at a great disparity with national averages of their respective countries, which is even more severe when analyzed from the perspective of traditional populations, whose estimated contingent is 1.6 million people [32–34]. Overcoming this condition finds obstacles in the current anthropic pressures placed upon the Amazon due to the expansion of agriculture and “economic development”—both have profoundly reduced biodiversity, cultural assets and ecosystem services. The effects of the combination of these elements with global climate change are apparent in the region. In the case of the Brazilian Amazon, the last 10 years have experienced the most severe events of the century related to droughts (2005 and 2010) and floods (2009 and 2012) due to changes in rainfall in the Amazon Basin [29,35–39].

The state of Amazonas, Brazil, exhibits the same social and environmental patterns observed in other regions of the Amazon. The state is, for the most part, covered by forest and faces important issues related to: 1) land use policies associated with farming and the construction of roads and hydroelectric dams; 2) health, due to high incidence rates of infectious diseases and the need to adapt infrastructure to peculiar socio-spatial conditions—mobility is primarily fluvial; and 3) the social vulnerability of the traditional population prevalent in the State and dependent on extractivism and family farming [40–45]. These conditions give rise to a peculiar sociodemographic situation in the state, mainly related to the influence of its large rivers and extensive forests. The construction of urban environments amid the world’s largest tropical rainforest exhibits nuances that extrapolate demography and are conditioned by the flood regime of the main rivers, such as the Solimões and Amazonas [46,47]. This regime determines the relationship between the human population and the natural environment, and is vital to aspects of daily life, such as hunting, fishing, access to water and rural production [48,49]. However, climate changes have been observed in the region, such as longer dry seasons and increased river flow, along with projections of reduced precipitation and rise in temperature by the end of the century [29,50–53]. These aspects of the climate, coupled with the dependence of the population of Amazonas on ecosystem services, constitute a scenario of threat to human well-being, which can be aggravated by the poor social indicators of the region.

Understanding the weaknesses and capabilities of the municipalities of the state of Amazonas to respond to climate change, considering the Brazilian context, is the first step towards enhancing and preparing populations to cope with climate hazards. Therefore, aiming to support future adaptation policies for local populations, this study presents a method to map human vulnerability to climate change in the state of Amazonas, Brazil. To do so, the Municipal Vulnerability Index was constructed, which combines regionalized climate scenarios with socioeconomic, demographic, environmental and health conditions of populations in order to better understand and compare vulnerability profiles of the 62 municipalities of the state of Amazonas. These results are expected to provide a better understanding of the weaknesses and potentialities of municipalities to deal with climate impacts, given that some characteristics, such as climatic conditions and health infrastructure, are distributed in a very heterogeneous way even among municipalities of the same state.

### Conceptual framework

#### Vulnerability to climate change

The conceptual framework is an essential part of a vulnerability analysis since it defines the fundamental assumptions and the relationships among their components. The focus of this study was to assess the vulnerability of populations of specific territories, the municipalities. Thus, it is understood that in each site a variety of biophysical and socioeconomic factors shape both the risks and vulnerabilities to climate change. The concept that best fits the approach, and used in the present work, is that of contextual vulnerability, defined by the fifth IPCC report [54] as “a present inability to cope with external pressures or changes, such as changing climate conditions, and a characteristic of social and ecological systems generated by multiple factors and processes”.

Multiple processes emerge from the varied socio-environmental and economic developmental conditions, as well as particular climate threats of each locality. Thus, this study encompassed these processes and factors in three basic components of vulnerability–exposure, sensitivity and adaptive capacity. Exposure concerns the nature and intensity of environmental and socio-political stress experienced by a system, encompassing both long-term changes in climatic conditions and the variability represented by extreme events [4,55]. In this work, exposure was considered in two temporal spaces, the present and future. Present exposure was represented by biophysical conditions related to the environment and hydrometeorological disasters. Future exposure was represented by climatic projections of the regional model ETA-HADGEM-ES (long-term changes). According to McCarthy [56], sensitivity is the degree to which a system can be affected by climate change, whether directly or indirectly, and is related to the intrinsic characteristics of the impacted system. The present study worked with the indirect effects of climate resulting from the possible damage that can occur, more specifically on specific population groups, due to either their social or financial condition. Adaptive capacity is related to the ability of populations to adjust to possible damage and respond to the consequence of climate impacts [1], and which focuses on the conditions of community organization, such as the role of institutions, governance and management.

**Fig 1:**
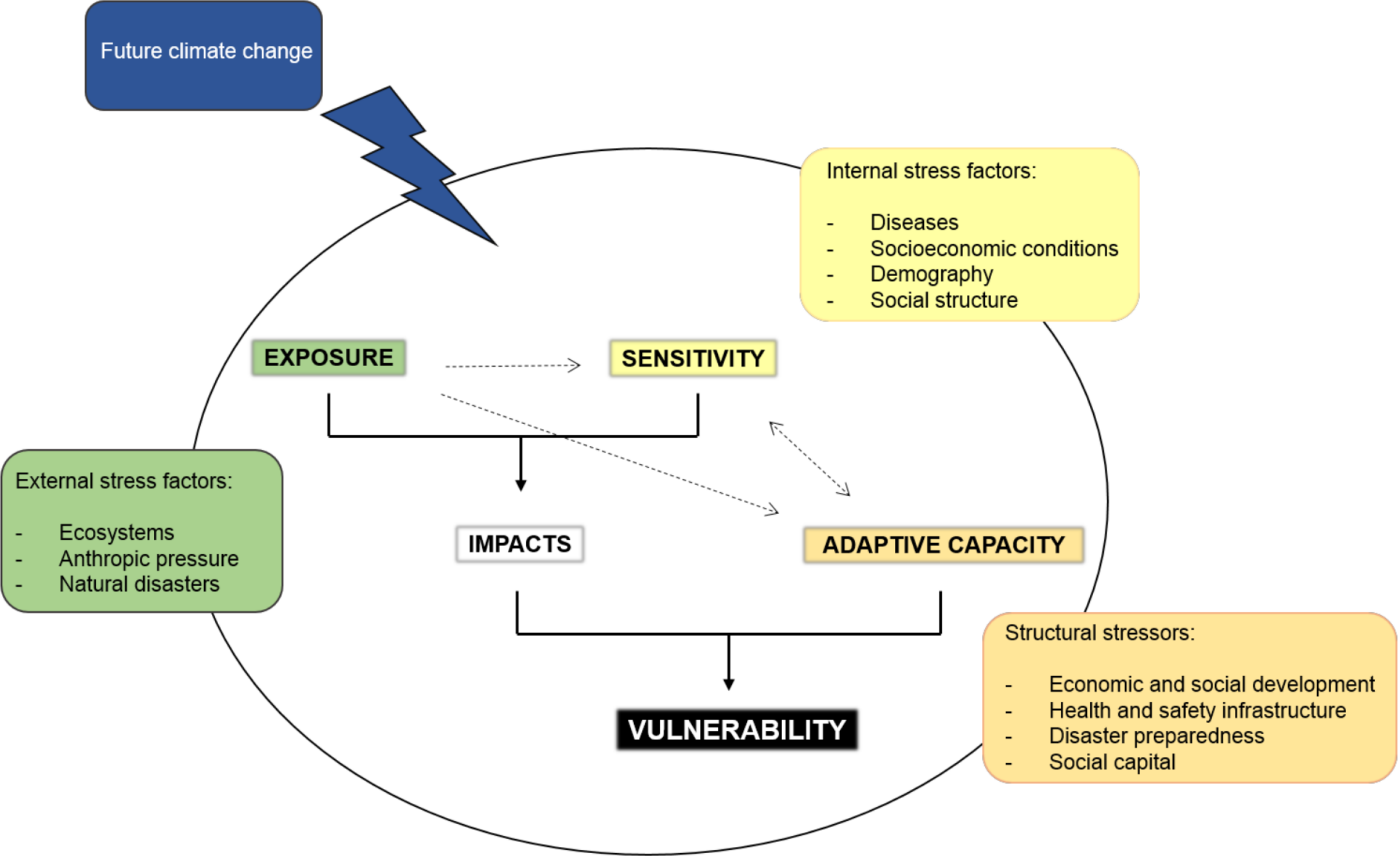
Conceptual framework showing the relationships among vulnerability components. The circle represents a municipality in which the conditions of exposure, sensitivity and adaptive capacity determine the vulnerability profile of the population. The boxes exemplify some of the conditions considered critical for each vulnerability component; the green box is related to exposure, the yellow box to sensitivity and the orange box to adaptive capacity. Climate risk is represented by future climate change. (Adapted from Allen Consulting Group, 2005).

It was assumed that the human populations at each location are subjected to multiple and specific tensions that combine with each other to determine climate impacts on a regional scale, with feedback between human and natural systems. Considering that the division between these systems is only arbitrary, since anthropic actions and social structure are not dissociated from the natural environment, the present study considered the “municipality” as the unit of analysis since it represents an integrated system in which impacts will occur [55]. In this sense, the present work has developed a conceptual framework for the municipalities of the state of Amazonas, which considers systems that possess some peculiar characteristics that shape their vulnerability profile and that may be exacerbated by future climate change (Fig 1).

In Fig 1, the circle represents a system, such as a municipality, in which various physical, natural, economic, social, political and structural conditions shape the vulnerability profile of a municipality. This profile is, somehow, determined by exposure and sensitivity, since these components contribute to determining the magnitude of the potential impacts of future climate change. The magnitude of impacts may depend on the capacity of a population and of its municipality to cope with the adverse effects of climate. The adaptive capacity is strictly linked to the sensitivity of the system, since this component includes certain characteristics intrinsic to a population that determine its ability to recover from impacts. Thus, the vulnerability profile constructed may be altered by the influence of climate change, here represented by gradual alterations in temperature and precipitation over time (from the present to the period of 2041–2070).

#### Concepts and variables of the methodological scheme

The present work comprises the unfolding of a national-scale project whose objective was to construct indicators of vulnerability for some Brazilian municipalities, in the context of climate change, in order to support future adaptive actions. Financed by public resources (Ministry of Environment), a Municipal Vulnerability Indicator was built for six Brazilian states and their municipalities, including the state of Amazonas. The methodology was designed to be easily-executed by local managers, and so that the constructed indices could be promptly interpreted and updated, thus allowing the monitoring of the vulnerability profile of the municipalities of Amazonas over time. In the end, the focus of the study was to estimate how climate change may influence the vulnerability of the state of Amazonas considering its current socio-environmental conditions.

The variables chosen to compose the methodological scheme possessed the following criteria: 1) should be mainly of free access; and 2) should be systematically updated. Most of these variables were selected based on what has been established by other studies developed for Brazil, whereas other variables were specific to this study [2,27,57,58]. All variables passed through validation by a panel of experts comprising managers, decision makers, non-governmental organizations and researchers from various parts of the country who had expertise in the thematic areas studied–disasters, social indicators, adaptation, climatology and health, among others. After this first evaluation, successive meetings were held in the state of Amazonas, as well as in the other states that were part of the national project, to discuss the reliability and adequacy of the indicators relative to local reality.

According to Adger’s definition for exposure [55] (the nature and degree to which a system experiences environmental or socio-political stress systems), two factors were considered in the present evaluation. The first, essentially environmental, considers the relevance of the Amazonian ecosystem to the resident population (ecosystem services) and to the regional dynamics of the terrestrial-atmosphere energy flux, using as proxy the extension of vegetation cover and the area of deforestation in recent years. The assumption here is that the maintenance of the natural environment makes the municipality less exposed to the effects of climate change due to the conservation of associated ecosystem services (e.g. temperature control, air quality, clean water, among others). Stress or destruction of ecosystems can increase the physical vulnerability of human settlements as well as reduce the possibilities for traditional populations (e.g. riverine) to adequately exploit livelihoods, including alternative food sources. The second important factor evaluated was exposure to risk of hydrometeorological disasters, a phenomenon provided by both social and environmental conditions and related to climate variability. This exposure was assessed through the susceptibility to disasters and also by historical records of occurrence of such events. Exposure indicators were positively related to vulnerability.

For sensitivity (the degree to which a system is affected, either adversely or beneficially, by climate-related stimuli [56]), some conditions intrinsic to the municipalities that may make them more susceptible to climate impacts were considered. The focus was on the susceptibility of the population, hence the variables to be chosen to compose the sensitivity index should reflect inequities in the process of socioeconomic development, since this engenders conditions of social marginalization and generates some social groups that are more vulnerable to climate and its impacts than others. The factors considered were those related to climate-sensitive diseases, which may be affected by expected changes in temperature and precipitation; those related to human poverty, in their monetary and non-monetary dimensions; and, finally, those related to the sociodemographic characteristics of the population. All of the indicators selected to comprise sensitivity have been associated with increased vulnerability and have been related with the indirect effects of climate resulting from possible damage that may occur to specific groups of populations (e.g elderly, people with disabilities, women lacking education).

For adaptive capacity (the ability of systems, institutions, people, and other organisms to adjust to potential damage, to take advantage of opportunities or to respond to consequences [59]), the main focus was governance and social capital. Governance and local institutions are considered essential to reducing vulnerability and enhancing adaptation at the local level, since they help to shape the risks associated with climate impacts and to define the degree to which individuals will respond to the impact, including the collective scope [60]. This aspect was evaluated by 1) the municipal competence to promote economic development in key areas such as health, education and employment, with regards to quality and access, and whose performance has been measured since 2008 by the FIRJAN Municipal Development Index; and 2) the presence of public institutions of security, risk management and health that could provide support and actions to reduce harm in the face of the expected negative impacts of climate change. As for social capital, its ability to increase the resilience of populations is well known, either through the proximity of different groups, closer ties between individuals who share a social identity or through networks of trust that are established between the different levels of authority [61–63]. For this, a proxy measure for the possibility of citizen participation in the political process was used, namely the representation on councils or consortia. The variables used here are inversely related to vulnerability, since municipalities with better levels of governance and socio-institutional arrangements are better able to deal with climate impacts, thus reducing vulnerability.

A future climate component was associated with the other basic elements of vulnerability to represent the extent to which the municipalities could be impacted by the predicted climate change in the state of Amazonas. Thus, the present study evaluated possible future climate change (2041–2070) in relation to the present (1961–1990), as projected by the model ETA-HadGem-ES considering climatic parameters related to temperature and precipitation. These data were municipalized for the entire state of Amazonas (see “Construction of the Climate Scenario” section) to serve as a predictor of the occurrence of climatic extremes in the near future. The assumption was that areas with the greatest predicted changes in temperature and precipitation would be those most exposed to climatic hazards. Table 1 describes the indicators used and their relation to vulnerability. The raw data for each indicator is available in the supplementary S3–S6 Tables.

**Table 1:** Indices that compose the vulnerability, their calculation, short description, and their relationship to the vulnerability.

MVI COMPONENT	INDEX AND CALCULATION	DESCRIPTION OF THE INDICATORS	RELATIONSHIP TO THE VULNERABILITY
**EXPOSURE INDEX**	**• Vegetation Cover Index (VCI)** • *VCI* = (*Score of vegetation cover* + *Score of deforestation*) / 2	**Native vegetation cover:** percentage of the area of the municipality covered by native vegetation in 2014 divided by total municipal land area. Proxy of ecosystem services.	The higher the vegetation cover, the less exposed/vulnerable.
		**Accumulated deforestation:** percentage of the original vegetation area deforested between 2000 and 2013 in relation to the original vegetation cover area in 2000. Indicates anthropic pressure upon the forest.	The higher the accumulated deforestation for the time series, the more exposed/vulnerable.
	**• Index of Natural Disasters (IND)** • *IDN* = (*NDSI* + *NDOI*) / 2 **• Natural Disasters Susceptibility Index (NDSI)** • *NDSI* = (*Score of population at risk* + *Score of CDD*) / 2 **• Natural Disasters Occurrence Index (NDOI)** • *NDOI* = (*Score of disaster occurrence* + *Score of deaths related to disasters*) / 2	**Population at risk:** the percentage of the total population living in areas of high and very high risk for landslides and hydrological events in relation to the total population. Suggestive of human losses that may result from natural disasters.	The higher the percentage of people at risk, the more exposed/vulnerable.
		**Consecutive dry days (CDD):** climate parameter that indicates greater propensity for dry periods.	The higher the average CDD, the more exposed / vulnerable.
		**Disaster occurrence between 1991–2012:** percentage of events that occurred in the municipality in relation to all events that occurred in the state. Used as an indication of the disasters’ burden on each municipality.	The higher the proportion of events in relation to the state, the greater the sensitivity/vulnerability.
		**Deaths related to natural disasters:** the proportion of deaths reported in the municipality in relation to the events in the municipality. Used as an indication of the fatality of recorded disasters.	The higher proportion of deaths, the higher sensitivity/vulnerability.
**SENSITIVITY INDEX**	**• Diseases Associated with Climate Index (DACI):** Dengue; Malaria; American Cutaneous Leishmaniasis; Accidents with poisonous animals (spider, snake, and scorpion) • *DACI* = (*Score of the proportion of cases* + *Score of the incidence of the disease* + *Score of the tendency of the incidence rate*) / 3	**Proportion of cases of the disease:** number of cases in the municipality in relation to the total number of cases in the state, in the time series.	The higher the proportion of cases in the city, the more sensitive/vulnerable.
		**Incidence rate:** the average incidence rate per 100,000 in the time series was used to measure the risk of the disease during the time series. For Malaria, the Annual Parasite Index was used, which considers the incidence per 1,000 inhabitants.	Higher average incidence rates, higher sensitivity/vulnerability.
		**Tendency of the disease’s incidence rate:** the incidence rate was used to determine how cases of the disease behaved (increase, decrease, stability) in the time series.	If the incidence showed a declining trend, the city was considered less sensitive; if the trend was an increase, the city was considered more sensitive.
	**• Poverty Index (PoI)** • *PoI* = (*Score of the probability of dying before age* 40 + *Score of illiterate population* + *Score of households whith inadequte sanitation* + *Score of the probability of dying before age* 5 + *Score of income bellow the poverty line*) / 5	**Probability of dying before age 40:** reflects the living conditions and mortality pattern of the population.	The higher the likelihood of dying before the age 40, the greater the sensitivity/vulnerability.
		**Percentage of the population aged over 25 that is illiterate:** ratio between the population aged 25 years or older who can not read or write and the total number of people in the same age group. The illiteracy rate was considered a proxy of education levels and population skills.	The higher the illiteracy rate, the higher the sensitivity.
		**Percentage of households with inadequate sanitation:** percentage of households with sanitation considered unsuitable including drinking water, sewage and garbage collection. Considered a basic need, it is related to the health conditions of the population.	Highest percentage of households without sanitation, increased vulnerability.
		**Probability of dying before the age of 5 per 1,000 live births:** reflects the living and health standards of the population.	More likely to die by the age of 5, higher the sensitivity/vulnerability.
		**Percentage of households with per capita income below the poverty line:** percentage of total households whose nominal per capita monthly income was up to ½ minimum wage. Monetary measure of poverty.	The higher the percentage of households with up to ½ minimum wage income, the higher the sensitivity/vulnerability.
	**• Sociodemographic Sensitivity Index (SSI)** • *SSI* = (*Current SSI* + *Future SSI*) / 2 **• Current SSI:** **• *C****urrent SSI* = (*Score of female householder* + *Score of young householder* + *Score of the infant population* + *Score of the elderly* + *Score of the population with disabilities* + *Score of the riverine population*) / 6 **• Future SSI:** • *Future SSI* = (*Score of the infant population* + *Score of the elderly population*) / 2	**Percentage of female householders with incomplete primary school or no education:** percentage of total households in the total population. Indicative of inequities related to gender and economics.	The higher the proportion of households headed by women, the greater the vulnerability.
		**Percentage of young householders:** percentage of total households in the total population whose head is between 10 and 29 years old	The higher the proportion of households headed by young individuals, the greater the vulnerability.
		**Percentage of the population under 5 years old:** percentage of the total population. Social group most vulnerable in disaster situations and who require permanent care.	The higher the percentage of infants in the population, the more sensitive/vulnerable.
		**Percentage of the population that is elderly (60 years old or older):** percentage of the total population. Social group most vulnerable in disaster situations and who require permanent care.	The higher the percentage of elderly in the population, the more sensitive/vulnerable.
		**Percentage of the population with disabilities:** percentage of the total population with some form of disability (visual, hearing, motor or intellectual). Most vulnerable social group in disaster situation and who may require specialized care.	The higher the percentage of the population with some form of disability, the more sensitive/vulnerable.
		**Riverine population:** percentage of the total population. Social group very dependent on natural resources for subsistence.	The higher the percentage of the population considered riverine, the more sensitive/vulnerable.
		**Projection of children aged 0 to 4 years old for the year 2040:** percentage of infant population in 2040.	The higher the percentage of infants expected in the population for 2040, the more sensitive/vulnerable.
		**Projection of the elderly aged 60 years old or older for the year 2040:** percentage of the elderly population in 2040.	The higher the percentage of elderly expected in the population for 2040, the more sensitive/vulnerable.
**ADAPTIVE CAPACITY INDEX**	**• Socioeconomic Structures Index (SEI):** This indicator is based on the FIRJAN Index of Municipal Development (FMDI), calculated for all Brazilian municipalities*. It follows the achievements and socioeconomic challenges of Brazil through the prism of municipal competence on a regular basis since 2008, and makes it possible to determine, with precision, whether the relative improvement experienced by a given municipality is the result of the adoption of specific policies. The FMDI varies between 0 and 1, where 0 represents the least developed and 1 the most developed municipality. This reasoning is opposite to that applied to vulnerability logic. Thus, for the construction of the Socioeconomic Structures Index (SEI), the reverse of FMDI was used, since the attributed scores followed the reasoning of vulnerability—if the city had high FMDI, it was considered more adapted and less vulnerable.	It reflects governance and encompasses the following: 1) structures for employment and income generation; 2) quality of education; and 3) quality of health care services (primary care).	The higher the score (closer to 1), the more sensitive/vulnerable.
	**• Institutions, Services, and Infrastructure for Adaptation Index (AdapI)** • *AdapI* = (*Score of secutiry institutions* + *Score of risk management instruments* + *Score of health care services*) / 3	**Existence of security institutions:** municipal civil defense, fire department and municipal guard. It reflects the structural capacity of the municipality to cope with climate impacts.	The greater the number of security institutions, the less vulnerable.
		**Existence of risk management instruments for landslides and floods:** mapping of risk areas, housing program, supervision control of risk areas, contingency planning, engineering projects, warning systems and risk register. It reflects the structural capacity of the municipality to cope with climate impacts and mitigate future impacts.	The larger the number of management instruments, the less vulnerable.
		**Health care** services (indicative of structural capacity of the municipality to provide basic services to the population): the information used were: 1) the number of hospital beds per 1,000 inhabitants; and 2) primary care coverage.	The higher the number of hospital beds and the coverage of basic care, the less vulnerable.
	**• Sociopolitical Organization Index (SOI)**	**Existence of municipal councils and consortia related to adaptation to climate (proxy of the community social capital):** environment, welfare and social development, basic sanitation, employment and labor, housing, transportation, urban development, and human rights. It reflects the social capital of the community.	The greater the number of councils and consortia, the less vulnerable.
**CLIMATE SCENARIO INDEX**	• *Temperature Index* = (*TMAXmean* + *TMINmean*) / 2 • *Precipitation Index* = (*Rx*5*day* + *R*95*p* + *CDD* + *PRCPTOT*) / 4	**Temperature anomalies:** average annual maximum temperature (TMAXmean) and average annual minimum temperature (TMINmean).	The higher the anomaly, the more vulnerable.
		**Precipitation anomalies:** Rx5day (monthly maximum consecutive 5-day precipitation); R95p (annual total precipitation when daily precipitation amount on day was higher than 95 percentile); CDD (maximum number of consecutive days with daily precipitation amount lower than 1mm); PRCPTOT (annual total precipitation on wet days).	The higher the anomaly, the more vulnerable.

* More information about the method and variables used to calculate the FIRJAN Index of Municipal Development can be obtained, in Portuguese, from the institution’s webpage (http://www.firjan.com.br/ifdm/).

## Material and methods

### Study area

**Fig 2:**
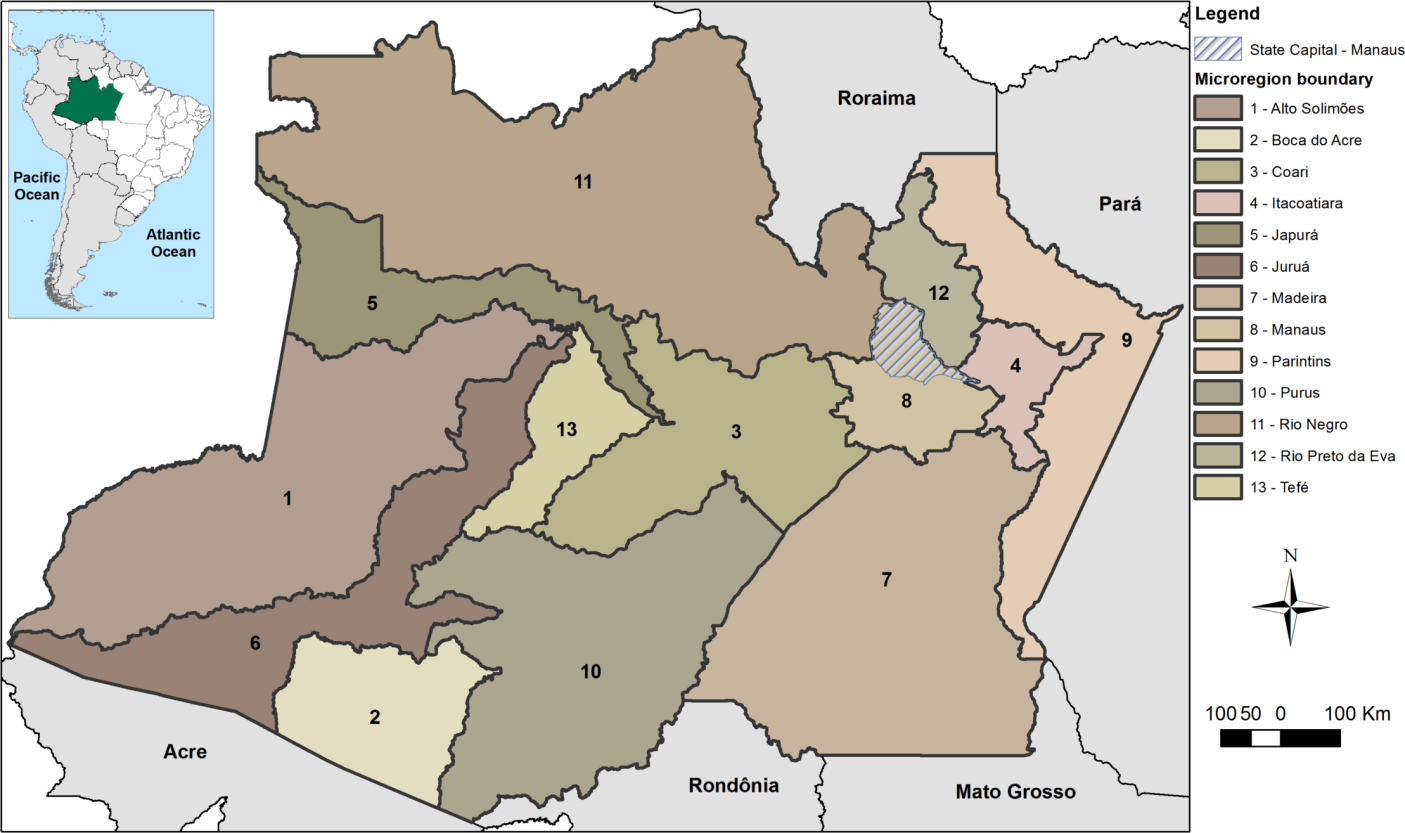
Microregions of the state of Amazonas, Brazil, and the location of the state capital, Manaus.

The state of Amazonas is located in the Northern Region of Brazil and is the largest state of the country, occupying about 18% of its territory (Fig 2). Although it has just 62 municipalities, the state capital of Manaus has a rich history, especially as a central harbor of the region, and contains 52% of the state’s population. For that reason, Manaus is considered the most populous city of the Brazilian Amazon. The state has 4,001,667 inhabitants, of which most are urban residents, although there is a considerable rural contingent of traditional groups: indigenous peoples who mainly live in legal reserves, and riverine peoples who reside along lakes and rivers [64]. Most of the population is male (50.3%), pardo (68.8%), receives at most half of the minimum wage (56.1%) and lives in houses lacking appropriate sanitation [65]. Environmentally, the state of Amazonas is considered only slightly deforested since 23.5% of its natural ecosystems are protected within conservation units and about 98% of the territory remains preserved. However, some regions, mostly in the southern portion, are historically linked to deforestation [66,67]. As part of the world’s largest hydrographic basin, the state of Amazonas is essentially fluvial. The mobility of goods and people occurs mainly via rivers; since access by land is not common, an estimated 20,000 km of waterways connect distant communities of the region [68]. To better contextualize the state of Amazonas, a brief description is provided that encompasses some environmental, economic and infrastructural conditions of the 13 microregions of the state (S1 Appendix).

### Construction of the climate scenario

Climatic data were made available by the INPE (National Institute of Space Research) and corresponded to the outputs of the climate model Eta-HadGem2-ES, which produces simulations of the current (1961–1990) and the future climate (2011–2040, 2041–2070, 2071–2100) considering the Representative Concentration Pathways (RCP) from the IPCC’s Fifth Report. The model Eta-HadGem2-ES corresponds to the regionalized model (Eta) nested within the global model HadGem-ES and presents a horizontal regular grid of 20km throughout South America. The regionalized model Eta exhibited strong agreement with the observational data of precipitation for South America and thus can be used for studies on climate change in Brazil [69].

Regarding the emission scenarios, Representative Concentration Pathways coming from the last IPCC assessment report—AR5 were used (IPCC 2013). The RCP refers to the number of radiative forcing measured in watts per square meter per year by 2100. The scenarios generated by IPCC are named according to their radiative forcing as follows: RPC2.6 (2.6 Wm-2), RCP4.5 (4.5 Wm-2), RCP6.0 (6.0 Wm-2) and RCP8.5 (8.5 Wm-2). The RCP8.5 combines, among other factors, high population growth, high energy demand, and high GHG emissions in the absence of climate change policies, corresponding to the RCP with the highest greenhouse gas emissions. This scenario includes radiative forcing beyond 8.5 W/m^2^ and CO_2_ concentrations of 1,370 ppm up to the year 2100. Considering the RCP8.5 as a pessimistic scenario and that shorter time periods are more appropriate for proposing and articulating public policies, the outputs of the Eta-HadGem-ES model considering the RCP8.5 were used for the period of 2041–2070.

Extreme climate indicators were constructed considering the absolute difference between the climatic parameters generated by Eta-HadGem-ES for the future climate slice (2041–2070) and the current slice (1961–1990). These indicators were generated with the assistance of the FClimDex program, and were the following: total annual rainfall for rainy days (i.e., days when rainfall is greater than or equal to 1 mm; PRCPTOT); total annual rainfall on days when rainfall is greater than that of the 95th percentile of the rainy days (R95p); monthly maximum consecutive 5-day precipitation (Rx5day); maximum number of consecutive dry days for the year, (i.e., days on which rainfall is less than 1 mm; CDD) and annual mean maximum (TMAXmean) and minimum (TMINmean) temperatures, in degrees Celsius.

These digital spatialized data bases were processed to generate maps of climatic anomalies for all the municipalities of the state of Amazonas. The spatial analysis and municipalization of climatic indicators were obtained using geostatistical modeling performed with the Geostatistical Analyst tool of ArcGIS Desktop software, version 10.2.2, and Surfer, version 11 [70]. The data were then interpolated to estimate the value of the climatic variables for the locations not available in the database.

### Source of data

Socioeconomic, environmental, infrastructural and climatic projections data for the 62 municipalities of the state of Amazonas were collected. Information about populations and infrastructure were obtained from the Brazilian Institute of Geography and Statistics (IBGE), FIRJAN Index of Municipal Development and the Department of Informatics of the Brazilian Health System (DataSus). The environmental data were collected from the PRODES project, linked to the INPE. The information related to natural disasters was obtained from the Brazilian Atlas of Natural Disasters, the National Water Agency (ANA) and the Mineral Resources Research Company (CPRM). Finally, for the climatic parameters related to precipitation and temperature to be used in the construction of the climate scenario, data were obtained from INPE.

### Scale of analysis

The scale of municipality was chosen because this is the smallest political-administrative unit in Brazil. Likewise, most of the data used in the study is available only on this scale of analysis. Focusing on municipality as a unit helps to identify local weaknesses and potentialities, as well as to compare vulnerabilities observed in each component–exposure, sensitivity, adaptive capacity and climate. For decision makers or state managers this is of particular relevance because these are the spheres from which financial resources are distributed to municipalities. Vulnerability mapping, therefore, allows the state to understand by which aspects the regions and their municipalities are most vulnerable, and thus allocate the resources as needed.

### Calculation of the Municipal Vulnerability Index

A synthetic indices approach was used to measure vulnerability, which is based on the selection of some indices from a potential set of variables, that are then systematically combined to generate levels of vulnerability [14]. This approach was chosen over evaluation modeling because 1) the results achieved are quite similar, and 2) for many developing countries, such as Brazil, the technical requirements, the availability of data and the financial resources needed to perform evaluation modeling are scarce or nonexistent [71]. For calculation of vulnerability, all the indicators were assumed to have equal importance, thus they have not received a priori weights [12]. The scale of analysis was that of the municipality.

**Fig 3:**
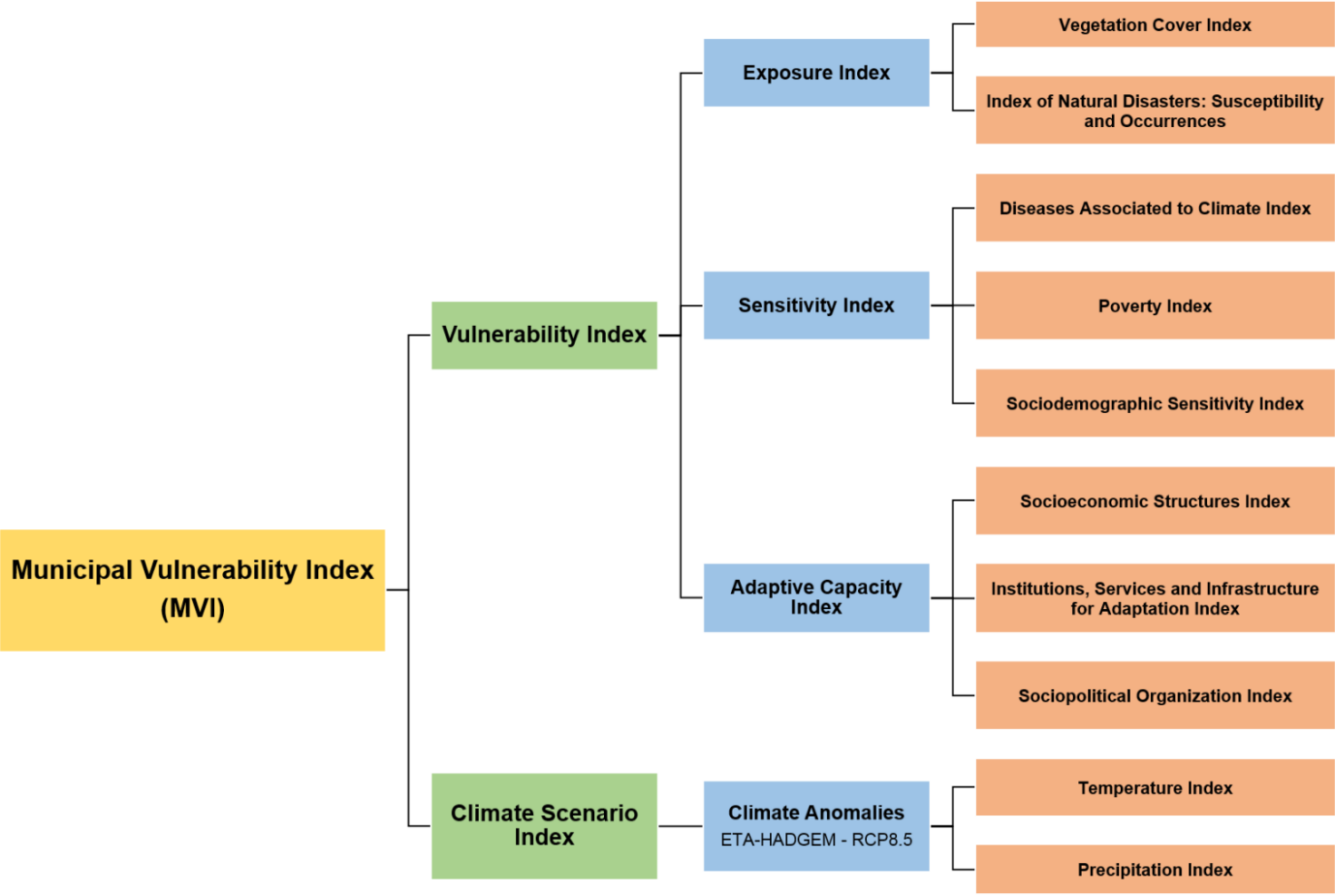
Methodological scheme. Organization of the indices chosen to generate the Municipal Vulnerability Index considering a pessimistic emission scenario (RCP8.5).

The present study constructed a Municipal Vulnerability Index (MVI) for the municipalities of the state of Amazonas, Brazil, from the successive aggregations of indices and sub-indices related to exposure, sensitivity, adaptive capacity and climate conditions of the territories. MVI represents, synthetically, the relationship between the current vulnerability, represented by the Vulnerability Index (VInd), and the future climate change, represented by the Climate Scenario Index (CSI), in a gradient between 0 and 1. Thus, 0 represents the lowest and 1 the highest vulnerability observed among the evaluated municipalities for all the indices. Fig 3 shows how the indices used to compose the MVI are related.

**Fig 4:**
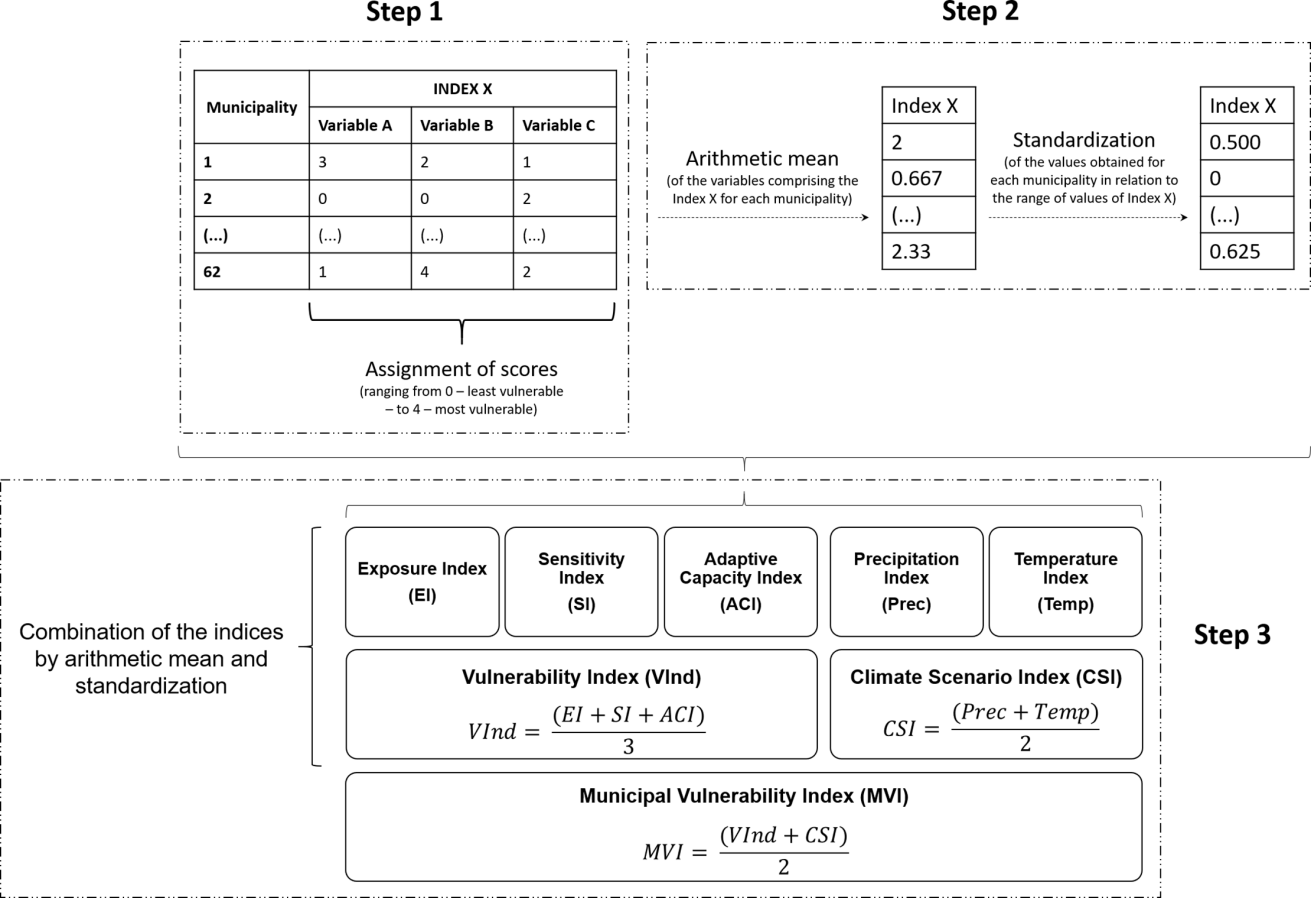
Diagram illustrating the steps of calculating all indicators and indices. Steps 1 and 2 comprise transforming the raw variables into indicators. Step 1 is representing the assignment of scores and Step 2 is illustrating the procedures of arithmetic mean and standardization to generate the indicators ranging from 0 (least vulnerable) to 1 (most vulnerable). Step 3 illustrates the aggregation of the indices to generate the final index, the Municipal Vulnerability Index.

Calculation of MVI involved three distinct steps: 1) attribution of gradual scores to the variables presented in Table 1; 2) aggregation of the scores using arithmetic mean and subsequent standardization to form the indices shown in the orange boxes of Fig 3 and in Table 1; and 3) combination of the indices using the same procedures used for arithmetic mean and standardization. Fig 4 shows the steps of calculating all indicators and indices used in the present work, as well as the successive aggregation of these indices to generate the MVI.

The first step was to attribute gradual scores, which represent vulnerability to the indicators shown in Table 1. Municipal variables were divided into groups for assigning scores so that the municipalities could receive a range of vulnerability scores. Each variable was analyzed individually and divided into percentiles—p5, p25, p75, p95 were used in most cases, but specificities as to the percentiles used can be seen in the S7 Table. For each interval a score ranging from 0 to 4 was assigned, where 0 was attributed to the least vulnerable group and 4 was attributed to most vulnerable group of municipalities, according to the rationale explained in Table 1. The division into classes for assignment of scores is done to allow the aggregation of variables that are in different units of measure. After the assignment of scores, all variables possess the same scale unit. For qualitative variables (present in some adaptive capacity indicators) values were arbitrarily applied as shown in S8 Table. Scoring was done considering the values for each variable in all 62 municipalities.

In vulnerability studies, exposure and sensitivity are considered to have the potential to increase the vulnerability of a system, while adaptive capacity the potential to decrease it. This logic was adopted in the present study. However, in view of the need to make information comprehensible to managers and decision makers, it was decided that the assignment of scores to variables would follow the same rationale for all indicators of exposure, sensitivity and adaptive capacity. In such a manner, having to interpret the indices of adaptive capacity in an inverse way was avoided, thereby simplifying the understanding of this component by decision makers and managers. Thus, for all indicators, scores were assigned considering whether the assessed characteristic increased or decreased vulnerability of the population. Therefore, in the adaptive capacity indices shown in S1 and S2 Tables, the highest values represented the most vulnerable (least adapted) municipalities, while the lowest values represented the least vulnerable (most adapted) municipalities.

The assignment of scores allows the aggregation of the variables that comprise each index through the arithmetic mean, which is the second step. These indices can be observed in the column “calculation of the indices” shown in Table 1 and in the orange boxes of the methodological scheme (Fig 3). However, the final value of the arithmetic mean does not vary between 0 and 1, as defined to represent the indices, so values of arithmetic means are standardized such that they range from 0 to 1 (Fig 4). Standardization is done in relation to the values of the other municipalities, using the following formula,

Indexp=(Index(obs)−MinimumIndex)/(MaximumIndex−MinimumIndex)

where:

Index p = standardized index;
Index obs = calculated index (for each municipality);
Minimum index = minimum value of the index among all municipalities;
Maximum index = maximum value of index among all the municipalities.

The same arithmetic mean and standardization procedures were used to calculate and aggregate all the indices of the methodological scheme in the final step. Accordingly, the MVI was constructed by the combination of its main indices–Vind and CSI. Likewise, VInd was formed by the combination of its three components—exposure, sensitivity and adaptive capacity—and the CSI was constructed by the aggregation of its climatic indices of temperature and precipitation anomalies (Fig 4). Thus, MVI represents, specifically, the current vulnerability to future climate change across the municipalities of the state of Amazonas.

This measure has the primary role of allowing the comparison among municipalities over time and space, since it allows both the observation of changes in current vulnerability according to future climate change, as well as the disaggregation of the main indices to explore which conditions most influenced their vulnerability. The method also enables the ranking of the municipalities according to their observed vulnerabilities, thereby providing a consistent and transparent methodology for the comparative assessment of the vulnerability of human populations to regional environmental changes. At the end, the indices developed (MVI, VInd and CSI) resulted in a relative measure of municipal vulnerability ranging between 0 and 1. For all indices, the intervals between 0 and 1 corresponded to: 0 to 0.2 (low); 0.201 to 0.400 (medium-low); 0.401 to 0.6 (medium); 0.601 to 0.8 (medium-high) and 0.801 to 1 (high).

## Results

**Fig 5:**
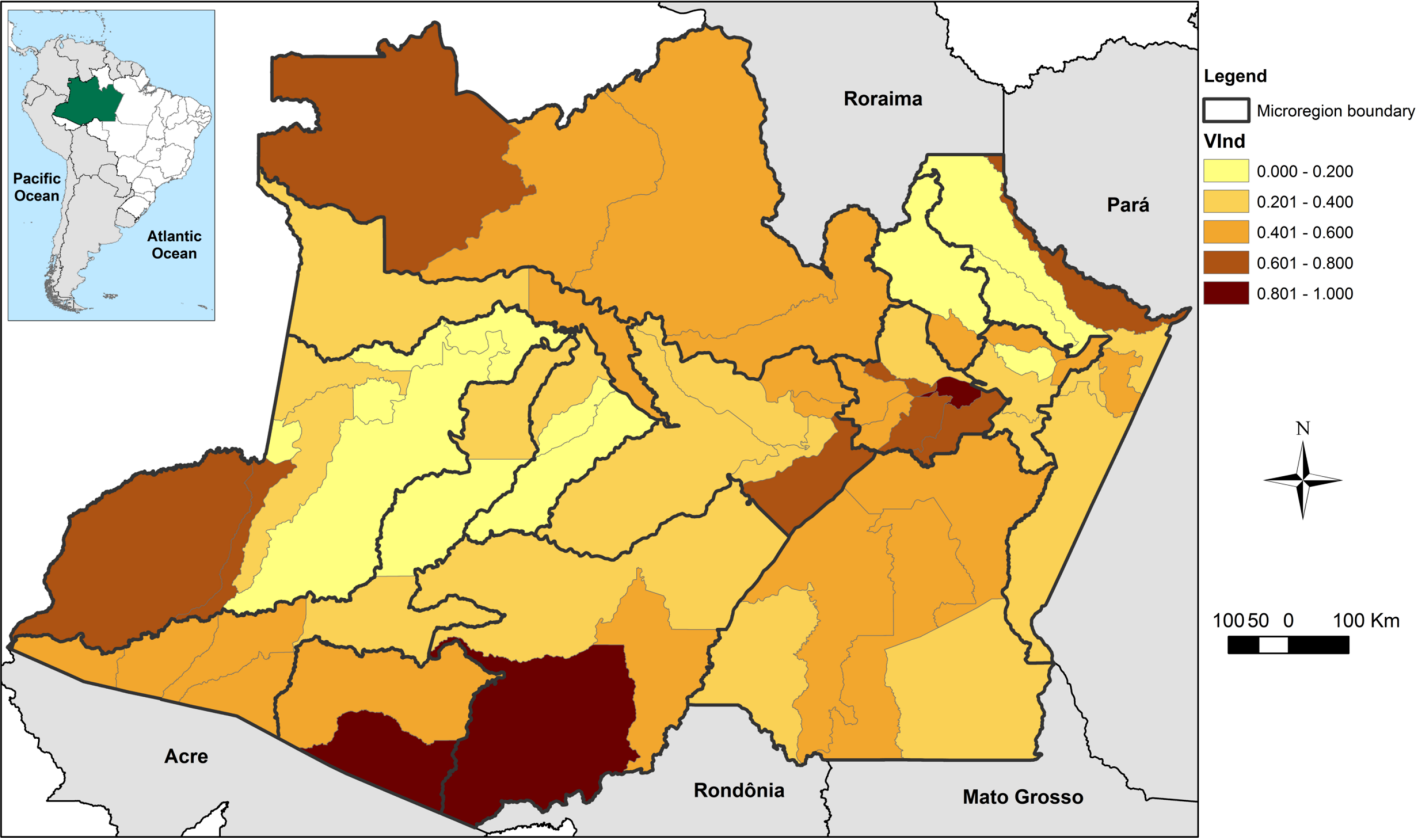
Map of the current Vulnerability Index (VInd) for the municipalities of the state of Amazonas.

The municipalities of the state of Amazonas were found to have medium current vulnerability; the average value of the Vulnerability Index (VInd) was 0.406. It is clear that the VInd was very heterogeneous throughout the state, with the central portion being the least vulnerable (lower VInd), while most of the southern, northern, and eastern regions, including some municipalities of the metropolitan region of Manaus, had higher VInd scores (Fig 5). The microregions of Purus, Manaus and Boca do Acre possessed the highest (> 0.560) average values of current vulnerability.

**Fig 6:**
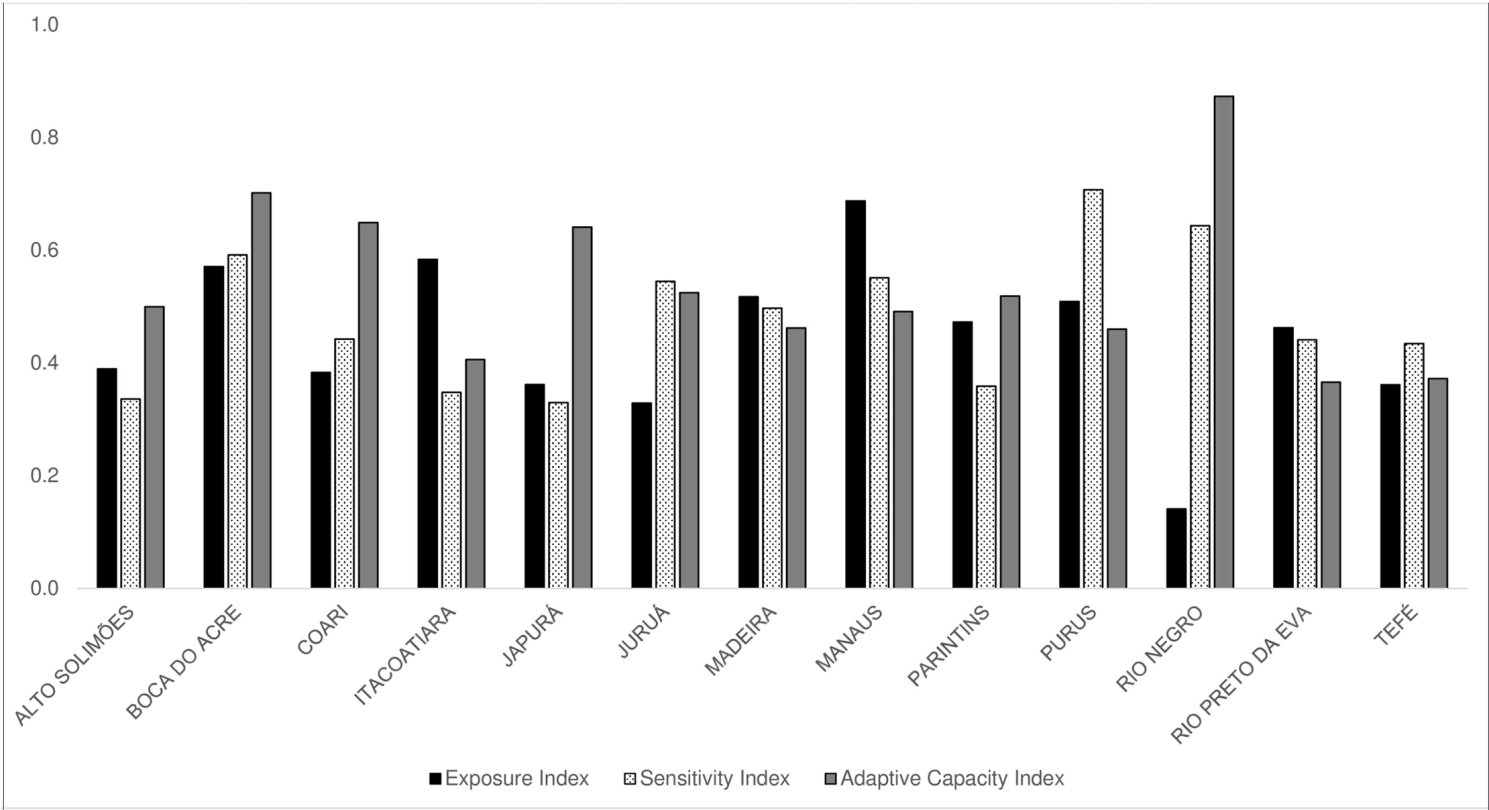
Values of the sub-indices that compose the current Vulnerability Index (VInd) of the microregions of Amazonas. Distribution of average values of exposure, sensitivity, and adaptive capacity indices for each microregion of the state of Amazonas.

The distribution of the exposure, sensitivity, and adaptive capacity indices for each microregion are shown in Fig 6. The exposure index was the main factor influencing current vulnerability (VInd) in the microregions of Rio Preto da Eva, Itacoatiara, Madeira, and Manaus, when compared to the sensitivity and adaptive capacity indices. In the Rio Preto da Eva, Madeira and Manaus microregions, this finding may be attributed to the greater occurrence and susceptibility to natural disasters, while in the Itacoatiara microregion, the vegetation cover and deforestation index had greater influence (S1 Table). The sensitivity had the greatest role in increasing the current vulnerability of the microregions of Juruá, Tefé, and Purus, due to the high indices observed for poverty conditions (PoI). Finally, the microregions where adaptive capacity were most important were Alto Solimões, Boca do Acre, Coari, Japurá, Parintins, and Rio Negro, all of which had high vulnerability values for the Sociopolitical Organization Index (SOI) and Socioecomic Structures Index (SEI).

**Fig 7:**
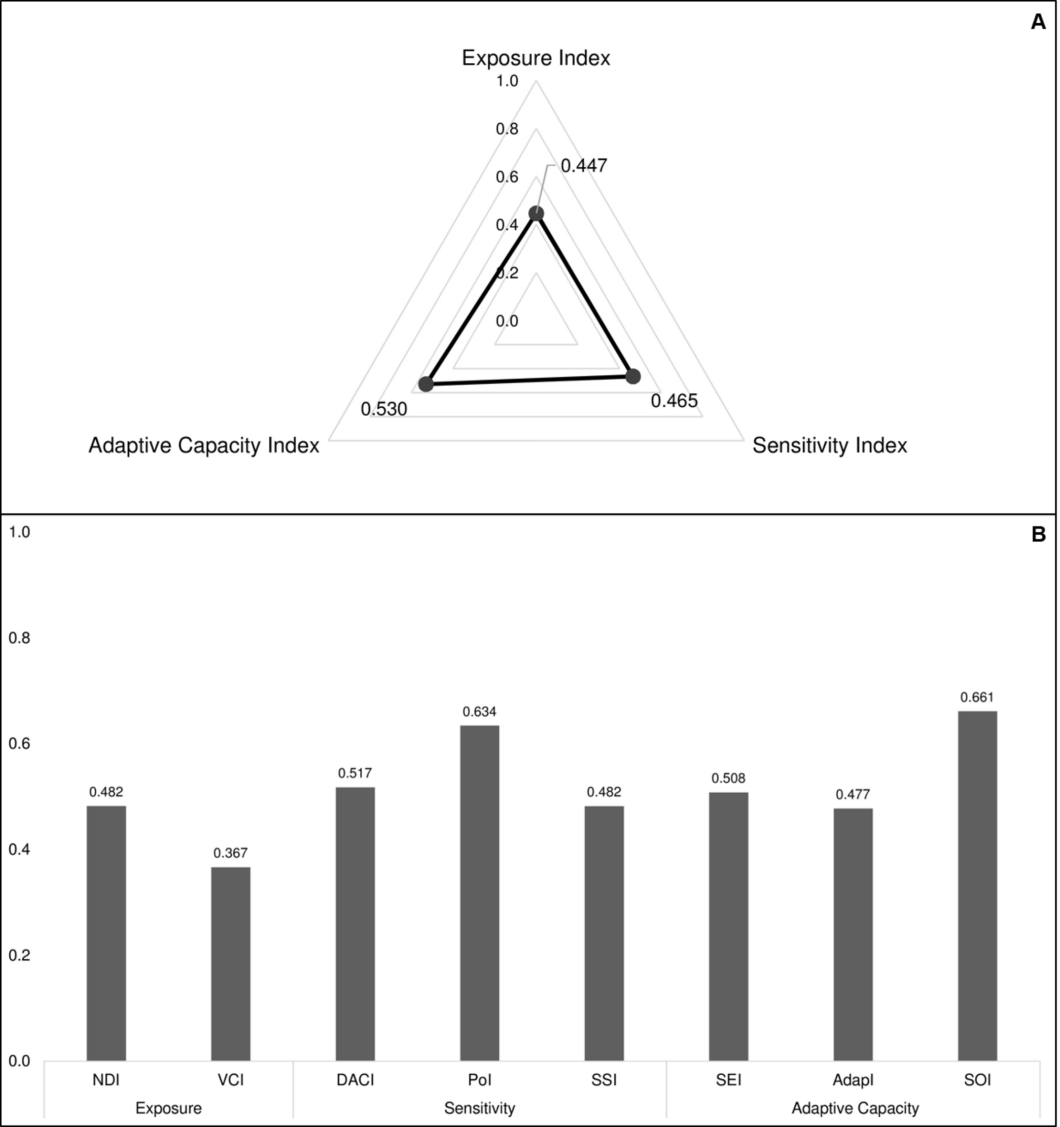
Values of the main indices that compose vulnerability, and each of their components. (A) Radar chart of the average values of the 62 municipalities in the core vulnerability indices—exposure, sensitivity, and adaptive capacity—demonstrating how they interact to build a unique profile for the population of Amazonas, Brazil. (B) Average values of each sub-index developed as a basis of the core indices of vulnerability. Abbreviations: NDI–natural disasters index; VCI–vegetation cover index; DACI–diseases associated to climate index; PoI–poverty index; SSI–sociodemographic sensitivity index; SEI—socioeconomic structures index; AdapI—institutions, services and infrastructure for adaptation index; SOI–sociopolitical organization index.

Fig 7A shows the average values of the key components of VInd. The municipalities demonstrated a poor performance in the Adaptive Capacity Index (0.530) followed by the Sensitivity Index (0.465), and by the Exposure Index (0.447). Fig 7B illustrates the performance of the municipalities, in average values, in the indices that compose exposure, sensitivity and adaptive capacity. The SOI was one of the components where the municipalities had the poorest results, followed by the PoI. The lowest mean values were observed in the Vegetation Cover Index (VCI), where the state showed a reduced vulnerability for almost all the municipalities.

**Fig 8:**
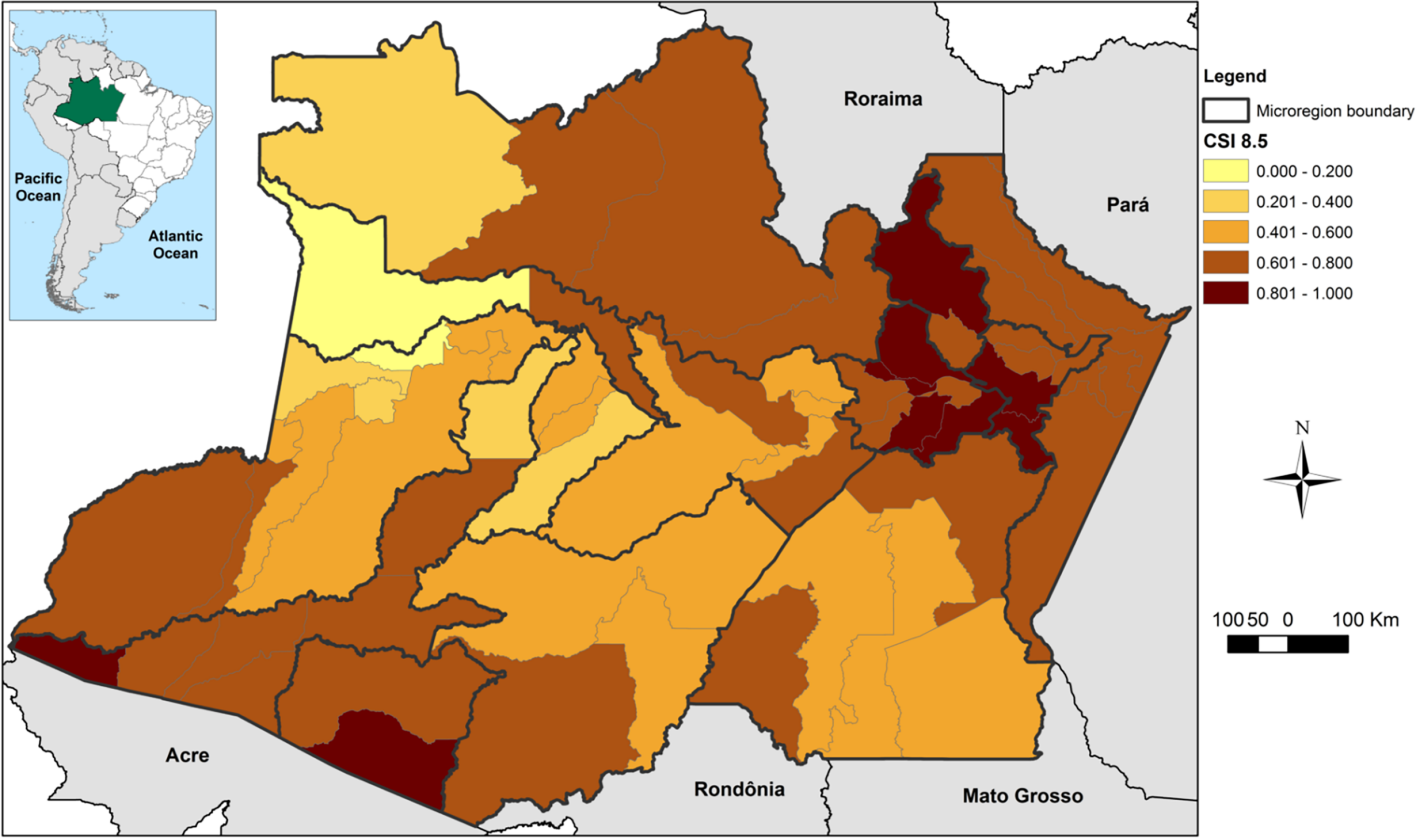
Representation of the Climate Scenario Index for the municipalities of the state of Amazonas, Brazil.

Climate change was represented by the Climate Scenario Index (CSI), which encompasses the anomalies of precipitation and temperature for the 2041–2070 period (baseline 1961–1990), considering a pessimistic emission scenario (RCP 8.5). Fig 8 maps how municipalities may be affected by future climate. The northern, northeastern and southwestern regions had the highest vulnerabilities to climate.

The temperature and precipitation indices for the emission scenario RCP 8.5 were used to develop the CSI, and its average value was considered medium-high (0.630). By disaggregating the components of CSI, both temperature and precipitation anomalies could be seen to have contributed similarly to the final value of the index. However, the value of the precipitation index (0.590) was slightly greater than that of the temperature index (0.581). In general, the climate change projections related to both parameters were not uniform for the state (S1–S6 Figs). Higher anomalies of maximum temperature were concentrated precisely in some regions (northeastern, southwestern) where CSI will be more prominent (S1 Fig). A similarity was observed between the distribution of the precipitation extremes (R95p and Rx5day) and the CSI for the northern and northeastern regions (S2 and S3 Figs). There was more homogeneity for the anomalies of minimum temperature, although the increases tended to be less pronounced, with the northeastern region again having the highest average increases for minimum temperature (S4 Fig). Regarding CDD, most of the state may show an increase in the number of days when the rain is below 1mm (S5 Fig), while total annual precipitation may show a large reduction in the eastern edge of the state and the southern region (S6 Fig). The state capital, Manaus, and its metropolitan region, located in the northeastern portion of Amazonas, as well as the northern and southwestern regions, were among the most vulnerable regarding climate (Fig 8).

**Fig 9:**
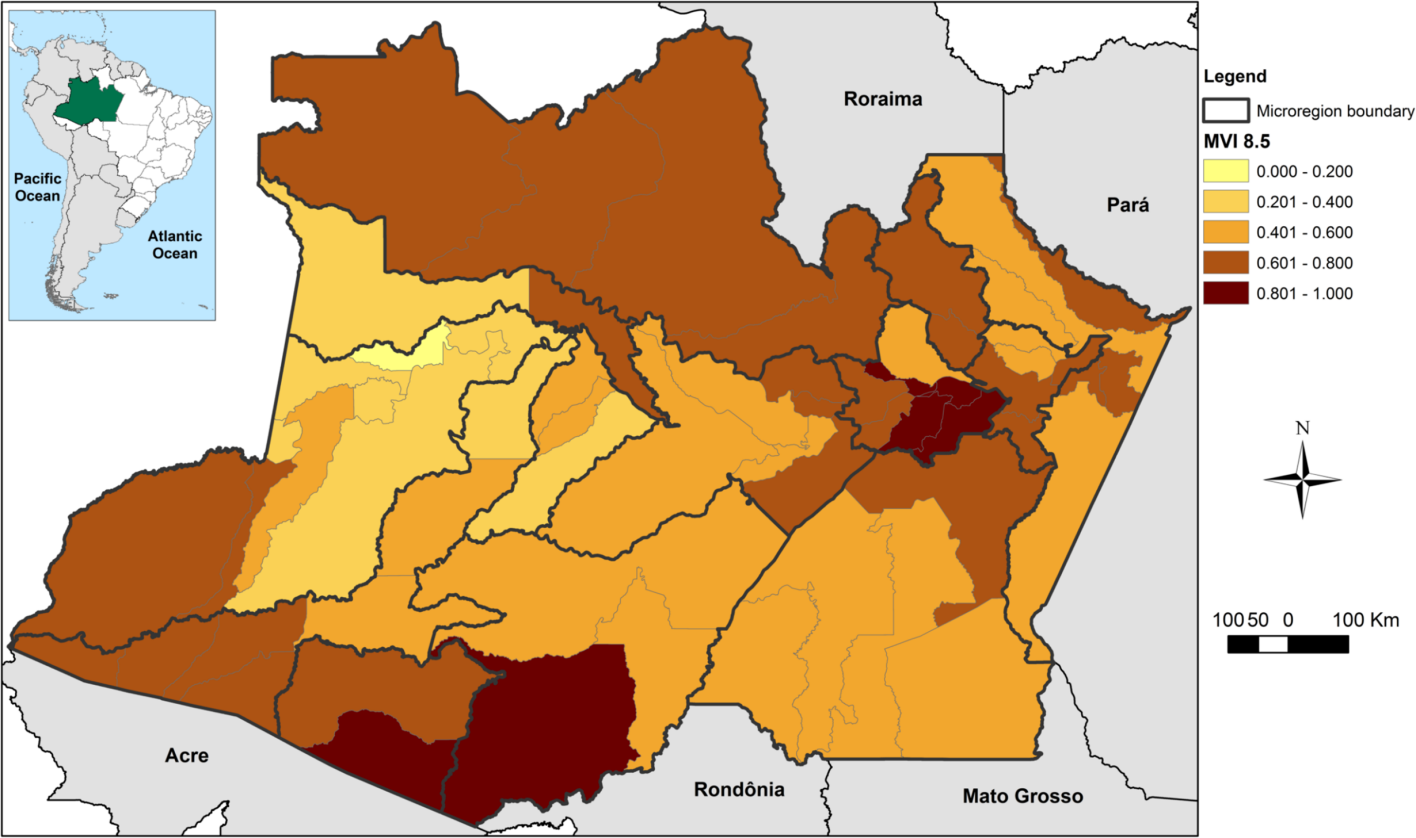
Municipal Vulnerability Index (MVI) of the state of Amazonas, Brazil. Representation of distribution of MVI values, considering the IPCC’s emission scenario RCP8.5.

The combination of current vulnerability (VInd) with the climate scenario (CSI) resulted in the Municipal Vulnerability Index (MVI). As demonstrated in Fig 9, the current vulnerability profile for the population of Amazonas (VInd–Fig 5) may be exacerbated by the climate in a more preponderant way in the northern, northeastern, extreme southern and southwestern regions.

**Fig 10:**
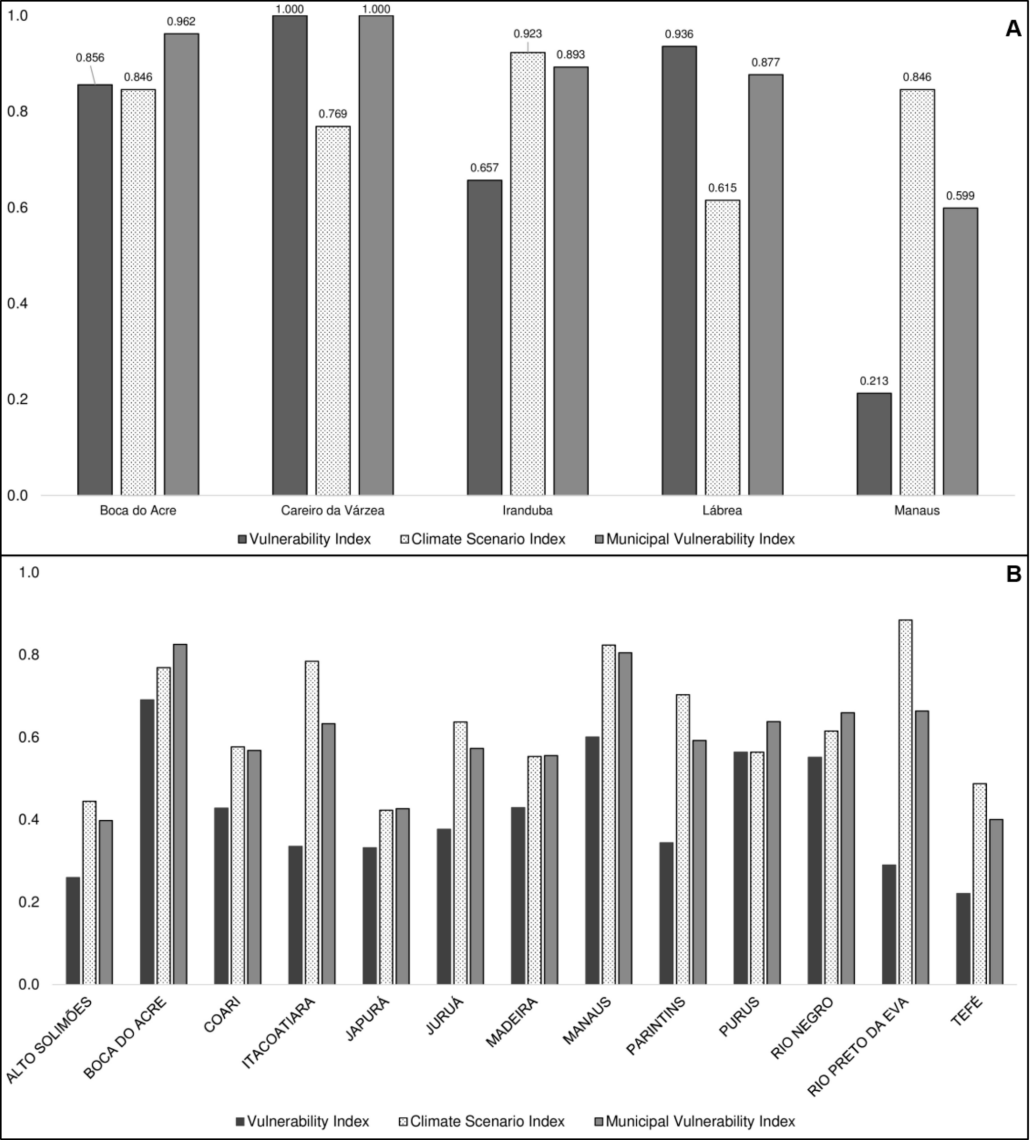
Distribution of the current Vulnerability Index, Climate Scenario Index, and Municipal Vulnerability Index. (A) Values of the indices referring to the most vulnerable municipalities and the Amazonas state capital, Manaus, Brazil, according to MVI. (B) Distribution of the indices, in average values, for the microregions of the state of Amazonas, Brazil.

The average value of MVI was 0.586. The municipalities with the highest MVIs were Careiro da Várzea (1.0) and Iranduba (0.893), both located in the Manaus metropolitan area; and Boca do Acre (0.962) and Lábrea (0.877), both located in the southern region. The capital, Manaus, which concentrates half of the inhabitants of the state, presented a medium MVI (0.599) (S2 Table). Fig 10A shows the values for the indices VInd and CSI for the most vulnerable municipalities, according to MVI, and for the state capital Manaus. It was noted that the indices interact in different ways in determining the final MVI of each municipality and microregion. However, when analyzing the values of vulnerability and climate scenario aggregated by microregion, it is clear that climate change projections represent the component with the greatest influence on the final vulnerability of the municipalities of Amazonas, represented here by MVI (Fig 10B).

## Discussion

### Insights for the state of Amazonas

The results of the present study demonstrate that current vulnerability, represented by the VInd, was quite heterogeneous, with the lowest values concentrated in the central region, and increasing vulnerability radiating outward towards the southern extreme, and eastern and northern regions. Some of the microregions identified as the most vulnerable by the VInd (i.e., Purus and Boca do Acre) were similar to those indicated by the Amazon Social Progress Index study [34] as having the lowest social progress in the state of Amazonas. Although the Amazon Social Progress Index did not consider a monetary dimension in its construction, as was the case with VInd, the overlap of microregions from the both studies is indicative of social and poverty issues as preponderant factors in determining the current vulnerability of the population of Amazonas.

Remarkably, these two aspects were those for which the municipalities of Amazonas had the worst performance, namely, the Poverty Index and the Sociopolitical Organization Index. The poverty issue, understood as deprivation of elements essential to a dignified life that go beyond the monetary issue, was addressed in this study using the Poverty Index, whose average value was the one of the highest among all indices that contributed to VInd. In addition to socioeconomic vulnerability being intrinsically linked to poverty condition, the assessment of this aspect is essential because it acts as a proxy for other issues that may increase the vulnerability of specific groups of a population, indicating, for example, those at higher risk of food insecurity or with limited abilities to adapt to future variability and climate change [72]. In this regard, the Northern Region of Brazil possesses much worse indicators related to child malnutrition, for example, than the other regions of the country, and the state of Amazonas is no exception. In recent decades the state has been facing a scenario of food insecurity, as evidenced by the high prevalence of childhood malnutrition, anemia, and hypovitaminosis [73–75].

Another important aspect that deserves to be highlighted is that economic growth has not reflected better living conditions for the population of the State. The watershed of Purus River, located in the southern portion of Amazonas, was the area that experienced the most economic growth in the State between 2002 and 2009 –an average rate of 94% growth in the Gross Domestic Product, with impetus coming mainly from agricultural and livestock activities [76]. However, this same region was among the most sensitive in PoI, probably due to a strong dependency on social assistance; the Bolsa Família benefit (a government aid) that is granted to around 67% of the families in the region [77].

The other index highlighted in the present study was that reflecting the issue of sociopolitical organization. This aspect is considered to be capable of contributing to satisfying popular demands in a variety of areas that have a direct impact on well-being, health, and social protection, thus enhancing the resilience of territories. Studies point out that different processes of construction and interaction of social capital are capable of increasing the resilience of a population [61–63]. However, the sociopolitical organization index of the present study demonstrated that the state of Amazonas lacks an organizational structure that allows its population to discuss the problems that affect communities in matters related to climate, environment, housing, and basic sanitation. In general, the presence of councils and consortia related to these and other sectors was observed to be precarious, including even the state capital, Manaus.

In this sense, it is necessary to value the internal capacity that traditional populations possess to organize themselves and to overcome adversities posed by climatic events, which have been characteristically imprinted by the seasonality of the rivers in the riverine way of life. Studying the 1999 flood in the Reserva de Desenvolvimento Sustentável de Mamirauá (Mamirauá Sustainable Development Unit), a protected area along the Solimões River, Moura and Peres [78] observed that the problems faced were solved based on a strong level of solidarity established among the residents of the community to work around the needs unmet by the state. Although only 44% of the existing communities in the reserve and their surroundings received some kind of governmental aid, most of the families (70%) decided to continue living in their residences, which varied according to the proximity of urban centers. These facts show that whatever initiative is aimed at mitigating and reducing the impacts of climate on these populations, it should focus on the sense of belonging observed between traditional populations and their socio-productive space, without which adaptive actions can be undermined for lack of adhesion and recognition of local peculiarities.

The above-mentioned facts demonstrate that the key components of vulnerability—sensitivity and adaptive capacity, which are the components to which the poverty and socio-political organization indices are related–are the most important factors in need of improvement in order to reduce the vulnerability of the Amazonas population to future climate variability and change. Although the Amazon region has experienced increased frequency of extreme events over the last few decades, this was not reflected in higher exposure rates in the present study, possibly because the environmental aspect (vegetation cover index and deforestation) has helped to modulate the effects of disasters in the state.

Natural disasters, included in the exposure component, was found to have a medium value, although other studies have demonstrated the susceptibility of the Amazon system to these phenomena, especially metropolitan areas [51,52,79–83]. The more frequent and intense extreme natural events reported in recent years has resulted in disasters of great magnitude due to the scarcity of safe water and food and the displacement of people throughout the region [35,36,48,49,79,84]. A recent study on the vulnerability of Brazilian municipalities to the increase in flood and landslide events showed that the western portion of Amazonas can anticipate increased flood-related vulnerability ranging from 4% to more than 30%, considering the HADGEM-ES climatic model in a pessimistic emission scenario (RCP8.5) [21]. As for landslides, starting from the same scenario and climatic model, the study showed that the entire state of Amazonas, except for small extensions in the extreme north and east, could experience increased vulnerability until the end of the century of between 3% and more of 30%.

Analyzing these data from the perspective of the results found in the Natural Disaster Index of the present study, found that most of the state may, in the near future, suffer doubly the impacts of both flood and landslide disasters, given the position of vulnerability to disasters already present in the southern and eastern portions of the state, which will thus tend to be increased. Pereira et al. [79], studying the risk of disasters in the basin of Purus River, observed that the municipality of Boca do Acre was an area of high threat for disasters associated with increased precipitation. Simultaneously, this same municipality, together with Canutama, also proved to be highly threatened by the occurrence of disasters associated with the intense reduction of precipitation. Boca do Acre was also one of the municipalities most vulnerable to the occurrence of disasters according to Natural Disaster Index, revealing that this municipality needs timely preventive actions to be implemented soon in order to avoid material and human damage in the coming decades.

Regarding climate, the CSI showed the northern and northeastern regions and the southwest portion of the state to be more vulnerable. In general, the projections of climate change for the region are inconsistent due to complex thermodynamic ocean-atmosphere interactions and the different results presented by different climate models [85–87]. Some models have projected rainier climates whereas others have projected drier climates for the Amazon Basin, though, the consensus is of an increase in mean temperature and reduction of rainfall for most of the Amazon territory–the same was found in the present survey by CSI [25,82,88,89]. The impacts may be severe since climate change might turn extreme events, like the droughts of 2005 and 2010, into the rule more than an exception. The close relationship and dependence of the populations of Amazonas to the hydrological regime has raised concerns about food security, mobility, and water potability, among others. It is noteworthy that the regions of the state where climate change has been most prominent are also those associated with higher disaster rates (southwest and northeastern regions), the poorest social indicators (northern and southwestern) and the highest population density in the state (metropolitan area) [90,91]. The long-term consequences of maintaining this socio-environmental pattern might be the loss of economic, human, and natural assets.

The climatic issue also poses a challenge to the conservation of the tropical rainforest of the Amazon. Recent estimates suggest that the combination of deforestation and global climate change could cause an increase in the occurrence of forest fires in the Amazon of up to 50% by 2050, creating a cycle of degradation and biodiversity loss [92]. Studies report thresholds that should not be exceeded in order to ensure the maintenance of the Amazon rainforest–up to 40% deforestation and a temperature rise of 3°C to 4°C [44,93–95]. In the Amazon, there exists a nonlinear interaction between several drivers that has culminated in the environmental changes that have been observed–the region has warmed 1°C in the last 60 years and lost about 20% of its original vegetation cover [96]. Among these drivers are climate change and land use due to global warming and deforestation, which in turn induce a greater frequency of extreme climatic events and forest fires, increasing the vulnerability of the systems of the Amazon [96]. These studies show how vegetation suppression and environmental change interact synergistically in tropical areas, which places the northeast and southern regions of the Amazonas on highest alert. The northeast portion because: 1) it was the one region that possessed many microregions–Manaus, Itacoatiara and Rio Preto da Eva–with the highest values for the indicator of vegetation cover and deforestation; and 2) it could experience the greatest climate change according to the CSI. The southern portion, comprising the microregions of Madeira and Purus, deserves special mention because: 1) according to reports of the Instituto do Homem e Meio Ambiente da Amazônia (IMAZON), it contains a concentration of municipalities in which deforestation increased the most in 2016, in addition to suffering intense pressure from the agricultural frontier, which is expanding from neighboring states; and 2) it is one of the places where climate change may be more prominent, according to the CSI.

Ultimately, the MVI demonstrated the northern, northeastern, and south-southwestern regions of the state to be the most vulnerable to future climate change, considering current socio-environmental conditions. However, the spatial distribution of vulnerability was quite different between MVI and CSI, suggesting that the areas most impacted by climate are not, necessarily, more vulnerable from the socio-environmental perspective. These findings are corroborated by other studies about vulnerability to climate in Brazil [2,25,28]. The microregions of Purus and Boca do Acre, in the south-southwestern region, had similar values for vulnerability and climate indices, whereas other regions, such as Rio Preto da Eva, Itacoatiara, and Manaus (northeastern), showed the climate index to be more important for the definition of MVI. Regarding Manaus, Torres et al. [25] identified it as a socio-climatic hotspot, mainly due to the high values observed in the climate index and the low human development of the peripheries, which may translate into greater incidence of diseases and/or more frequent landslides and floods related to high population density.

It is important to highlight that the reduced vulnerability values observed for the municipalities in the central region of the state, as well as for the Madeira microregion, does not mean that these territories do not require actions to enhance their adaptive capacity and resilience. Thus, for microregions that have municipalities in the central parts of the state–Coari, Tefé, Japurá and Juruá –investment is essential to reduce poverty together with the improvement of socio-political articulations, since the climatic aspect was not decisive in determining the vulnerability of these human populations to the climate change.

### Broader implications

Although the conditions that affect people's vulnerability profiles are determined by local interactions, which is reflected in dramatically different responses throughout the world to the same climate stimulus, some conditions are considered “generic” determinants of vulnerability and are closely related to developmental issues (e.g., poverty, governance, health) [17]. Interestingly, findings from the present study show that some generic issues, namely poverty (sensitivity index) and sociopolitical organization (adaptive capacity index), as well as the climate scenario, were the main aspects influencing vulnerability of the municipalities of Amazonas (MVI). These generic aspects demonstrate that, although vulnerability assessments are often based on intricate methods of analysis, due in part to the complexity of interactions, short-term actions to reduce vulnerability in developing countries should focus on improving quality of life and coping skills of the population, along with tackling problems with risk on a local-basis. This aspect is reviewed by Brooks et al [17], who state that addressing issues of health, education and governance would improve adaptation, specific measures and technologies should also be promoted among specific localities or population groups.

The aspects of governance and institutional capacity, for example, are considered a challenge in vulnerability assessments and for the implementation of effective strategies for adaptation at the local level. On the one hand, governance and social capital are difficult to measure because they are highly variable factors among populations, and present structural dimensions that are still barely visible or poorly understood [11,97]. On the other hand, the most vulnerable population groups are often excluded from decision-making processes, and marginalization itself is a trigger for situations of vulnerability [55]. In Brazil, there is evidence of positive impacts from the participation of civil society in municipal councils and the cooperation between local governments through consortia for provisioning basic services to the population [98–101]. On a local-basis, these agencies represent possible channels of communication between the population and the public power, established by the law, which may assist in voice and accountability issues. This shows the key role of those institutions in meeting popular demands and better allocating public resources. Moreover, these findings reiterate that the existence of these institutions is a viable step towards articulating the best policy of the population to the local government. However, there is no doubt that the present method does not allow a thorough evaluation of the quality of the institutional articulation promoted between these bodies and the population, which calls for new ways of thinking about the evaluation of aspects of governance as capable of increasing the adaptive capacity in local contexts.

Regarding climate, the results presented here highlight that the incorporation of CSI substantially rose the vulnerability scores from the VInd to the MVI, showing that the climatic issue needs to be specifically addressed and is of crucial importance in differentiating the relative vulnerability to climate change presented by the municipalities. O’brien et al [4] have shown that the districts with greater sensitivity to climate in India were not those considered more vulnerable, mainly due to varying levels of adaptive capacity. Using the Socio-climatic Vulnerability Index, Torres et al [25] reported similar findings for some metropolitan areas in Brazil. These authors showed that high population densities were an important factor in making cities with moderate expected climate change highly vulnerable to future climate changes.

As demonstrated by Engle & Lemos [11], from a policy perspective, decision makers are interested in identifying specific characteristics of the system on which they can work. Thus, the analysis of the vulnerability indicators proposed herein, and in other studies, lead to policy recommendations that depend directly on the local perspective [2,8,14,28,71,84]. These relativizations are important to ensuring that decision makers can use the information produced in a relevant and practical way. In the present work, the disaggregation of the indices allows for a detailed analysis to identify specific actions and priority areas for intervention, mainly through the mapping of the results, since stakeholders and decision makers can systematically identify and evaluate the spatial distribution of the likely vulnerabilities. The disaggregation of the indices allows for a detailed analysis to identify specific actions and priority areas for intervention. For example, the results for the microregion of Boca do Acre showed that it possessed the highest current vulnerability status due to poor performance in adaptive capacity, particularly due to low socio-political organization (S1 Table). On the other hand, the microregion of Tefé presented the lowest value for the present vulnerability, but with a worse or similar performance in the components of the sensitivity index when compared to the microregion of Boca do Acre, especially regarding the diseases associated with climate index. This possibility of disaggregating the indices and analyzing them individually, either by microregion or by municipality, makes the MVI a useful tool for planning actions in the medium and long term that are adapted to the local reality. In addition, the use of systematic information allows constantly feeding the database and to evaluate the modification of the conditions of vulnerability and/or actions implemented over time.

These remarks show that broad comparisons of the results of different vulnerability studies are a risky task in the face of the profusion of concepts and complexity surrounding the theme, which favors the proliferation of approaches without methodological and/or conceptual uniqueness [9,102]. However, Cannon et al [97] demonstrated the applicability of a method called the analysis of capacities and vulnerabilities (CVA), which was proposed by a non-governmental institution in 1980. The CVA has since then been absorbed by other methodological approaches, mainly in the Philippines, where it was progressively revised and applied for more than a decade to the theme of disasters. In addition, these authors also showed that although the focus and availability of data in each study using CVA varied greatly, the method was able to assess the vulnerability and capabilities of the populations studied with considerable breadth and depth. Likewise, in Brazil, there are studies applying and improving the same conceptual model of vulnerability proposed, initially, to evaluate the vulnerability of the municipalities of the state of Rio de Janeiro to climate [2], to other regions of the country, such as the municipalities of the state of Minas Gerais [28,58] and the Paraguay River Basin [58]. Although several methodologies are available to carry out vulnerability assessments (i.e. qualitative diagnostics, future model simulations, statistical analysis), indicators-based approach have been presenting satisfactory results in evaluating the vulnerability of specific sectors (i.e. agriculture) and populations to climate change [71]. In a review of 35 vulnerability case studies carried out across the globe at different scales using the indictors-based approach, Sabelli [71] demonstrated that this kind of evaluation is useful in countries with significantly lack of data, and technical and financial resources to undertake more complex modeling simulations, mainly at small scales, showing the practical application of this approach.

This demonstrates that although the MVI methodology was developed for the Amazon, its adaptation to other regions of Brazil is possible because it encompasses both the main dimensions of vulnerability and the interactions between its components—exposure, sensitivity and adaptive capacity—with the perspective of future climate change. This demonstrate its reproducibility and simple approach comparing to other methods of vulnerability evaluations, allowing to include abstract concepts in a quantitative analysis. Its applicability is possible as long as local conditions are considered, the variables or indicators are adapted to regional peculiarities, and the specificity of scale is respected, which, methodologically, can limit its use [103].

Thus, it is possible that MVI can be used simultaneously as a diagnostic and planning tool, in the same way that Cannon et al [97] demonstrated for CVA. As a diagnostic tool, the indices created can help to (i) understand the problems and underlying the causes of vulnerability, and to (ii) highlight priority areas for reducing vulnerability, as well as what actions should be taken (e.g. reducing deforestation, improving social indicators). As a planning tool, the MVI methodology may, in addition to helping prioritize actions, allow the dynamic monitoring of proposed future adaptation actions, since the constructed indices can be systematically updated.

## Conclusions

The results presented here are a pioneering attempt to evaluate the social-environmental and health vulnerability of the population of the state of Amazonas to the impacts of climate change. The major contributions of this study are the development of a set of indicators adapted to the reality of Brazil and the mapping of the vulnerabilities of the municipalities of the state of Amazonas using a climatic perspective. The results have shown that a first step towards making the population of Amazonas better prepared to cope with climate impacts would be the improvement of their life and health conditions, as poverty was one of the factors that most influenced the current vulnerability of the municipalities.

Another important step towards improving adaptive capacity is to invest in the infrastructure of municipalities, such as health care networks and disaster preparedness, since drought and flood events for the Amazon region may become more frequent and intense. Although this does not represent a greater risk of death to the local population, which is already adapted in many ways, a greater severity of climate events implies a threat to food and the nutritional security of the region. The dependency of the smallholder on the river flood regime, which determines not only the appropriate time for farming and the viable crops, but also the flow of goods between the small cities and Manaus, is evidence of how harmful a change in rainfall and hydrological patterns of the Amazon Basin could be.

The methodology applied was developed to be simple and easy to implement so that it could be appropriated by decision-makers at municipal and state levels. The methodology allowed the interpretation of vulnerability in a synthetic manner and the targeting of resources and actions according to regional peculiarities. In the future, this method is likely to be applied to other Amazonian states of Brazil that share the same socio-spatial characteristics as the municipalities of the state of Amazonas.

## Supporting information

S1 Fig Representation of the average anomaly of maximum temperature for the state of Amazonas, Brazil. The anomalies (°C) were calculated considering the current slice as 1961–1990 and the future slice as 2041–2070 from a pessimistic emission scenario, the IPCC’s RCP8.5.
(TIF)

S2 Fig Representation of the R95p anomaly, which represents extreme precipitation above 95 percentile, for the state of Amazonas, Brazil. The anomaly was calculated considering the percentage difference between the current slice (1961–1990) and the future slice (2041–2070) from a pessimistic emission scenario, the IPCC’s RCP8.5.
(TIF)

S3 Fig Representation of the Rx5day anomaly, which represents the maximum precipitation accumulated in five days, for the state of Amazonas, Brazil. The anomaly was calculated considering the percentage difference between the current slice (1961–1990) and the future slice (2041–2070) from a pessimistic emission scenario, the IPCC’s RCP8.5.
(TIF)

S4 Fig Representation of the average anomaly of minimum temperature for the state of Amazonas, Brazil. The anomalies (°C) were calculated considering the current slice as 1961–1990 and the future slice as 2041–2070 from a pessimistic emission scenario, the IPCC’s RCP8.5.
(TIF)

S5 Fig Representation of the CDD anomaly–consecutive dry days—for the state of Amazonas, Brazil. The anomaly was calculated considering the percentage difference between the current slice (1961–1990) and the future slice (2041–2070) from a pessimistic emission scenario, the IPCC’s RCP8.5.
(TIF)

S6 Fig Representation of the total precipitation anomaly for the state of Amazonas, Brazil. The anomaly was calculated considering the percentage difference between the current slice (1961–1990) and the future slice (2041–2070) from a pessimistic emission scenario, the IPCC’s RCP8.5.
(TIF)

S1 Table Average values of the main indices and sub-indices that composed the Municipal Vulnerability Index for the microregions of the state of Amazonas, Brazil. (DOCX)

S2 Table Values of the main indices and sub-indices, by municipality, that composed the Municipal Vulnerability Index of the state of Amazonas, Brazil. (DOCX)

S3 Table Raw values of the variables used to compose the exposure index of the municipalities of the state of Amazonas, Brazil. (DOCX)

S4 Table Raw values of the variables used to compose the sensitivity index of the municipalities of the state of Amazonas, Brazil. (DOCX)

S5 Table Raw values and information on the variables that composed the Adaptive Capacity Index of the municipalities of the state of Amazonas, Brazil. (DOCX)

S6 Table Raw values of the climatic parameters used to compose the climatic scenario index of the municipalities of the state of Amazonas, Brazil. (DOCX)

S7 Table Percentiles used to assign scores to the quantitative variables. (DOCX)

S8 Table Assignment of values to the qualitative variables used to perform the institutions, services, and Infrastructure for Adaptation Index (AdapI). (DOCX)

S1 Appendix A brief description of the microregions of the state of Amazonas. (DOCX)
