# Supplementary figures and images for: Mapping human vulnerability to climate change in the Brazilian Amazon: The construction of a municipal vulnerability index
Source: PLoS One. 2018 Feb 14;13(2):e0190808. doi: 10.1371/journal.pone.0190808 (PMC5812563; doi:10.1371/journal.pone.0190808)

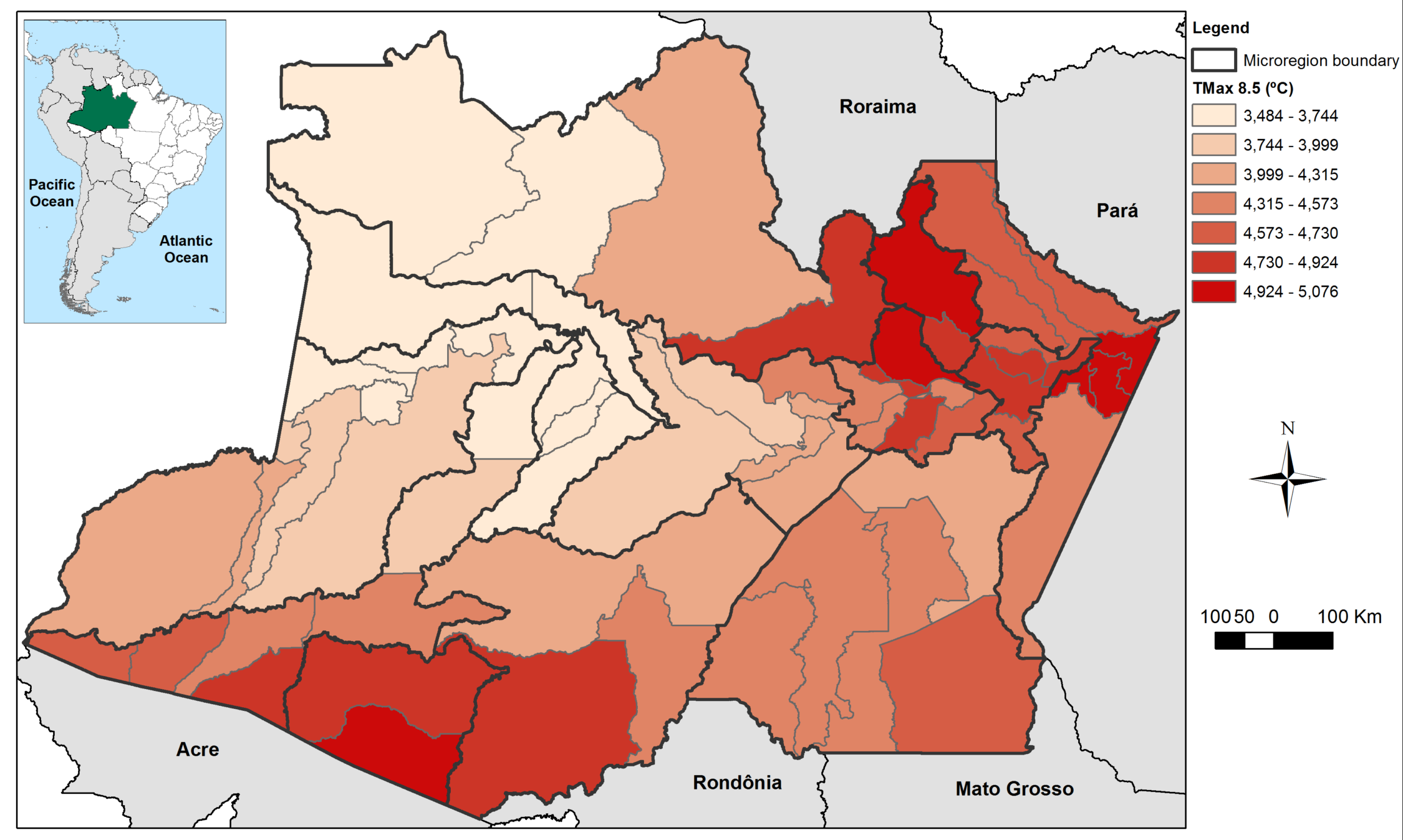

Supplement: S1 Fig — The anomalies (°C) were calculated considering the current slice as 1961–1990 and the future slice as 2041–2070 from a pessimistic emission scenario, the IPCC’s RCP8.5. (TIF) [file pone.0190808.s001.tif]

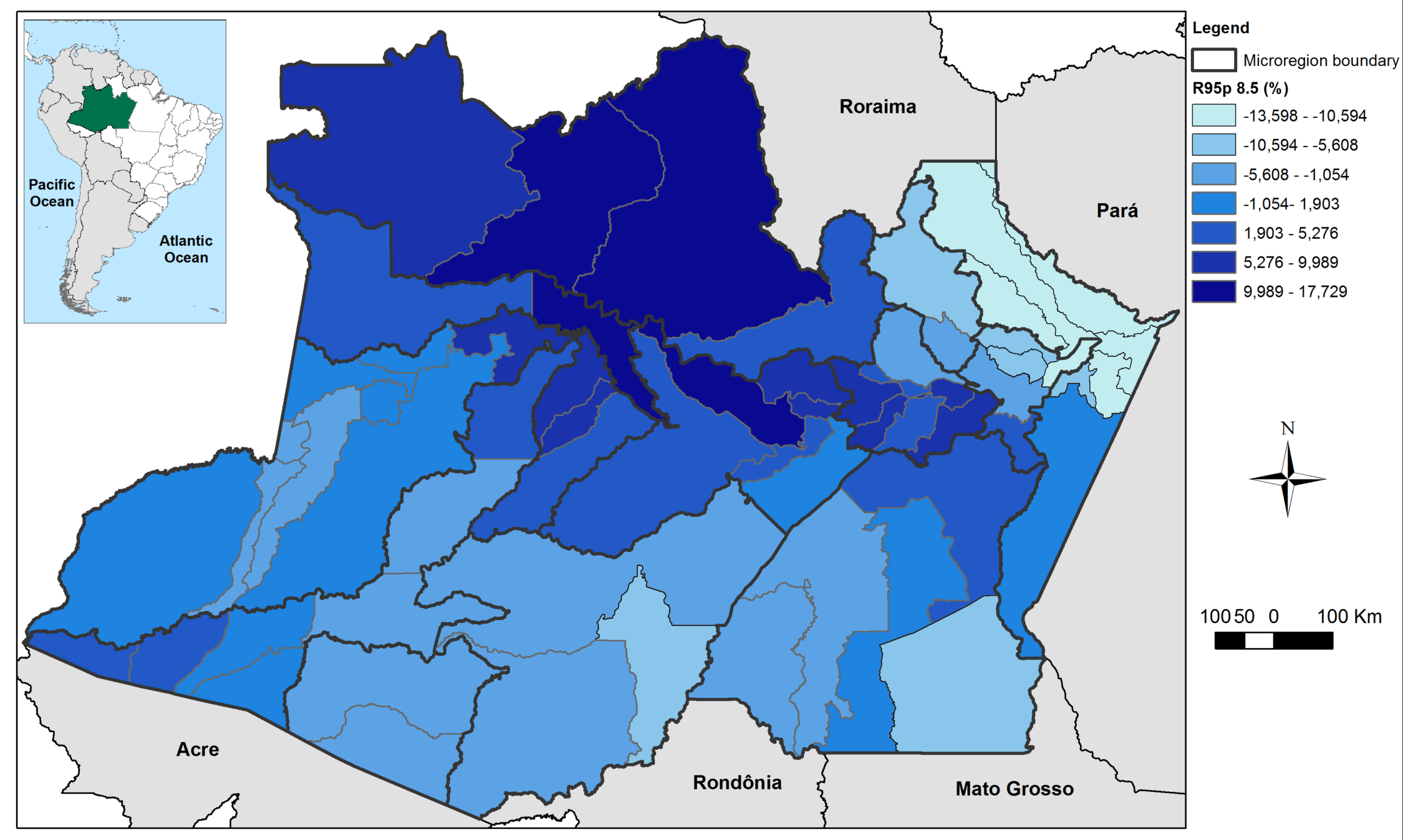

Supplement: S2 Fig — The anomaly was calculated considering the percentage difference between the current slice (1961–1990) and the future slice (2041–2070) from a pessimistic emission scenario, the IPCC’s RCP8.5. (TIF) [file pone.0190808.s002.tif]

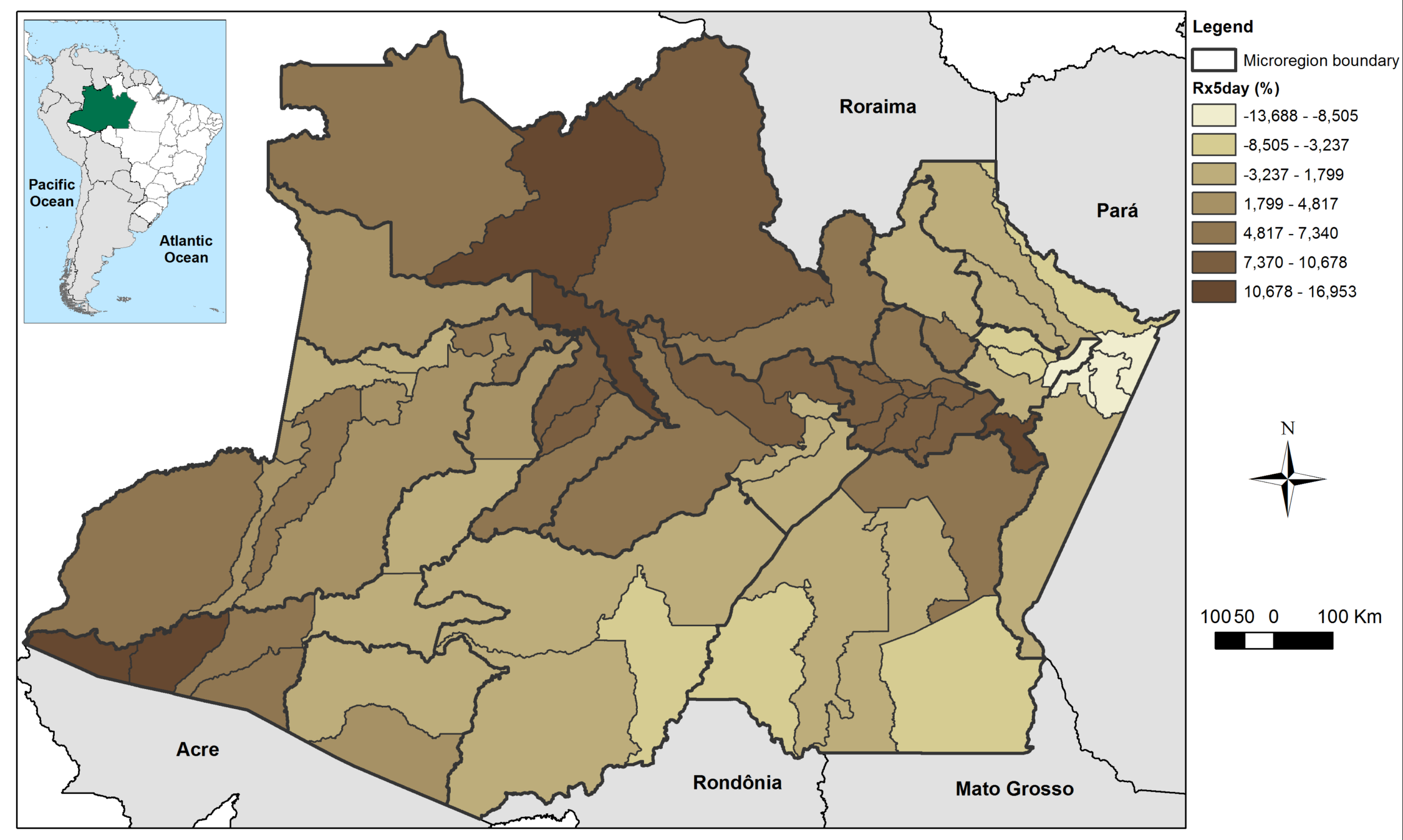

Supplement: S3 Fig — The anomaly was calculated considering the percentage difference between the current slice (1961–1990) and the future slice (2041–2070) from a pessimistic emission scenario, the IPCC’s RCP8.5. (TIF) [file pone.0190808.s003.tif]

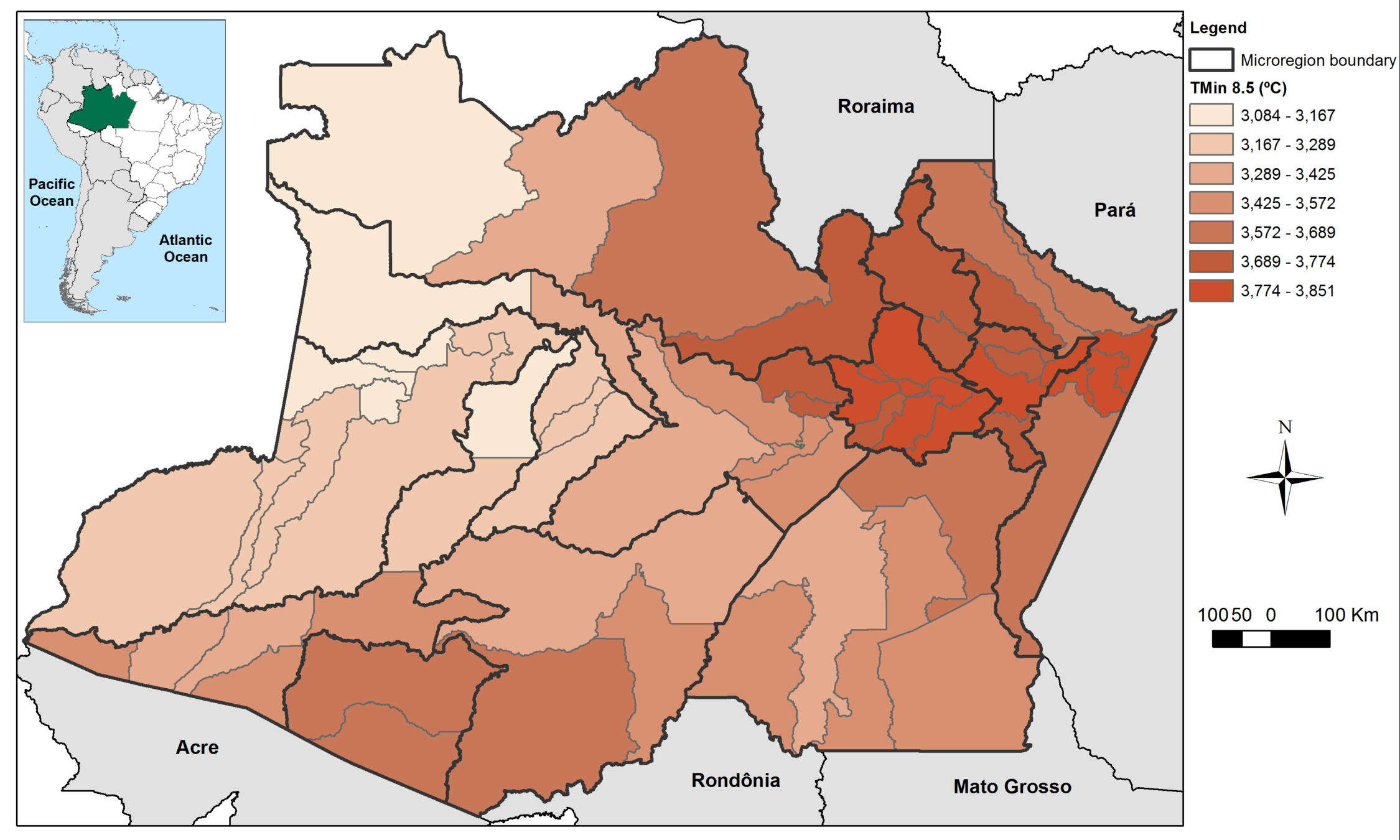

Supplement: S4 Fig — The anomalies (°C) were calculated considering the current slice as 1961–1990 and the future slice as 2041–2070 from a pessimistic emission scenario, the IPCC’s RCP8.5. (TIF) [file pone.0190808.s004.tif]

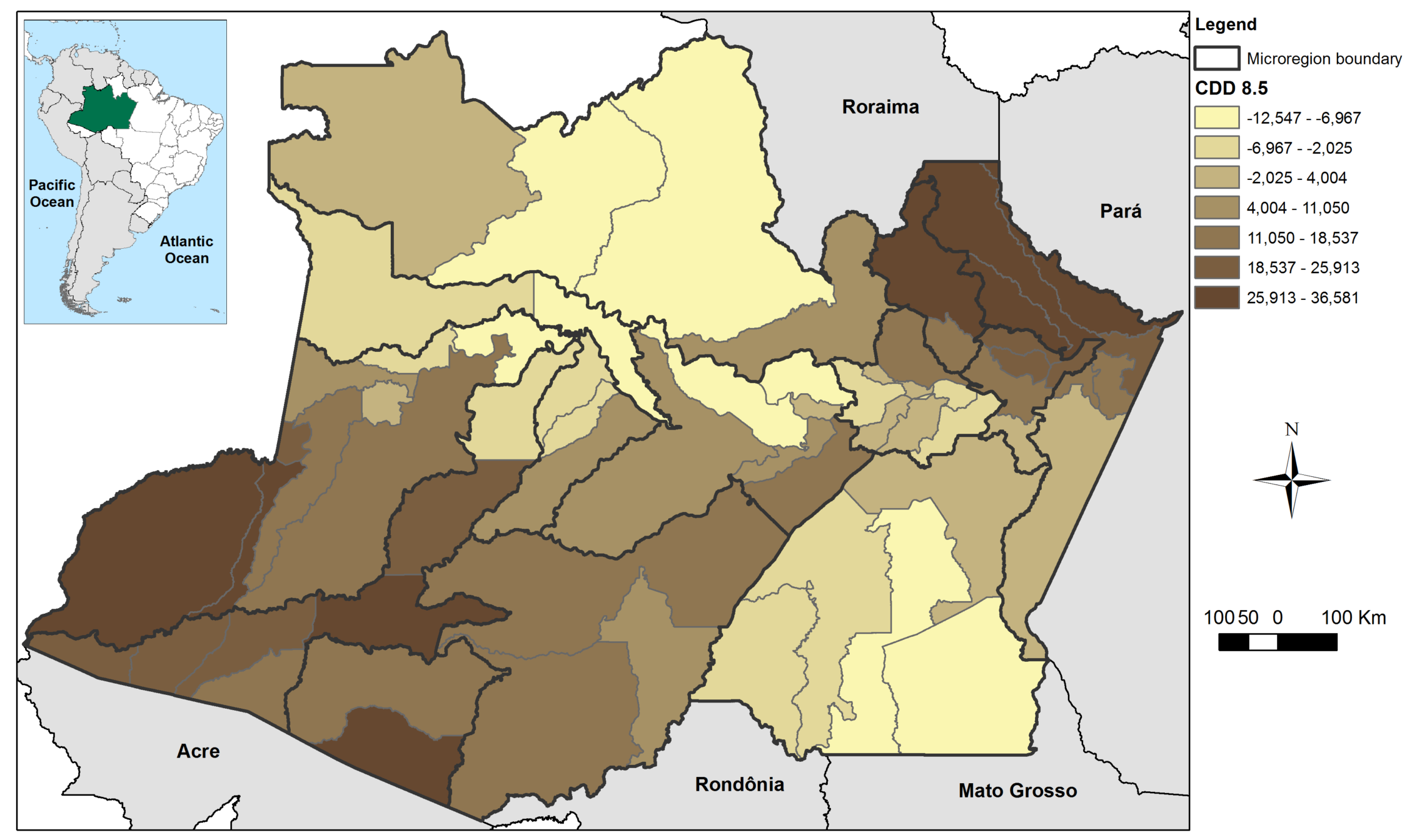

Supplement: S5 Fig — The anomaly was calculated considering the percentage difference between the current slice (1961–1990) and the future slice (2041–2070) from a pessimistic emission scenario, the IPCC’s RCP8.5. (TIF) [file pone.0190808.s005.tif]

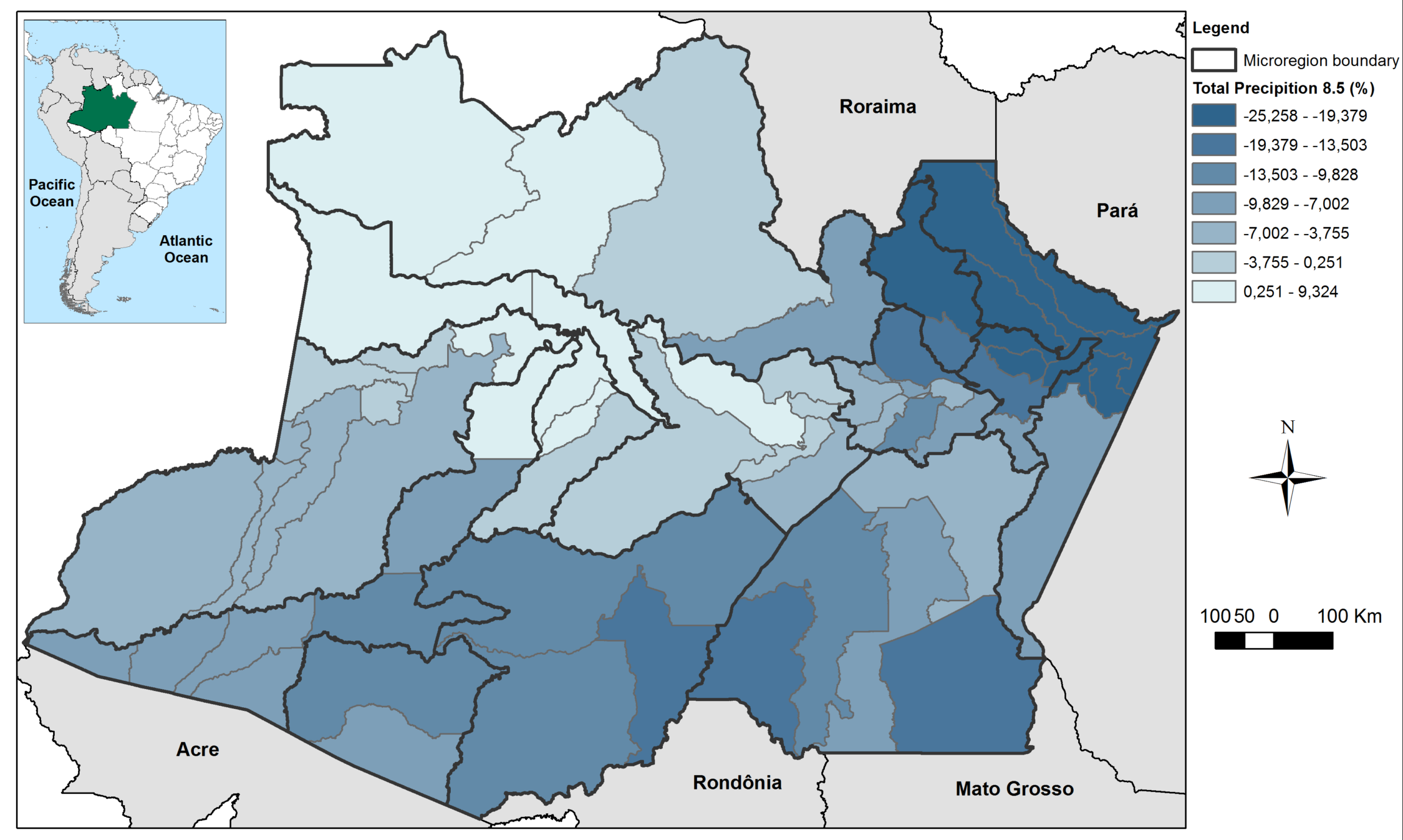

Supplement: S6 Fig — The anomaly was calculated considering the percentage difference between the current slice (1961–1990) and the future slice (2041–2070) from a pessimistic emission scenario, the IPCC’s RCP8.5. (TIF) [file pone.0190808.s006.tif]
