# Supplementary material for: Mapping human vulnerability to climate change in the Brazilian Amazon: The construction of a municipal vulnerability index
Source: PLoS One. 2018 Feb 14;13(2):e0190808. doi: 10.1371/journal.pone.0190808 (PMC5812563; doi:10.1371/journal.pone.0190808)
Supplement: S1 Table — (DOCX) [file pone.0190808.s007.docx]

**S1 Table. Average values of the main indices and sub-indices that composed the Municipal Vulnerability Index for the microregions of the state of Amazonas, Brazil.**

| **INDEX** | **MICROREGION** | | | | | | | | | | | | |
| --- | --- | --- | --- | --- | --- | --- | --- | --- | --- | --- | --- | --- | --- |
|  | **Alto Solimões** | **Boca do Acre** | **Coari** | **Itacoatiara** | **Japurá** | **Juruá** | **Madeira** | **Manaus** | **Parintins** | **Purus** | **Rio Negro** | **Rio preto** | **Tefé** |
| **Index of Natural Disasters** | 0.422 | 0.800 | 0.466 | 0.480 | 0.400 | 0.400 | 0.640 | 0.714 | 0.429 | 0.633 | 0.125 | 0.450 | 0.400 |
| **Vegetation Cover Index** | 0.318 | 0.286 | 0.262 | 0.629 | 0.286 | 0.225 | 0.343 | 0.592 | 0.469 | 0.334 | 0.143 | 0.429 | 0.286 |
| **Exposure Index** | 0.389 | 0.571 | 0.383 | 0.583 | 0.362 | 0.329 | 0.517 | 0.687 | 0.473 | 0.509 | 0.141 | 0.463 | 0.361 |
| **Diseases Associated to Climate Index** | 0.375 | 0.483 | 0.483 | 0.446 | 0.411 | 0.400 | 0.686 | 0.637 | 0.401 | 0.607 | 0.784 | 0.824 | 0.565 |
| **Poverty Index** | 0.736 | 0.782 | 0.625 | 0.438 | 0.750 | 0.848 | 0.588 | 0.464 | 0.527 | 0.834 | 0.719 | 0.282 | 0.646 |
| **Sociodemographic Sensitivity Index** | 0.338 | 0.552 | 0.493 | 0.582 | 0.279 | 0.500 | 0.407 | 0.656 | 0.554 | 0.542 | 0.387 | 0.495 | 0.379 |
| **Sensitivity Index** | 0.336 | 0.592 | 0.442 | 0.348 | 0.330 | 0.545 | 0.497 | 0.551 | 0.359 | 0.707 | 0.643 | 0.441 | 0.434 |
| **Socioeconomic Structures Index** | 0.556 | 0.500 | 0.542 | 0.400 | 0.875 | 0.536 | 0.450 | 0.321 | 0.393 | 0.667 | 0.875 | 0.250 | 0.500 |
| **Institutions Services and Infrastructure for Adaptation Index** | 0.353 | 0.674 | 0.551 | 0.504 | 0.282 | 0.419 | 0.587 | 0.556 | 0.584 | 0.232 | 0.826 | 0.261 | 0.109 |
| **Sociopolitical Organization Index** | 0.667 | 0.875 | 0.833 | 0.450 | 0.750 | 0.679 | 0.450 | 0.679 | 0.643 | 0.583 | 0.750 | 0.750 | 0.667 |
| **Adaptive Capacity Index** | 0.500 | 0.702 | 0.649 | 0.406 | 0.641 | 0.525 | 0.462 | 0.491 | 0.518 | 0.460 | 0.873 | 0.366 | 0.372 |
| **Vulnerability Index** | 0.260 | 0.691 | 0.428 | 0.335 | 0.332 | 0.377 | 0.429 | 0.600 | 0.344 | 0.564 | 0.552 | 0.290 | 0.221 |
| **Temperature Index** | 0.302 | 0.786 | 0.547 | 0.800 | 0.215 | 0.530 | 0.571 | 0.796 | 0.755 | 0.619 | 0.500 | 0.929 | 0.286 |
| **Precipitation Index** | 0.524 | 0.643 | 0.524 | 0.657 | 0.571 | 0.653 | 0.457 | 0.735 | 0.551 | 0.429 | 0.643 | 0.714 | 0.619 |
| **Climate Scenario Index** | 0.445 | 0.769 | 0.577 | 0.785 | 0.423 | 0.637 | 0.554 | 0.824 | 0.703 | 0.564 | 0.615 | 0.885 | 0.487 |
| **Municipal Vulnerability Index** | 0.398 | 0.826 | 0.568 | 0.633 | 0.427 | 0.573 | 0.556 | 0.805 | 0.592 | 0.638 | 0.660 | 0.664 | 0.401 |
