# Supplementary material for: Mapping human vulnerability to climate change in the Brazilian Amazon: The construction of a municipal vulnerability index
Source: PLoS One. 2018 Feb 14;13(2):e0190808. doi: 10.1371/journal.pone.0190808 (PMC5812563; doi:10.1371/journal.pone.0190808)
Supplement: S2 Table — (DOCX) [file pone.0190808.s008.docx]

**S2 Table. Values of the main indices and sub-indices, by municipality, that composed the Municipal Vulnerability Index of the state of Amazonas, Brazil.**

| **Municipalities** | **Index of Natural Disasters** | **Vegetation Cover Index** | **Exposure Index** | **Diseases Associated to Climate Index** | **Poverty Index** | **Sociodemographic Sensitivity Index** | **Sensitivity Index** | **Socioeconomic Structures Index** | **Institutions Services and Infrastructure for Adaptation Index** | **Sociopolitical Organization Index** | **Adaptive Capacity Index** | **Vulnerability Index** | **Temperature Index** | **Precipitation Index** | **Climate Scenario Index** | **Municipal Vulnerability Index** |
| --- | --- | --- | --- | --- | --- | --- | --- | --- | --- | --- | --- | --- | --- | --- | --- | --- |
| Alvarães | 0.200 | 0.286 | 0.256 | 0.552 | 0.688 | 0.307 | 0.404 | 0.750 | 0.261 | 0.500 | 0.472 | 0.197 | 0.286 | 0.714 | 0.539 | 0.416 |
| Amaturá | 0.200 | 0.429 | 0.331 | 0.097 | 0.750 | 0.171 | 0.036 | 0.500 | 0.478 | 0.750 | 0.565 | 0.062 | 0.143 | 0.429 | 0.308 | 0.209 |
| Anamã | 0.699 | 0.286 | 0.518 | 0.400 | 0.688 | 0.557 | 0.473 | 0.500 | 0.391 | 1.000 | 0.634 | 0.530 | 0.571 | 0.429 | 0.538 | 0.604 |
| Anori | 0.400 | 0.286 | 0.361 | 0.352 | 0.625 | 0.432 | 0.308 | 0.500 | 0.739 | 0.750 | 0.676 | 0.341 | 0.571 | 0.429 | 0.538 | 0.497 |
| Apuí | 0.500 | 0.429 | 0.489 | 0.759 | 0.500 | 0.341 | 0.441 | 0.250 | 0.435 | 0.750 | 0.440 | 0.358 | 0.571 | 0.286 | 0.461 | 0.463 |
| Atalaia do Norte | 0.700 | 0.286 | 0.519 | 0.614 | 1.000 | 0.262 | 0.633 | 0.500 | 0.761 | 0.500 | 0.579 | 0.601 | 0.571 | 0.714 | 0.692 | 0.731 |
| Autazes | 0.500 | 0.571 | 0.564 | 0.655 | 0.500 | 0.739 | 0.646 | 0.500 | 0.565 | 0.750 | 0.602 | 0.656 | 0.714 | 0.857 | 0.846 | 0.849 |
| Barcelos | 0.300 | 0.143 | 0.233 | 0.786 | 0.813 | 0.000 | 0.441 | 1.000 | 0.739 | 0.750 | 0.889 | 0.488 | 0.571 | 0.571 | 0.615 | 0.624 |
| Barreirinha | 0.700 | 0.571 | 0.669 | 0.358 | 0.625 | 0.523 | 0.376 | 0.500 | 0.434 | 0.750 | 0.546 | 0.507 | 1.000 | 0.429 | 0.769 | 0.722 |
| Benjamin Constant | 1.000 | 0.286 | 0.677 | 0.710 | 0.625 | 0.523 | 0.621 | 0.500 | 0.652 | 0.750 | 0.639 | 0.740 | 0.429 | 0.714 | 0.616 | 0.767 |
| Beruri | 0.300 | 0.429 | 0.384 | 0.304 | 0.750 | 0.659 | 0.520 | 0.750 | 0.739 | 1.000 | 0.889 | 0.643 | 0.571 | 0.571 | 0.615 | 0.711 |
| Boa Vista do Ramos | 0.200 | 0.571 | 0.406 | 0.304 | 0.625 | 0.705 | 0.465 | 0.500 | 0.391 | 0.750 | 0.528 | 0.377 | 1.000 | 0.429 | 0.769 | 0.648 |
| Boca do Acre | 0.900 | 0.429 | 0.699 | 0.635 | 0.688 | 0.750 | 0.770 | 0.250 | 0.652 | 1.000 | 0.639 | 0.856 | 0.857 | 0.714 | 0.846 | 0.962 |
| Borba | 0.800 | 0.143 | 0.496 | 0.607 | 0.625 | 0.523 | 0.549 | 0.500 | 0.543 | 0.500 | 0.486 | 0.466 | 0.571 | 0.714 | 0.692 | 0.655 |
| Caapiranga | 0.699 | 0.286 | 0.518 | 0.752 | 0.625 | 0.478 | 0.619 | 0.500 | 0.413 | 0.750 | 0.537 | 0.563 | 0.571 | 0.429 | 0.538 | 0.623 |
| Canutama | 0.700 | 0.429 | 0.594 | 0.290 | 0.750 | 0.523 | 0.415 | 0.750 | 0.304 | 0.750 | 0.597 | 0.517 | 0.571 | 0.429 | 0.538 | 0.597 |
| Carauari | 0.400 | 0.000 | 0.211 | 0.407 | 0.750 | 0.443 | 0.441 | 0.500 | 0.565 | 0.250 | 0.389 | 0.136 | 0.429 | 0.714 | 0.616 | 0.425 |
| Careiro | 0.699 | 0.571 | 0.668 | 0.642 | 0.688 | 0.387 | 0.523 | 0.500 | 0.739 | 0.500 | 0.569 | 0.621 | 1.000 | 0.714 | 0.923 | 0.873 |
| Careiro da Várzea | 0.900 | 0.857 | 0.925 | 0.434 | 0.750 | 0.739 | 0.666 | 0.500 | 0.869 | 0.750 | 0.731 | 1.000 | 0.714 | 0.714 | 0.769 | 1.000 |
| Coari | 0.300 | 0.143 | 0.233 | 0.642 | 0.500 | 0.443 | 0.431 | 0.500 | 0.282 | 0.750 | 0.481 | 0.206 | 0.429 | 0.571 | 0.539 | 0.421 |
| Codajás | 0.400 | 0.143 | 0.286 | 0.448 | 0.563 | 0.387 | 0.301 | 0.500 | 0.739 | 0.750 | 0.676 | 0.285 | 0.571 | 0.714 | 0.692 | 0.552 |
| Eirunepé | 0.400 | 0.143 | 0.286 | 0.538 | 0.688 | 0.580 | 0.585 | 0.500 | 0.478 | 0.750 | 0.565 | 0.402 | 0.571 | 0.714 | 0.692 | 0.618 |
| Envira | 0.400 | 0.429 | 0.436 | 0.400 | 0.875 | 0.568 | 0.610 | 0.500 | 0.587 | 0.750 | 0.611 | 0.551 | 0.714 | 0.571 | 0.692 | 0.703 |
| Fonte Boa | 0.000 | 0.429 | 0.226 | 0.214 | 0.813 | 0.353 | 0.288 | 0.500 | 0.087 | 0.750 | 0.398 | 0.049 | 0.286 | 0.571 | 0.462 | 0.288 |
| Guajará | 0.600 | 0.429 | 0.542 | 0.586 | 0.750 | 0.432 | 0.558 | 0.500 | 0.304 | 0.750 | 0.491 | 0.507 | 0.571 | 1.000 | 0.846 | 0.765 |
| Humaitá | 0.700 | 0.429 | 0.594 | 0.793 | 0.375 | 0.432 | 0.441 | 0.250 | 0.826 | 0.250 | 0.394 | 0.397 | 0.571 | 0.571 | 0.615 | 0.572 |
| Ipixuna | 0.300 | 0.143 | 0.233 | 0.186 | 0.938 | 0.512 | 0.466 | 0.750 | 0.478 | 1.000 | 0.778 | 0.430 | 0.571 | 0.714 | 0.692 | 0.634 |
| Iranduba | 0.700 | 0.714 | 0.744 | 0.703 | 0.375 | 1.000 | 0.774 | 0.250 | 0.348 | 0.500 | 0.296 | 0.657 | 1.000 | 0.714 | 0.923 | 0.893 |
| Itacoatiara | 0.400 | 0.714 | 0.586 | 0.800 | 0.125 | 0.739 | 0.486 | 0.250 | 0.304 | 0.250 | 0.171 | 0.272 | 1.000 | 0.714 | 0.923 | 0.676 |
| Itamarati | 0.500 | 0.143 | 0.338 | 0.497 | 0.938 | 0.489 | 0.667 | 0.500 | 0.130 | 0.500 | 0.310 | 0.321 | 0.571 | 0.571 | 0.615 | 0.529 |
| Itapiranga | 0.300 | 0.571 | 0.458 | 0.441 | 0.375 | 0.614 | 0.323 | 0.500 | 0.804 | 0.750 | 0.704 | 0.435 | 0.571 | 0.571 | 0.615 | 0.594 |
| Japurá | 0.200 | 0.143 | 0.181 | 0.490 | 0.750 | 0.296 | 0.397 | 1.000 | 0.108 | 0.750 | 0.620 | 0.242 | 0.000 | 0.286 | 0.154 | 0.224 |
| Juruá | 0.200 | 0.286 | 0.256 | 0.186 | 1.000 | 0.478 | 0.486 | 0.500 | 0.391 | 0.750 | 0.528 | 0.290 | 0.286 | 0.286 | 0.308 | 0.338 |
| Jutaí | 0.100 | 0.143 | 0.128 | 0.503 | 0.750 | 0.273 | 0.390 | 0.750 | 0.282 | 0.750 | 0.588 | 0.179 | 0.286 | 0.571 | 0.462 | 0.362 |
| Lábrea | 0.900 | 0.429 | 0.699 | 0.931 | 0.813 | 0.659 | 1.000 | 0.750 | 0.391 | 0.500 | 0.528 | 0.936 | 0.714 | 0.429 | 0.615 | 0.877 |
| Manacapuru | 0.900 | 0.571 | 0.774 | 0.745 | 0.313 | 0.568 | 0.459 | 0.000 | 0.413 | 0.750 | 0.324 | 0.484 | 0.714 | 0.714 | 0.769 | 0.708 |
| Manaquiri | 0.600 | 0.429 | 0.542 | 0.448 | 0.625 | 0.648 | 0.525 | 0.500 | 0.609 | 0.750 | 0.621 | 0.572 | 0.571 | 0.714 | 0.692 | 0.715 |
| Manaus | 0.700 | 0.429 | 0.594 | 0.835 | 0.000 | 0.512 | 0.265 | 0.000 | 0.348 | 0.750 | 0.296 | 0.213 | 0.857 | 0.714 | 0.846 | 0.599 |
| Manicoré | 0.500 | 0.429 | 0.489 | 0.593 | 0.750 | 0.387 | 0.532 | 0.500 | 0.391 | 0.500 | 0.421 | 0.406 | 0.571 | 0.286 | 0.461 | 0.490 |
| Maraã | 0.600 | 0.429 | 0.542 | 0.331 | 0.750 | 0.262 | 0.262 | 0.750 | 0.456 | 0.750 | 0.662 | 0.422 | 0.429 | 0.857 | 0.693 | 0.630 |
| Maués | 0.200 | 0.286 | 0.256 | 0.703 | 0.438 | 0.523 | 0.486 | 0.750 | 0.565 | 0.500 | 0.602 | 0.340 | 0.571 | 0.571 | 0.615 | 0.540 |
| Nhamundá | 0.800 | 0.429 | 0.647 | 0.352 | 0.625 | 0.693 | 0.490 | 0.250 | 0.870 | 0.750 | 0.625 | 0.622 | 0.571 | 0.714 | 0.692 | 0.743 |
| Nova Olinda do Norte | 0.500 | 0.429 | 0.489 | 0.497 | 0.625 | 0.432 | 0.409 | 0.500 | 0.478 | 0.250 | 0.352 | 0.277 | 0.714 | 0.857 | 0.846 | 0.635 |
| Novo Airão | 0.100 | 0.143 | 0.128 | 0.648 | 0.563 | 0.603 | 0.590 | 0.500 | 0.913 | 0.750 | 0.750 | 0.424 | 0.857 | 0.571 | 0.769 | 0.674 |
| Novo Aripuanã | 0.699 | 0.286 | 0.518 | 0.676 | 0.688 | 0.353 | 0.523 | 0.750 | 0.739 | 0.250 | 0.569 | 0.520 | 0.571 | 0.429 | 0.538 | 0.599 |
| Parintins | 0.700 | 0.714 | 0.744 | 0.448 | 0.250 | 0.523 | 0.177 | 0.250 | 0.652 | 0.250 | 0.319 | 0.270 | 1.000 | 0.429 | 0.769 | 0.588 |
| Pauini | 0.699 | 0.143 | 0.443 | 0.331 | 0.875 | 0.353 | 0.413 | 0.750 | 0.696 | 0.750 | 0.764 | 0.526 | 0.714 | 0.571 | 0.692 | 0.689 |
| Presidente Figueiredo | 0.400 | 0.429 | 0.436 | 0.738 | 0.125 | 0.648 | 0.379 | 0.000 | 0.217 | 0.750 | 0.241 | 0.146 | 1.000 | 0.857 | 1.000 | 0.648 |
| Rio Preto da Eva | 0.500 | 0.429 | 0.489 | 0.910 | 0.438 | 0.341 | 0.503 | 0.500 | 0.304 | 0.750 | 0.491 | 0.434 | 0.857 | 0.571 | 0.769 | 0.680 |
| Santa Isabel do Rio Negro | 0.000 | 0.000 | 0.000 | 0.703 | 0.938 | 0.341 | 0.707 | 1.000 | 1.000 | 0.750 | 1.000 | 0.585 | 0.429 | 0.857 | 0.693 | 0.722 |
| Santo Antônio do Içá | 0.300 | 0.286 | 0.308 | 0.345 | 0.750 | 0.387 | 0.359 | 0.500 | 0.261 | 1.000 | 0.579 | 0.274 | 0.286 | 0.429 | 0.385 | 0.373 |
| São Gabriel da Cachoeira | 0.100 | 0.286 | 0.203 | 1.000 | 0.563 | 0.603 | 0.835 | 1.000 | 0.652 | 0.750 | 0.852 | 0.709 | 0.143 | 0.571 | 0.385 | 0.618 |
| São Paulo de Olivença | 0.800 | 0.429 | 0.647 | 0.255 | 0.688 | 0.250 | 0.158 | 0.750 | 0.348 | 0.750 | 0.616 | 0.392 | 0.429 | 0.571 | 0.539 | 0.526 |
| São Sebastião do Uatumã | 0.200 | 0.429 | 0.331 | 0.234 | 0.625 | 0.432 | 0.226 | 0.250 | 0.695 | 0.750 | 0.551 | 0.181 | 0.571 | 0.714 | 0.692 | 0.493 |
| Silves | 0.300 | 0.429 | 0.384 | 0.338 | 0.500 | 0.523 | 0.275 | 0.250 | 0.522 | 0.500 | 0.371 | 0.128 | 0.857 | 0.571 | 0.769 | 0.507 |
| Tabatinga | 0.400 | 0.286 | 0.361 | 0.635 | 0.500 | 0.603 | 0.537 | 0.250 | 0.152 | 0.000 | 0.000 | 0.039 | 0.286 | 0.714 | 0.539 | 0.327 |
| Tapauá | 0.300 | 0.143 | 0.233 | 0.600 | 0.938 | 0.443 | 0.706 | 0.500 | 0.000 | 0.500 | 0.255 | 0.239 | 0.571 | 0.429 | 0.538 | 0.440 |
| Tefé | 0.500 | 0.286 | 0.414 | 0.696 | 0.375 | 0.432 | 0.374 | 0.250 | 0.000 | 0.750 | 0.255 | 0.137 | 0.286 | 0.429 | 0.385 | 0.295 |
| Tonantins | 0.300 | 0.286 | 0.308 | 0.000 | 0.750 | 0.216 | 0.000 | 0.750 | 0.152 | 0.750 | 0.532 | 0.000 | 0.000 | 0.000 | 0.000 | 0.000 |
| Uarini | 0.500 | 0.286 | 0.414 | 0.448 | 0.875 | 0.398 | 0.525 | 0.500 | 0.065 | 0.750 | 0.389 | 0.329 | 0.286 | 0.714 | 0.539 | 0.491 |
| Urucará | 0.200 | 0.286 | 0.256 | 0.407 | 0.500 | 0.478 | 0.292 | 0.250 | 0.478 | 0.750 | 0.458 | 0.112 | 0.571 | 0.571 | 0.615 | 0.411 |
| Urucurituba | 0.900 | 1.000 | 1.000 | 0.152 | 0.563 | 0.603 | 0.245 | 0.500 | 0.413 | 0.500 | 0.431 | 0.564 | 0.857 | 0.571 | 0.769 | 0.754 |
| **AVERAGE VALUES OF THE INDICES** | **0.482** | **0.367** | **0.447** | **0.517** | **0.634** | **0.482** | **0.465** | **0.508** | **0.477** | **0.661** | **0.530** | **0.406** | **0.581** | **0.590** | **0.630** | **0.586** |
