# Supplementary material for: Mapping human vulnerability to climate change in the Brazilian Amazon: The construction of a municipal vulnerability index
Source: PLoS One. 2018 Feb 14;13(2):e0190808. doi: 10.1371/journal.pone.0190808 (PMC5812563; doi:10.1371/journal.pone.0190808)
Supplement: S3 Table — (DOCX) [file pone.0190808.s009.docx]

**S3 Table. Raw values of the variables used to compose the Exposure Index of the municipalities of the State of Amazonas, Brazil.**

| **Municipalities** | **Vegetation Cover Index** | | **Natural Disasters Susceptibility Index** | | **Natural Disasters Occurrence Index** | |
| --- | --- | --- | --- | --- | --- | --- |
|  | **Native vegetation cover (%)** | **Accumulated deforestation (%)** | **Consecutive dry days** | **Population at risk (%)** | **Disaster occurrence (%)** | **Deaths related to natural disasters (%)** |
| Alvarães | 90.32 | 1.27 | 12.63 | 1.90 | 1.22 | 0.00 |
| Amaturá | 74.37 | 0.22 | 13.27 | 1.03 | 1.22 | 0.00 |
| Anamã | 80.81 | 1.12 | 16.20 | 33.93 | 1.84 | 0.00 |
| Anori | 77.71 | 0.28 | 16.02 | 19.70 | 1.22 | 0.00 |
| Apuí | 94.64 | 2.02 | 44.20 | 2.69 | 0.41 | 0.00 |
| Atalaia do Norte | 72.73 | 0.06 | 15.93 | 2.60 | 1.63 | 25.00 |
| Autazes | 51.64 | 5.80 | 18.62 | 0.00 | 1.63 | 37.50 |
| Barcelos | 77.13 | 0.06 | 15.54 | 1.68 | 0.61 | 0.00 |
| Barreirinha | 47.76 | 2.61 | 23.80 | 1.84 | 2.24 | 0.00 |
| Benjamin Constant | 70.81 | 0.17 | 15.66 | 8.79 | 2.45 | 41.67 |
| Beruri | 74.16 | 0.23 | 17.20 | 5.37 | 1.02 | 0.00 |
| Boa Vista do Ramos | 36.10 | 2.16 | 20.10 | 0.20 | 1.02 | 0.00 |
| Boca do Acre | 86.94 | 5.01 | 35.87 | 12.34 | 2.24 | 0.00 |
| Borba | 80.82 | 0.19 | 23.21 | 0.95 | 2.24 | 9.09 |
| Caapiranga | 89.97 | 0.46 | 15.19 | 25.76 | 1.84 | 0.00 |
| Canutama | 91.93 | 2.06 | 30.00 | 56.06 | 1.43 | 0.00 |
| Carauari | 96.91 | 0.14 | 15.68 | 1.36 | 1.43 | 0.00 |
| Careiro | 56.30 | 3.28 | 17.70 | 0.75 | 2.04 | 40.00 |
| Careiro da Várzea | 25.89 | 18.10 | 18.40 | 7.72 | 3.47 | 5.88 |
| Coari | 85.75 | 0.13 | 15.71 | 5.41 | 1.22 | 0.00 |
| Codajás | 86.64 | 0.19 | 14.17 | 12.77 | 1.43 | 0.00 |
| Eirunepé | 95.25 | 0.63 | 22.19 | 0.18 | 1.63 | 0.00 |
| Envira | 95.12 | 2.00 | 25.75 | 3.34 | 1.22 | 0.00 |
| Fonte Boa | 52.49 | 0.24 | 10.81 | 0.75 | 1.22 | 0.00 |
| Guajará | 91.17 | 2.08 | 20.99 | 4.01 | 2.86 | 0.00 |
| Humaitá | 72.35 | 0.63 | 31.27 | 0.27 | 1.63 | 25.00 |
| Ipixuna | 97.08 | 0.65 | 21.03 | 3.45 | 1.22 | 0.00 |
| Iranduba | 58.03 | 10.03 | 16.94 | 3.89 | 2.24 | 9.09 |
| Itacoatiara | 46.23 | 6.16 | 17.75 | 0.40 | 2.04 | 0.00 |
| Itamarati | 94.36 | 0.13 | 20.00 | 15.10 | 1.43 | 0.00 |
| Itapiranga | 21.36 | 0.70 | 18.17 | 1.97 | 1.02 | 0.00 |
| Japurá | 68.07 | 0.03 | 10.78 | 1.40 | 1.43 | 0.00 |
| Juruá | 81.24 | 0.20 | 12.20 | 1.09 | 1.22 | 0.00 |
| Jutaí | 90.11 | 0.10 | 14.62 | 0.14 | 1.43 | 0.00 |
| Lábrea | 90.03 | 3.51 | 36.25 | 2.68 | 1.63 | 12.50 |
| Manacapuru | 53.05 | 3.64 | 16.70 | 1.43 | 3.47 | 11.76 |
| Manaquiri | 67.43 | 1.33 | 18.18 | 3.03 | 2.24 | 0.00 |
| Manaus | 65.72 | 1.81 | 16.30 | 1.72 | 1.43 | 42.86 |
| Manicoré | 72.06 | 1.74 | 27.27 | 0.62 | 1.84 | 0.00 |
| Maraã | 76.03 | 0.30 | 13.49 | 17.42 | 1.43 | 14.29 |
| Maués | 90.53 | 0.62 | 23.79 | 0.00 | 1.02 | 0.00 |
| Nhamundá | 43.32 | 0.53 | 27.28 | 12.70 | 2.45 | 0.00 |
| Nova Olinda do Norte | 55.31 | 1.43 | 19.23 | 0.63 | 2.24 | 0.00 |
| Novo Airão | 86.76 | 0.11 | 14.89 | 0.00 | 1.22 | 0.00 |
| Novo Aripuanã | 86.39 | 1.21 | 35.07 | 1.93 | 1.63 | 0.00 |
| Parintins | 19.01 | 5.67 | 29.20 | 2.09 | 3.06 | 0.00 |
| Pauini | 97.48 | 0.24 | 26.87 | 7.89 | 1.84 | 0.00 |
| Presidente Figueiredo | 59.03 | 1.02 | 16.77 | 9.78 | 0.41 | 0.00 |
| Rio Preto da Eva | 73.00 | 1.40 | 16.03 | 9.31 | 1.43 | 0.00 |
| Santa Isabel do Rio Negro | 84.82 | 0.05 | 12.38 | 0.00 | 0.41 | 0.00 |
| Santo Antônio do Içá | 90.64 | 0.24 | 12.60 | 0.27 | 1.84 | 0.00 |
| São Gabriel da Cachoeira | 55.33 | 0.15 | 10.51 | 2.74 | 0.61 | 0.00 |
| São Paulo de Olivença | 74.15 | 0.39 | 13.96 | 1.30 | 1.63 | 125.00 |
| São Sebastião do Uatumã | 69.19 | 0.64 | 19.95 | 0.41 | 1.22 | 0.00 |
| Silves | 46.35 | 1.75 | 17.81 | 1.41 | 1.22 | 0.00 |
| Tabatinga | 87.66 | 0.81 | 12.51 | 0.79 | 2.24 | 0.00 |
| Tapauá | 87.46 | 0.06 | 20.44 | 4.28 | 0.82 | 0.00 |
| Tefé | 90.23 | 0.51 | 14.81 | 1.75 | 1.63 | 0.00 |
| Tonantins | 88.77 | 0.28 | 11.53 | 0.41 | 1.84 | 0.00 |
| Uarini | 91.42 | 0.63 | 12.14 | 0.00 | 2.04 | 40.00 |
| Urucará | 75.83 | 0.17 | 21.16 | 0.23 | 1.02 | 0.00 |
| Urucurituba | 4.27 | 10.23 | 21.34 | 5.98 | 1.63 | 12.50 |
