# Supplementary material for: Mapping human vulnerability to climate change in the Brazilian Amazon: The construction of a municipal vulnerability index
Source: PLoS One. 2018 Feb 14;13(2):e0190808. doi: 10.1371/journal.pone.0190808 (PMC5812563; doi:10.1371/journal.pone.0190808)
Supplement: S4 Table — (DOCX) [file pone.0190808.s010.docx]

**S4 Table. Raw values of the variables used to compose the Sensitivity Index of the municipalities of the state of Amazonas, Brazil.**

| **Municipalities** | **Diseases Associated with Climate Index** | | | | | | | | | | | | **Poverty Index** | | | | | **Sociodemographic Sensitivity Index** | | | | | | | |
| --- | --- | --- | --- | --- | --- | --- | --- | --- | --- | --- | --- | --- | --- | --- | --- | --- | --- | --- | --- | --- | --- | --- | --- | --- | --- |
|  | **Dengue** | | | **American Cutaneous Leishmaniasis** | | | **Malaria** | | | **Accidents with poisonous animals** | | | **Probability of dying before the age of 5 (1,000 live births)** | **Probability of dying before age 40** | **Households with per capita income below the poverty line (%)** | **Households with inadequate sanitation (%)** | **Population aged over 25 that is illiterate (%)** | **Young householders (%)** | **Female householders with incomplete primary school or no education (%)** | **Population with disabilities (%)** | **Population that is 60 years old or older (%)** | **Population under 5 years old (%)** | **Riverine population (%)** | **Elderly aged 60 years old or older for 2040 (%)** | **Children aged 0 to 4 years old for 2040 (%)** |
|  | **Cases (%)** | **Incidence rate (100.000)** | **Tendency** | **Cases (%)** | **Incidence rate (100.000)** | **Tendency** | **Cases (%)** | **Incidence rate (100.000)** | **Tendency** | **Cases (%)** | **Incidence rate (100.000)** | **Tendency** |  |  |  |  |  |  |  |  |  |  |  |  |  |
| Alvarães | 0.03 | 19.61 | -21.79 | 0.14 | 21.71 | -32.63 | 1.91 | 170.69 | -12.62 | 1.51 | 166.34 | -11.35 | 22.28 | 4.83 | 74.57 | 35.55 | 26.88 | 4.65 | 3.65 | 15.07 | 4.7 | 18.13 | 26.09 | 11.88 | 5.98 |
| Amaturá | 0.00 | 0.00 | 0.00 | 0.03 | 6.53 | -10.77 | 0.06 | 8.04 | 0.25 | 0.28 | 46.10 | 14.60 | 23.22 | 5.03 | 80.61 | 32.45 | 29.7 | 2.86 | 2.21 | 18.04 | 5.14 | 15.66 | 36.84 | 11.20 | 6.30 |
| Anamã | 0.02 | 20.95 | -13.78 | 0.05 | 11.26 | 1.35 | 0.10 | 16.46 | 0.05 | 0.91 | 178.00 | -14.07 | 28.89 | 6.21 | 63.72 | 45.46 | 21.87 | 3.93 | 3.28 | 17.17 | 5.94 | 14.63 | 63.69 | 13.17 | 6.46 |
| Anori | 0.03 | 20.82 | -1.83 | 0.04 | 4.92 | -2.55 | 0.04 | 3.78 | -0.15 | 0.68 | 74.51 | 10.07 | 25.97 | 5.6 | 67.43 | 33.82 | 25.64 | 3.83 | 3.93 | 21 | 5.91 | 14.98 | 37.85 | 11.17 | 6.98 |
| Apuí | 0.03 | 15.59 | -17.33 | 1.42 | 168.89 | -28.86 | 0.45 | 31.00 | -3.36 | 2.26 | 192.13 | 52.54 | 23.41 | 5.07 | 51.23 | 42.01 | 14.39 | 5.82 | 4.32 | 18.65 | 5.23 | 11.37 | 2.85 | 13.61 | 5.97 |
| Atalaia do Norte | 0.00 | 2.39 | 2.51 | 0.35 | 58.84 | -25.52 | 2.86 | 248.75 | -59.76 | 1.10 | 129.79 | 59.70 | 30.62 | 6.57 | 76.97 | 61.98 | 43.57 | 4.5 | 4.11 | 13.35 | 4.04 | 16.81 | 43.46 | 10.53 | 6.62 |
| Autazes | 0.05 | 15.08 | -7.60 | 0.54 | 38.61 | -0.69 | 1.98 | 83.47 | -2.19 | 2.35 | 121.56 | -8.22 | 19.07 | 4.15 | 66.66 | 45.37 | 20.31 | 5.07 | 4.71 | 23.06 | 7.06 | 15.53 | 24.65 | 18.19 | 5.55 |
| Barcelos | 0.65 | 243.62 | -400.72 | 1.03 | 80.83 | -20.80 | 3.18 | 144.21 | 26.04 | 1.09 | 61.43 | 16.55 | 31.5 | 6.75 | 70.63 | 46.64 | 37.63 | 3.63 | 3.12 | 13.79 | 5.51 | 12.05 | 33.49 | 15.43 | 4.94 |
| Barreirinha | 0.01 | 2.12 | -1.30 | 0.17 | 12.93 | 3.38 | 0.00 | 0.14 | 0.00 | 1.47 | 83.01 | 24.27 | 22.97 | 4.97 | 75.24 | 44.62 | 14.2 | 4.41 | 4.24 | 20.91 | 6.57 | 17.6 | 20.90 | 15.02 | 6.01 |
| Benjamin Constant | 0.02 | 5.15 | 4.39 | 0.23 | 18.37 | 2.77 | 0.46 | 16.99 | -14.07 | 2.41 | 128.25 | 17.12 | 24.87 | 5.37 | 69.07 | 37.11 | 29.88 | 4.91 | 5.54 | 18.94 | 5.65 | 17.67 | 8.72 | 12.15 | 6.52 |
| Beruri | 0.00 | 2.86 | -4.94 | 0.05 | 7.40 | -1.54 | 0.45 | 40.88 | -3.57 | 0.75 | 79.49 | 16.19 | 30.96 | 6.64 | 70.31 | 37.6 | 33.1 | 5.35 | 5.28 | 20.76 | 5.49 | 18.18 | 42.79 | 14.92 | 6.09 |
| Boa Vista do Ramos | 0.01 | 7.12 | -2.55 | 0.07 | 11.92 | 3.41 | 0.01 | 1.24 | -0.04 | 0.98 | 117.19 | -23.24 | 27.26 | 5.87 | 75.88 | 42.26 | 10.36 | 5.71 | 6.19 | 24.77 | 5.4 | 15.72 | 45.72 | 13.91 | 5.95 |
| Boca do Acre | 0.23 | 71.08 | -65.46 | 2.48 | 178.67 | 15.67 | 0.82 | 35.58 | -0.51 | 1.03 | 51.71 | 6.48 | 22.57 | 4.89 | 63.93 | 36.77 | 35.01 | 5.33 | 6.09 | 22.31 | 6.92 | 14.23 | 72.03 | 17.09 | 5.49 |
| Borba | 0.44 | 125.77 | -66.55 | 0.42 | 26.48 | -8.45 | 2.60 | 96.13 | -13.70 | 2.48 | 113.21 | -0.94 | 22.73 | 4.92 | 69.87 | 44.16 | 20.6 | 4.61 | 4.32 | 18.52 | 5.9 | 16.75 | 33.04 | 15.76 | 5.37 |
| Caapiranga | 0.03 | 36.34 | 0.00 | 0.17 | 33.75 | 18.97 | 0.71 | 85.43 | -9.66 | 0.81 | 119.94 | 42.26 | 22.85 | 4.95 | 65.69 | 34.23 | 19.59 | 4.63 | 3.06 | 20.18 | 5.54 | 14.93 | 46.72 | 14.37 | 6.01 |
| Canutama | 0.01 | 5.86 | -1.96 | 0.21 | 37.75 | -11.01 | 1.38 | 156.17 | -9.07 | 0.43 | 56.08 | -0.27 | 26.92 | 5.8 | 70.81 | 51.57 | 36.22 | 4.36 | 4.37 | 21.44 | 6.6 | 12.64 | 90.70 | 22.26 | 4.33 |
| Carauari | 0.00 | 1.66 | -0.70 | 0.88 | 72.66 | -30.37 | 0.65 | 31.21 | -17.05 | 1.32 | 79.21 | 18.86 | 28.23 | 6.08 | 68.9 | 21.6 | 39.7 | 5.07 | 4.99 | 24.01 | 5.41 | 16.16 | 21.74 | 15.64 | 5.65 |
| Careiro | 0.31 | 91.90 | -118.76 | 0.30 | 21.78 | -0.85 | 4.41 | 204.73 | 1.82 | 1.46 | 78.36 | -5.45 | 22.1 | 4.79 | 69.78 | 55.58 | 17.76 | 5 | 4.14 | 13.94 | 5.97 | 13.85 | 31.32 | 15.89 | 5.68 |
| Careiro da Várzea | 0.05 | 23.59 | 2.46 | 0.04 | 4.26 | -0.60 | 0.41 | 26.45 | 1.70 | 0.47 | 36.24 | 14.39 | 22.1 | 4.79 | 67.86 | 79.98 | 21.41 | 4.58 | 4.74 | 24.67 | 7.8 | 12.24 | 90.22 | 16.59 | 6.28 |
| Coari | 1.49 | 197.31 | -324.31 | 1.79 | 51.64 | -46.78 | 4.04 | 69.54 | -4.28 | 4.12 | 85.24 | -12.91 | 20.96 | 4.82 | 57.19 | 34.8 | 23.98 | 5.66 | 5.2 | 17.37 | 5.26 | 14.28 | 31.01 | 13.64 | 5.46 |
| Codajás | 0.41 | 178.14 | -283.55 | 0.16 | 18.29 | -3.08 | 0.22 | 14.88 | -3.39 | 1.19 | 93.60 | -8.73 | 22.85 | 4.95 | 70.57 | 31.65 | 24.78 | 4.77 | 3.97 | 16.8 | 5.59 | 13.82 | 28.49 | 11.94 | 6.49 |
| Eirunepé | 0.01 | 4.65 | -2.79 | 1.01 | 72.20 | -0.58 | 2.75 | 108.74 | -45.31 | 1.32 | 67.50 | -14.00 | 26.1 | 5.63 | 66.4 | 39.94 | 43.35 | 5.07 | 6.21 | 18.25 | 5.31 | 16.53 | 97.46 | 15.55 | 5.64 |
| Envira | 0.00 | 2.36 | -2.89 | 0.88 | 113.34 | -10.15 | 0.09 | 6.72 | 2.26 | 1.01 | 95.89 | -1.80 | 31.7 | 6.79 | 73.6 | 34.11 | 42.35 | 4.76 | 4.98 | 18.58 | 4.92 | 16.24 | 95.80 | 12.53 | 5.93 |
| Fonte Boa | 0.00 | 1.76 | -1.18 | 0.06 | 4.76 | -4.58 | 0.13 | 6.89 | -4.22 | 0.97 | 58.53 | 17.22 | 33.44 | 7.15 | 69.22 | 31 | 31.41 | 3.46 | 4.57 | 18.29 | 5.7 | 17.68 | 33.60 | 12.43 | 5.82 |
| Guajará | 0.01 | 4.11 | -4.46 | 0.28 | 43.09 | -4.29 | 2.37 | 222.04 | 0.67 | 0.54 | 60.79 | -7.57 | 25.1 | 5.42 | 75.36 | 47.71 | 43.16 | 4.63 | 4.3 | 15.39 | 4.55 | 16.07 | 91.97 | 12.49 | 6.02 |
| Humaitá | 1.15 | 260.16 | -507.76 | 2.19 | 121.63 | 12.19 | 1.96 | 69.53 | 5.19 | 2.57 | 108.80 | -13.93 | 20.2 | 4.39 | 54.77 | 25.96 | 22.82 | 4.29 | 4.14 | 19.74 | 6.22 | 12.71 | 31.23 | 13.88 | 6.05 |
| Ipixuna | 0.00 | 0.73 | -0.79 | 0.53 | 59.58 | -7.83 | 1.87 | 111.89 | -17.78 | 0.44 | 34.30 | -17.06 | 23.33 | 5.05 | 83.67 | 58.72 | 46.58 | 3.79 | 2.3 | 10.27 | 4.49 | 19.09 | 93.76 | 13.13 | 6.50 |
| Iranduba | 0.14 | 34.98 | -24.30 | 0.28 | 15.80 | 0.51 | 2.69 | 86.08 | -2.01 | 2.49 | 101.18 | 35.93 | 19.01 | 4.14 | 55.58 | 16.04 | 17.41 | 5.88 | 6.59 | 24.8 | 6.98 | 14.39 | 40.29 | 18.00 | 5.79 |
| Itacoatiara | 1.81 | 208.02 | -120.11 | 5.45 | 141.79 | 1.22 | 2.11 | 31.69 | -0.77 | 5.15 | 94.59 | 0.75 | 16.39 | 3.81 | 51.1 | 24.2 | 11.96 | 5.7 | 5.6 | 22.68 | 7.32 | 13.51 | 18.58 | 17.91 | 5.74 |
| Itamarati | 0.00 | 4.16 | 2.77 | 0.10 | 26.56 | -1.78 | 0.36 | 56.14 | 28.52 | 0.34 | 63.57 | 18.46 | 23.33 | 5.05 | 87.31 | 54.59 | 48.14 | 5.92 | 5.73 | 9.78 | 4.36 | 16.94 | 97.60 | 12.31 | 5.78 |
| Itapiranga | 0.00 | 4.26 | 0.00 | 0.43 | 112.18 | 5.61 | 0.33 | 48.19 | 0.28 | 0.49 | 87.60 | 9.07 | 20.03 | 4.35 | 64.36 | 22.15 | 11.35 | 4.27 | 4.05 | 25.73 | 7.39 | 14.29 | 14.76 | 16.43 | 5.60 |
| Japurá | 0.02 | 27.60 | -15.11 | 0.10 | 34.05 | 6.29 | 0.40 | 77.18 | -13.91 | 0.47 | 93.44 | -2.13 | 27.72 | 5.97 | 71.07 | 45.45 | 32.48 | 4.58 | 3.96 | 10.88 | 3.71 | 16.61 | 37.82 | 12.96 | 5.82 |
| Juruá | 0.01 | 8.72 | -4.87 | 0.08 | 19.57 | -8.49 | 0.42 | 53.76 | -28.95 | 0.30 | 52.58 | -14.69 | 36.64 | 7.81 | 68.13 | 42.49 | 44.08 | 4.97 | 5.74 | 20.22 | 4.51 | 16.21 | 42.04 | 10.85 | 7.16 |
| Jutaí | 0.02 | 9.15 | -8.47 | 0.04 | 3.48 | -1.48 | 1.40 | 86.09 | 17.88 | 0.86 | 66.58 | 26.37 | 24.44 | 5.28 | 76.69 | 36.84 | 36.21 | 4.92 | 4.18 | 17.27 | 4.96 | 20.66 | 38.76 | 11.03 | 6.04 |
| Lábrea | 0.82 | 205.29 | -356.87 | 1.75 | 111.32 | 8.41 | 3.69 | 140.53 | 38.86 | 1.75 | 78.23 | 9.91 | 28.45 | 6.12 | 65.65 | 41.29 | 38.06 | 5.28 | 5.54 | 25.82 | 5.99 | 15.09 | 36.51 | 14.42 | 5.83 |
| Manacapuru | 1.52 | 172.07 | -104.78 | 1.13 | 28.67 | -2.87 | 2.78 | 41.78 | -1.11 | 4.42 | 79.82 | 10.76 | 18.45 | 4.33 | 52.54 | 23.74 | 21.21 | 4.47 | 5.3 | 24.1 | 6.89 | 13.55 | 24.44 | 15.74 | 5.86 |
| Manaquiri | 0.04 | 17.80 | -10.35 | 0.04 | 4.58 | -2.97 | 0.59 | 49.10 | -2.14 | 0.73 | 63.21 | -2.57 | 27.62 | 5.95 | 67.4 | 62.9 | 14.12 | 4.5 | 2.77 | 18.4 | 6.5 | 15.34 | 54.40 | 11.74 | 7.08 |
| Manaus | 84.34 | 472.40 | -234.07 | 40.00 | 52.23 | -2.34 | 20.86 | 15.56 | -1.29 | 9.00 | 8.10 | 2.74 | 15.2 | 3.32 | 32.65 | 0.94 | 4.76 | 5.75 | 4.83 | 25.61 | 6.04 | 10.88 | 0.15 | 14.85 | 6.61 |
| Manicoré | 0.30 | 61.03 | -47.95 | 0.92 | 44.48 | -16.33 | 2.53 | 74.88 | -1.86 | 1.89 | 67.32 | -17.81 | 27.77 | 5.98 | 60.73 | 49.63 | 22.42 | 4.05 | 4.17 | 15.16 | 6.3 | 13.89 | 33.05 | 14.26 | 5.87 |
| Maraã | 0.03 | 16.61 | -18.19 | 0.10 | 11.62 | -15.14 | 0.43 | 29.22 | 0.73 | 0.62 | 52.76 | -31.53 | 24.87 | 5.37 | 80.26 | 47.76 | 24.01 | 4.71 | 3.31 | 15.37 | 3.64 | 17.74 | 49.13 | 11.65 | 5.76 |
| Maués | 0.24 | 44.96 | -97.69 | 1.41 | 61.78 | 12.38 | 0.42 | 10.22 | 8.44 | 3.28 | 105.52 | 27.89 | 18.93 | 4.12 | 68.25 | 47.17 | 13.83 | 4.45 | 5.43 | 19.51 | 6.5 | 16.51 | 19.68 | 14.65 | 5.81 |
| Nhamundá | 0.01 | 6.36 | -1.95 | 0.44 | 53.61 | -10.19 | 0.16 | 11.05 | -0.13 | 0.75 | 65.26 | 6.67 | 22.16 | 4.8 | 72.84 | 53.88 | 12.36 | 4.22 | 3.29 | 19.34 | 7.59 | 13.89 | 51.77 | 18.23 | 5.45 |
| Nova Olinda do Norte | 0.50 | 160.13 | -254.91 | 0.31 | 22.26 | -3.04 | 0.18 | 7.44 | 0.28 | 1.33 | 66.92 | 0.07 | 21.96 | 4.76 | 67.73 | 48.62 | 17.44 | 3.23 | 4.08 | 20.78 | 6.12 | 14.14 | 34.19 | 13.97 | 5.97 |
| Novo Airão | 0.14 | 103.16 | -110.54 | 0.50 | 92.32 | 3.14 | 0.87 | 127.11 | -8.76 | 1.23 | 171.52 | -10.70 | 22.68 | 4.91 | 62.58 | 32.23 | 19.97 | 4.64 | 4.46 | 17.43 | 6.44 | 14.67 | 35.22 | 13.74 | 6.52 |
| Novo Aripuanã | 0.50 | 220.01 | -419.62 | 0.92 | 94.78 | 48.47 | 1.36 | 86.36 | -5.00 | 0.64 | 50.36 | -11.02 | 25.66 | 5.54 | 65.6 | 61.01 | 25.35 | 4.27 | 4.63 | 21.36 | 6.52 | 13.57 | 25.45 | 16.37 | 5.07 |
| Parintins | 0.45 | 41.21 | -23.93 | 0.53 | 10.99 | 1.90 | 0.05 | 0.57 | -0.03 | 4.64 | 67.49 | -17.27 | 18.93 | 4.12 | 57.97 | 26.37 | 8.08 | 4.07 | 4.23 | 19.73 | 7.13 | 13.7 | 84.17 | 17.26 | 5.57 |
| Pauini | 0.00 | 0.00 | 0.00 | 0.44 | 52.31 | -10.74 | 0.64 | 43.54 | 28.64 | 0.47 | 39.73 | -12.73 | 32.32 | 6.92 | 74.86 | 47.13 | 36.62 | 3.87 | 4.25 | 16.99 | 4.58 | 17.02 | 92.13 | 11.94 | 5.84 |
| Presidente Figueiredo | 0.11 | 41.55 | -46.85 | 9.46 | 839.55 | -94.22 | 2.62 | 134.61 | -2.45 | 1.73 | 109.45 | -15.29 | 18.6 | 4.05 | 42.54 | 30.28 | 9.47 | 6.22 | 4.37 | 22.34 | 5.23 | 13.38 | 4.89 | 14.02 | 6.87 |
| Rio Preto da Eva | 0.14 | 59.61 | -8.41 | 14.75 | 1327.69 | -117.86 | 2.68 | 135.28 | -17.26 | 3.30 | 202.64 | 94.63 | 21.13 | 4.58 | 53.2 | 46.59 | 15.03 | 4.92 | 3.81 | 18.11 | 5.36 | 12.84 | 4.73 | 15.57 | 6.17 |
| Santa Isabel do Rio Negro | 0.01 | 13.40 | 0.00 | 0.58 | 93.00 | -47.83 | 1.11 | 101.87 | -4.19 | 1.00 | 114.74 | 29.10 | 29.81 | 6.4 | 78.76 | 53.57 | 33.33 | 3.06 | 2.97 | 12.34 | 5.74 | 19.76 | 18.61 | 10.26 | 7.38 |
| Santo Antônio do Içá | 0.00 | 0.85 | -0.37 | 0.08 | 6.16 | 6.35 | 0.84 | 38.96 | 23.37 | 0.92 | 47.57 | 2.88 | 25.65 | 5.54 | 82.75 | 41.68 | 28.55 | 3.74 | 2.54 | 17.89 | 4.73 | 17.31 | 34.83 | 12.78 | 6.00 |
| São Gabriel da Cachoeira | 1.49 | 372.75 | 302.97 | 0.79 | 45.01 | -10.85 | 3.75 | 121.38 | -3.52 | 4.96 | 209.74 | 5.60 | 20.7 | 4.98 | 68.9 | 47.5 | 19.14 | 5.01 | 4.14 | 18.55 | 6.79 | 15.56 | 18.00 | 16.03 | 5.99 |
| São Paulo de Olivença | 0.00 | 0.86 | 3.01 | 0.05 | 3.41 | -0.49 | 1.35 | 51.65 | -29.66 | 1.87 | 93.37 | 13.61 | 22.07 | 4.78 | 79.71 | 62.28 | 30.53 | 2.49 | 2.22 | 15.19 | 6.14 | 16.31 | 44.20 | 16.42 | 5.65 |
| São Sebastião do Uatumã | 0.00 | 1.11 | 0.00 | 0.15 | 33.58 | -5.12 | 0.58 | 82.10 | -1.80 | 0.21 | 35.14 | 5.41 | 29.35 | 6.31 | 65.21 | 31.47 | 13.95 | 4.31 | 3.09 | 20.12 | 5.24 | 15.78 | 35.80 | 11.12 | 6.67 |
| Silves | 0.00 | 4.93 | -11.50 | 0.36 | 89.68 | -30.17 | 0.44 | 62.58 | 0.52 | 0.44 | 78.69 | -15.22 | 20.2 | 4.39 | 63.94 | 47.98 | 10.53 | 4.16 | 4.11 | 26.83 | 6.28 | 15.65 | 19.84 | 14.97 | 5.82 |
| Tabatinga | 0.54 | 95.82 | 343.15 | 0.65 | 31.96 | 9.33 | 2.09 | 53.05 | -19.53 | 1.51 | 49.98 | -11.82 | 23.88 | 5.17 | 59.47 | 20.5 | 19.33 | 4.33 | 5.56 | 18.52 | 5.28 | 16.3 | 19.48 | 13.55 | 6.47 |
| Tapauá | 0.01 | 3.16 | -2.04 | 0.49 | 54.33 | -10.46 | 1.73 | 115.25 | 18.78 | 0.92 | 75.71 | 31.11 | 39.06 | 8.3 | 70.72 | 38.44 | 40.29 | 5.64 | 4.36 | 25.95 | 4.85 | 15.31 | 39.18 | 17.88 | 5.29 |
| Tefé | 1.80 | 280.60 | -372.11 | 1.74 | 58.72 | -84.37 | 3.12 | 60.33 | -2.80 | 2.37 | 56.81 | 20.90 | 18.74 | 4.08 | 52.37 | 14.6 | 19.58 | 4.8 | 4.68 | 21.52 | 5.72 | 15.2 | 20.38 | 13.72 | 5.75 |
| Tonantins | 0.00 | 0.00 | 0.00 | 0.02 | 2.63 | -4.05 | 0.06 | 3.99 | -0.15 | 0.65 | 54.24 | -3.97 | 22.14 | 4.8 | 72.92 | 62.29 | 26.79 | 3.29 | 3.39 | 14.27 | 4.8 | 17.62 | 32.94 | 11.98 | 5.95 |
| Uarini | 0.00 | 2.13 | -1.50 | 0.14 | 25.73 | -36.14 | 1.15 | 124.80 | 11.44 | 1.84 | 242.93 | -57.25 | 32.38 | 6.94 | 71.22 | 38.78 | 25.15 | 6.01 | 4.69 | 13.5 | 4.72 | 18.1 | 37.94 | 13.32 | 5.48 |
| Urucará | 0.00 | 2.22 | -1.72 | 0.20 | 22.46 | -10.09 | 0.27 | 17.18 | -0.10 | 0.78 | 65.89 | 16.75 | 26.44 | 5.7 | 68 | 20.38 | 10.69 | 3.49 | 3.43 | 22.76 | 6.98 | 15.2 | 29.88 | 15.26 | 5.82 |
| Urucurituba | 0.01 | 8.86 | -4.45 | 0.07 | 11.66 | -1.47 | 0.00 | 0.43 | 0.01 | 0.74 | 91.73 | -13.81 | 22.73 | 4.92 | 66.8 | 34.36 | 12.6 | 3.53 | 3.56 | 22.57 | 6.85 | 14.64 | 43.47 | 13.51 | 6.50 |
