# Supplementary material for: Mapping human vulnerability to climate change in the Brazilian Amazon: The construction of a municipal vulnerability index
Source: PLoS One. 2018 Feb 14;13(2):e0190808. doi: 10.1371/journal.pone.0190808 (PMC5812563; doi:10.1371/journal.pone.0190808)
Supplement: S5 Table — (DOCX) [file pone.0190808.s011.docx]

**S5 Table. Raw values and information on the variables that composed the Adaptive Capacity Index of the municipalities of the state of Amazonas, Brazil.**

| **Municipalities** | **Socioeconomic Structures Index** | **Institutions, Services, and Infrastructure for Adaptation Index** | | | | | | | **Sociopolitical Organization Index** |
| --- | --- | --- | --- | --- | --- | --- | --- | --- | --- |
|  | **Reverse FIRJAN Index** | **Health care services** | | **Risk Management Instruments for Landslides and Floods** | | **Security Institutions** | | |  |
|  |  | **Primary care coverage (%)** | **Hospital beds (per 1,000 inhabitants)** | **Landslides (nº)** | **Floods (nº)** | **Fire department** | **Civil Defense** | **Municipal guard** | **Municipal councils and consortia related to adaptation to climate (nº)** |
| Alvarães | 0.42 | 76.56 | 1.80 | 3 | 5 | No | Municipal coordination | Yes | 2 |
| Amaturá | 0.44 | 81.78 | 1.84 | 0 | 5 | No | Municipal coordination | No | 1 |
| Anamã | 0.46 | 100 | 1.87 | 0 | 3 | No | Municipal coordination | No | 0 |
| Anori | 0.46 | 100 | 1.19 | 0 | 0 | No | Municipal coordination | No | 1 |
| Apuí | 0.53 | 100 | 2.42 | 0 | 0 | No | Municipal coordination | No | 1 |
| Atalaia do Norte | 0.45 | 56.52 | 1.93 | 0 | 1 | No | Municipal coordination | No | 2 |
| Autazes | 0.42 | 90.06 | 1.67 | 0 | 0 | No | Unit that is not a municipal coordination | Yes | 1 |
| Barcelos | 0.34 | 53.99 | 0.98 | 0 | 3 | No | Unit that is not a municipal coordination | No | 1 |
| Barreirinha | 0.43 | 42.74 | 1.04 | 6 | 6 | No | Municipal coordination | Yes | 1 |
| Benjamin Constant | 0.43 | 54.33 | 1.06 | 0 | 6 | No | Municipal coordination | No | 1 |
| Beruri | 0.38 | 74.27 | 0.83 | 0 | 0 | No | Municipal coordination | Yes | 0 |
| Boa Vista do Ramos | 0.46 | 100 | 1.30 | 0 | 3 | No | Unit that is not a municipal coordination | No | 1 |
| Boca do Acre | 0.51 | 51.3 | 2.21 | 0 | 0 | No | Municipal coordination | Yes | 0 |
| Borba | 0.46 | 50.11 | 1.35 | 1 | 5 | No | Municipal coordination | Yes | 2 |
| Caapiranga | 0.45 | 100 | 1.77 | 0 | 1 | No | Municipal coordination | Yes | 1 |
| Canutama | 0.40 | 85.8 | 1.45 | 0 | 3 | No | Municipal coordination | Yes | 1 |
| Carauari | 0.47 | 34.44 | 1.79 | 0 | 4 | No | Municipal coordination | Yes | 4 |
| Careiro | 0.48 | 80.56 | 0.71 | 0 | 3 | No | Municipal coordination | No | 2 |
| Careiro da Várzea | 0.49 | 84.21 | 0.00 | 0 | 0 | No | Municipal coordination | Yes | 1 |
| Coari | 0.49 | 100 | 1.87 | 0 | 2 | No | Municipal coordination | Yes | 1 |
| Codajás | 0.44 | 74.79 | 2.39 | 0 | 0 | No | Municipal coordination | No | 1 |
| Eirunepé | 0.46 | 86.09 | 2.53 | 0 | 0 | No | Unit that is not a municipal coordination | Yes | 1 |
| Envira | 0.47 | 100 | 1.28 | 0 | 1 | No | Not available | Yes | 1 |
| Fonte Boa | 0.49 | 64.66 | 3.76 | 4 | 4 | No | Municipal coordination | Yes | 1 |
| Guajará | 0.46 | 83.36 | 1.64 | 0 | 4 | No | Municipal coordination | Yes | 1 |
| Humaitá | 0.51 | 52.88 | 0.82 | 0 | 0 | No | Municipal coordination | Yes | 3 |
| Ipixuna | 0.38 | 51.15 | 1.30 | 0 | 3 | No | Municipal coordination | Yes | 0 |
| Iranduba | 0.53 | 100 | 0.96 | 3 | 5 | No | Municipal coordination | No | 2 |
| Itacoatiara | 0.53 | 94.04 | 1.33 | 0 | 0 | Yes | Municipal coordination | Yes | 3 |
| Itamarati | 0.43 | 100 | 2.08 | 2 | 2 | No | Municipal coordination | Yes | 2 |
| Itapiranga | 0.50 | 100 | 2.79 | 0 | 1 | No | Not available | No | 1 |
| Japurá | ND | 100 | 4.29 | 1 | 1 | No | Municipal coordination | Yes | 1 |
| Juruá | 0.45 | 100 | 1.67 | 2 | 2 | No | Not available | Yes | 1 |
| Jutaí | 0.41 | 98.4 | 1.93 | 0 | 2 | No | Unit that is not a municipal coordination | Yes | 1 |
| Lábrea | 0.42 | 66.65 | 1.27 | 0 | 3 | Yes | Municipal coordination | No | 2 |
| Manacapuru | 0.60 | 84.74 | 0.86 | 2 | 0 | Yes | Municipal coordination | Yes | 1 |
| Manaquiri | 0.50 | 78.07 | 0.70 | 4 | 5 | No | Unit that is not a municipal coordination | No | 1 |
| Manaus | 0.70 | 52.34 | 2.34 | 2 | 1 | Yes | Unit that is not a municipal coordination | Yes | 1 |
| Manicoré | 0.50 | 62.02 | 1.47 | 2 | 2 | No | Unit that is not a municipal coordination | Yes | 2 |
| Maraã | 0.39 | 68.2 | 1.25 | 3 | 1 | No | Municipal coordination | Yes | 1 |
| Maués | 0.42 | 71.17 | 1.92 | 0 | 5 | No | Municipal coordination | No | 2 |
| Nhamundá | 0.51 | 32.05 | 2.06 | 2 | 1 | No | Unit that is not a municipal coordination | No | 1 |
| Nova Olinda do Norte | 0.45 | 56.69 | 1.02 | 0 | 4 | No | Municipal coordination | Yes | 5 |
| Novo Airão | 0.47 | 77.47 | 1.13 | 0 | 0 | No | Municipal coordination | No | 1 |
| Novo Aripuanã | 0.42 | 54.28 | 1.93 | 0 | 1 | No | Not available | No | 3 |
| Parintins | 0.60 | 77.35 | 1.77 | 1 | 1 | Yes | Municipal coordination | No | 3 |
| Pauini | 0.42 | 61.38 | 1.81 | 0 | 0 | No | Municipal coordination | No | 1 |
| Presidente Figueiredo | 0.70 | 100 | 1.62 | 0 | 1 | No | Municipal coordination | Yes | 1 |
| Rio Preto da Eva | 0.49 | 100 | 1.47 | 0 | 0 | Yes | Municipal coordination | Yes | 1 |
| Santa Isabel do Rio Negro | 0.35 | 49.3 | 0.89 | 0 | 0 | No | Not available | Yes | 1 |
| Santo Antônio do Içá | 0.45 | 84.37 | 2.32 | 4 | 4 | No | Unit that is not a municipal coordination | No | 0 |
| São Gabriel da Cachoeira | 0.37 | 58.85 | 4.43 | 0 | 0 | No | Not available | Yes | 1 |
| São Paulo de Olivença | 0.42 | 91.81 | 0.74 | 4 | 4 | No | Municipal coordination | No | 1 |
| São Sebastião do Uatumã | 0.51 | 100 | 1.64 | 0 | 0 | No | Not available | No | 1 |
| Silves | 0.51 | 100 | 4.51 | 0 | 0 | No | Not available | No | 2 |
| Tabatinga | 0.53 | 71.8 | 1.43 | 2 | 2 | Yes | Unit that is not a municipal coordination | Yes | 8 |
| Tapauá | 0.47 | 100 | 3.53 | 3 | 4 | No | Municipal coordination | Yes | 2 |
| Tefé | 0.53 | 65.16 | 2.10 | 3 | 3 | Yes | Municipal coordination | Yes | 1 |
| Tonantins | 0.39 | 100 | 1.95 | 2 | 4 | No | Municipal coordination | Yes | 1 |
| Uarini | 0.43 | 100 | 2.13 | 2 | 2 | No | Municipal coordination | Yes | 1 |
| Urucará | 0.58 | 100 | 2.68 | 0 | 4 | No | Not available | No | 1 |
| Urucurituba | 0.45 | 100 | 1.84 | 0 | 1 | No | Municipal coordination | Yes | 2 |
