# Supplementary material for: Mapping human vulnerability to climate change in the Brazilian Amazon: The construction of a municipal vulnerability index
Source: PLoS One. 2018 Feb 14;13(2):e0190808. doi: 10.1371/journal.pone.0190808 (PMC5812563; doi:10.1371/journal.pone.0190808)
Supplement: S6 Table — (DOCX) [file pone.0190808.s012.docx]

**S6 Table. Raw values of the climatic parameters used to compose the Climatic Scenario Index of the municipalities of the state of Amazonas, Brazil.**

| **Municipalities** | **CDD (days)** | **PRECPTOT (mm)** | **R95p (mm)** | **RX5day (mm)** | **Tmax (°C)** | **Tmin (°C)** |
| --- | --- | --- | --- | --- | --- | --- |
| Alvarães | -2.23 | 3.85 | 6.72 | 7.84 | 3.62 | 3.21 |
| Amaturá | 3.56 | -2.49 | 0.17 | 4.20 | 3.66 | 3.10 |
| Anamã | 0.87 | -0.78 | 9.21 | 0.69 | 4.27 | 3.72 |
| Anori | 11.05 | -2.66 | 4.06 | 1.75 | 4.07 | 3.54 |
| Apuí | -7.93 | -15.15 | -7.20 | -5.89 | 4.64 | 3.51 |
| Atalaia do Norte | 27.09 | -5.30 | 1.90 | 6.11 | 4.21 | 3.29 |
| Autazes | -2.03 | -7.55 | 6.53 | 7.88 | 4.64 | 3.80 |
| Barcelos | -12.28 | -2.25 | 12.38 | 8.14 | 4.12 | 3.69 |
| Barreirinha | 18.54 | -22.74 | -10.59 | -10.30 | 5.08 | 3.80 |
| Benjamin Constant | 28.60 | -5.82 | -1.17 | 4.82 | 4.13 | 3.28 |
| Beruri | 15.34 | -4.42 | 1.90 | 1.49 | 4.08 | 3.47 |
| Boa Vista do Ramos | 15.00 | -20.65 | -8.34 | -8.50 | 4.99 | 3.81 |
| Boca do Acre | 29.10 | -9.02 | -2.81 | 3.99 | 5.04 | 3.67 |
| Borba | 0.14 | -4.68 | 5.28 | 5.16 | 4.32 | 3.63 |
| Caapiranga | -7.45 | -1.18 | 9.99 | 9.22 | 4.35 | 3.73 |
| Canutama | 10.60 | -14.30 | -5.61 | -5.20 | 4.55 | 3.51 |
| Carauari | 21.40 | -7.17 | -2.71 | 1.27 | 4.00 | 3.26 |
| Careiro | 1.47 | -11.82 | 3.53 | 8.85 | 4.85 | 3.85 |
| Careiro da Várzea | -4.01 | -5.02 | 7.95 | 8.42 | 4.53 | 3.78 |
| Coari | 7.43 | -1.62 | 5.05 | 7.37 | 3.80 | 3.31 |
| Codajás | -7.67 | 3.57 | 12.11 | 9.26 | 3.96 | 3.57 |
| Eirunepé | 25.91 | -7.38 | 0.75 | 5.21 | 4.54 | 3.40 |
| Envira | 17.25 | -7.74 | 1.53 | 5.35 | 4.78 | 3.48 |
| Fonte Boa | -6.97 | 5.90 | 7.60 | 6.25 | 3.52 | 3.20 |
| Guajará | 22.17 | -7.00 | 4.03 | 11.93 | 4.72 | 3.48 |
| Humaitá | -2.32 | -13.50 | -2.84 | -4.13 | 4.54 | 3.47 |
| Ipixuna | 20.32 | -7.43 | 4.07 | 11.60 | 4.61 | 3.43 |
| Iranduba | 2.61 | -9.01 | 4.55 | 10.68 | 4.78 | 3.81 |
| Itacoatiara | 14.72 | -16.04 | -2.61 | 1.47 | 4.87 | 3.81 |
| Itamarati | 29.01 | -10.42 | -3.22 | 0.04 | 4.49 | 3.44 |
| Itapiranga | 23.56 | -20.62 | -9.16 | -4.15 | 4.73 | 3.72 |
| Japurá | -5.65 | 1.76 | 4.61 | 3.94 | 3.48 | 3.08 |
| Juruá | -3.18 | 2.29 | 4.44 | 2.46 | 3.59 | 3.17 |
| Jutaí | 17.34 | -5.05 | -0.70 | 2.98 | 3.92 | 3.22 |
| Lábrea | 17.63 | -12.42 | -3.37 | -0.59 | 4.90 | 3.68 |
| Manacapuru | -5.13 | -5.43 | 6.54 | 9.67 | 4.57 | 3.79 |
| Manaquiri | 0.62 | -3.75 | 7.33 | 9.58 | 4.42 | 3.74 |
| Manaus | 13.61 | -15.19 | -1.51 | 5.79 | 4.94 | 3.78 |
| Manicoré | -2.53 | -10.08 | -1.88 | -2.03 | 4.34 | 3.39 |
| Maraã | -12.55 | 9.32 | 14.49 | 16.95 | 3.64 | 3.36 |
| Maués | 1.39 | -8.79 | 0.45 | 0.55 | 4.53 | 3.67 |
| Nhamundá | 36.58 | -22.88 | -11.24 | -6.29 | 4.71 | 3.66 |
| Nova Olinda do Norte | 4.00 | -8.76 | 4.62 | 12.05 | 4.67 | 3.77 |
| Novo Airão | 6.15 | -9.25 | 2.73 | 6.70 | 4.79 | 3.76 |
| Novo Aripuanã | -7.61 | -9.29 | 0.03 | -0.22 | 4.51 | 3.48 |
| Parintins | 21.56 | -25.26 | -13.60 | -13.69 | 5.06 | 3.80 |
| Pauini | 17.96 | -9.83 | -2.62 | -0.32 | 4.85 | 3.60 |
| Presidente Figueiredo | 31.19 | -21.09 | -9.34 | 0.58 | 5.04 | 3.77 |
| Rio Preto da Eva | 16.12 | -18.01 | -3.87 | 5.58 | 4.87 | 3.77 |
| Santa Isabel do Rio Negro | -8.60 | 4.94 | 17.73 | 14.65 | 3.62 | 3.34 |
| Santo Antônio do Içá | 7.61 | -2.01 | 0.91 | 1.80 | 3.67 | 3.11 |
| São Gabriel da Cachoeira | 2.47 | 1.59 | 6.70 | 6.55 | 3.48 | 3.13 |
| São Paulo de Olivença | 16.96 | -4.93 | -1.05 | 5.82 | 3.93 | 3.21 |
| São Sebastião do Uatumã | 32.08 | -21.94 | -10.88 | -1.56 | 4.73 | 3.72 |
| Silves | 21.08 | -19.38 | -7.52 | -3.24 | 4.80 | 3.75 |
| Tabatinga | 22.08 | -4.14 | -1.21 | 4.67 | 3.86 | 3.19 |
| Tapauá | 16.56 | -11.07 | -3.18 | -1.38 | 4.17 | 3.34 |
| Tefé | 7.04 | -1.32 | 2.70 | 6.24 | 3.74 | 3.21 |
| Tonantins | -3.22 | 0.25 | 1.65 | -1.08 | 3.50 | 3.09 |
| Uarini | -5.35 | 5.99 | 8.52 | 8.64 | 3.61 | 3.26 |
| Urucará | 36.35 | -21.14 | -11.05 | -1.06 | 4.73 | 3.69 |
| Urucurituba | 21.24 | -22.68 | -10.92 | -11.75 | 4.92 | 3.80 |

**Legend:** CDD (maximum number of consecutive days with daily precipitation amount lower than 1mm); PRCPTOT (annual total precipitation on wet days); R95p (annual total precipitation when daily precipitation amount on day was higher than 95 percentile); Rx5day (monthly maximum consecutive 5-day precipitation); Tmax (average annual maximum temperature) and Tmin (average annual minimum temperature).
