# Supplementary material for: Mapping human vulnerability to climate change in the Brazilian Amazon: The construction of a municipal vulnerability index
Source: PLoS One. 2018 Feb 14;13(2):e0190808. doi: 10.1371/journal.pone.0190808 (PMC5812563; doi:10.1371/journal.pone.0190808)
Supplement: S7 Table — (DOCX) [file pone.0190808.s013.docx]

**Table 7.** Percentiles used to assign scores to the quantitative variables.

| **MVI Component** | **Index** | **Indicator** | | **Percentiles** | **Score** | | | | |
| --- | --- | --- | --- | --- | --- | --- | --- | --- | --- |
|  |  |  |  |  | **0** | **1** | **2** | **3** | **4** |
| **Exposure** | **Vegetation Cover Index** | Native vegetation cover (%) | | P5, P50, P95 | ≥ 95.25 | 95.25 --\| 77.13 | 77.13 --\| 36.10 | 36.10 --\| 4.27 | ≤ 4,27 |
|  |  | Accumulated deforestation (%) | | P5; P25; P75; P95 | < 0.06 | 0.06 \|-- 0.2 | 0.2 \|-- 2.0 | 2 \|-- 6,16 | ≥ 6,16 |
|  | **Natural Disasters Susceptibility Index** | Consecutive dry days | | P5; P25; P75; P95 | < 11,50 | 11,50 \|-- 14,80 | 14,80 \|-- 21,30 | 21,30 \|-- 35,10 | ≥35,10 |
|  |  | Population at risk (%) | | P5; P25; P75; P95 | < 0,20 | 0,20 \|-- 0,95 | 0,95 \|-- 5,98 | 5,98 \|-- 25,76 | ≥ 25,76 |
|  | **Natural Disasters Occurrence Index** | Disaster occurrence (%) | | P5; P25; P75; P95 | < 1,43 | 1,43 \|-- 1,63 | 1,63 \|-- 2,24 | 2,24 \|-- 3,47 | ≥ 3,47 |
|  |  | Deaths related to natural disasters (%) | | P5; P25; P75; P95 | < 5,88 | 5,88 \|-- 11,76 | 11,76 \|-- 40,00 | 40,00 \|-- 125,00 | ≥125,00 |
| **Sensitivity** | **Diseases Associated with Climate Index** | Accidents with poisonous animals | Incidence | P2,5;P25;P75;P97,5 | <34,3 | 34,3\|-- 58,53 | 58,53\|-- 108,80 | 108,80\|-- 209,74 | ≥209,74 |
|  |  |  | Proportion | P2,5;P25;P75;P97,5 | <0,28 | 0,28\|-- 0,68 | 0,68\|-- 1,89 | 1,89\|-- 5,15 | ≥5,15 |
|  |  |  | Tendency | P2,5;P25;P75;P97,5 | <-31,53 | -31,53\|-- -11,82 | -11,82\|-- 16,75 | 16,75\|-- 59,70 | ≥59,70 |
|  |  | Malaria | Incidence | P2,5;P25;P75;P97,5 | <0,43 | 0,43 \|-- 16,99 | 16,99\|-- 101,87 | 101,87\|-- 222,04 | ≥222,04 |
|  |  |  | Proportion | P1;P25;P75;P99 | <0,01 | 0,01\|-- 0,36 | 0,36\|-- 2,37 | 2,37\|-- 20,86 | ≥20,86 |
|  |  |  | Tendency | P2,5;P25;P75;P97,5 | <-45,31 | -45,31\|-- -5,0 | -5,0\|-- 0,52 | 0,52\|-- 28,64 | ≥28,64 |
|  |  | Dengue | Incidence | P1;P25;P75;P99 | <2,12 | 2,12\|-- 13,40 | 13,40\|-- 160,13 | 160,13\|-- 472,40 | ≥472,40 |
|  |  |  | Proportion | P1;P25;P75;P99 | <0,01 | 0,01\|-- 0,02 | 0,02\|-- 0,50 | 0,50\|-- 84,34 | ≥84,34 |
|  |  |  | Tendency | P2,5;P25;P75;P97,5 | <-419,69 | -419,69\|-- -65,46 | -65,46\|-- 0,70 | 0,70\|-- 302,97 | ≥302,97 |
|  |  | American Cutaneous Leishmaniasis | Incidence | P2,5;P25;P75;P97,5 | <3,41 | 3,41\|-- 12,93 | 12,93\|-- 72,20 | 72,20\|-- 839,55 | ≥839,55 |
|  |  |  | Proportion | P2,5;P25;P75;P97,5 | <0,03 | 0,03\|-- 0,10 | 0,10\|-- 0,92 | 0,92\|-- 14,75 | ≥14,75 |
|  |  |  | Tendency | P2,5;P25;P75;P97,5 | <-94,22 | -94,22\|-- -10,85 | -10,85\|-- 1,35 | 1,35\|-- 18,97 | ≥18,97 |
|  | **Poverty Index** | Probability of dying before the age of 5 (1,000 live births) | | P5; P25; P75; P95 | <18,601 | 18,60\|-- 21,96 | 21,96\|-- 27,72 | 27,72\|-- 32,38 | ≥32,38 |
|  |  | Probability of dying before age 40 | | P5; P25; P75; P95 | <4,08 | 4,08\|-- 4,79 | 4,79\|-- 5,97 | 5,97\|-- 6,94 | ≥6,94 |
|  |  | Households with per capita income below the poverty line (%) | | P5; P25; P75; P95 | <51,23 | 51,23\|-- 63,93 | 63,93\|-- 72,92 | 72,92\|-- 80,61 | ≥80,61 |
|  |  | Households with inadequate sanitation (%) | | P1;P25;P75;P99 | ≤0,90 | 0,90 \|-- 32,20 | 32,20\|-- 47,80 | 47,80\|-- 80,00 | ≥80,00 |
|  |  | Population aged over 25 that is illiterate (%) | | P5; P25; P75; P95 | <10,36 | 10,36\|-- 15,03 | 15,03\|-- 33,33 | 33,33\|-- 43,57 | ≥43,57 |
|  | **Sociodemographic Sensitivity Index** | Young householders (%) | | P5; P25; P75; P95 | <3,23 | 3,23\|-- 4,07 | 4,07\|-- 5,07 | 5,07\|-- 5,88 | ≥5,88 |
|  |  | Female householders with incomplete primary school or no education (%) | | P5; P25; P75; P95 | <2,54 | 2,54\|-- 3,65 | 3,65\|-- 4,98 | 4,98\|-- 6,09 | ≥6,09 |
|  |  | Population with disabilities (%) | | P5; P25; P75; P95 | <12,34 | 12,34\|-- 16,99 | 16,99\|-- 22,31 | 22,31\|-- 25,73 | ≥25,73 |
|  |  | Population that is 60 years old or older (%) | | P5; P25; P75; P95 | <4,36 | 4,36\|-- 5,14 | 5,14\|-- 6,50 | 6,50\|-- 7,32 | ≥7,32 |
|  |  | Population under 5 years old (%) | | P5; P25; P75; P95 | <12,24 | 12,24\|-- 13,89 | 13,89\|-- 16,75 | 16,75\|-- 18,18 | ≥18,18 |
|  |  | Riverine population (%) | | P5; P25; P75; P95 | <4,89 | 4,89\|-- 24,44 | 24,44\|-- 45,72 | 45,72\|-- 93,76 | ≥93,76 |
|  |  | Elderly aged 60 years old or older for 2040 (%) | | P5; P25; P75; P95 | <0,110 | 0,110\|-- 0,124 | 0,124\|-- 0,157 | 0,157\|-- 0,18 | ≥0,18 |
|  |  | Children aged 0 to 4 years old for 2040 (%) | | P5; P25; P75; P95 | <0,052 | 0,052\|-- 0,568 | 0,568\|-- 0,63 | 0,63\|-- 0,698 | ≥0,698 |
| **Adaptive Capacity** | **Socioeconomic Structures Index** | Reverse FIRJAN Index | | P5; P25; P75; P95 | <0,401 | 0,401\|-- 0,502 | 0,502\|-- 0.577 | 0.577\|-- 0.630 | ≥0.630 |
|  | **Institutions, Services, and Infrastructure for Adaptation Index** | Health care services | Primary care coverage (%) | P5; P25; P75; P95 | ≥91,81 | 91,81--\|81,78 | 81,78--\|54,28 | 54,28--\|42,74 | <42,74 |
|  |  |  | Hospital beds (per 1,000 inhabitants) | P5; P25; P75; P95 | ≥3,76 | 3,76 --\|2,08 | 2,.08--\|1,25 | 1,25--\|0,74 | <0,74 |
|  | **Sociopolitical Organization Index** | Municipal councils and consortia related to adaptation to climate (nº) | | P5, P50, P95 | ≥8 | 5\|--\|3 | 3\|-- 2 | 2\|-- 1 | <1 |
| **Climate** | **Temperature Index** | Minimum temperature | | P5; P25; P75; P95 | <3,112 | 3,112\|-- 3,289 | 3,289\|-- 3,754 | 3,754\|-- 3,805 | ≥3,805 |
|  |  | Maximum temperature | | P5; P25; P75; P95 | <3,515 | 3,515\|-- 3,929 | 3,929\|-- 4,775 | 4,775\|-- 5,038 | ≥5,038 |
|  | **Precipitation Index** | CDD (days) | | P5; P25; P75; P95 | <-7,928 | -7,928\|-- -2,318 | -2,318\|-- 21,081 | 21,081\|-- 31,191 | ≥31,191 |
|  |  | PRECPTOT (mm) | | P5; P25; P75; P95 | <1,316 | 1,316\|-- 4,138 | 4,138\|-- 13,503 | 13,503\|-- 22,678 | ≥22,678 |
|  |  | R95p (mm) | | P5; P25; P75; P95 | <-10,919 | -10,919\|-- -3,176 | -3,176\|-- 5,047 | 5,047\|-- 12,108 | ≥12,108 |
|  |  | RX5day (mm) | | P5; P25; P75; P95 | <-8,504 | -8,504\|-- -0,593 | -0,593\|-- 7,836 | 7,836\|-- 11,927 | ≥11,927 |
