# Supplementary material for: Mapping human vulnerability to climate change in the Brazilian Amazon: The construction of a municipal vulnerability index
Source: PLoS One. 2018 Feb 14;13(2):e0190808. doi: 10.1371/journal.pone.0190808 (PMC5812563; doi:10.1371/journal.pone.0190808)
Supplement: S8 Table — (DOCX) [file pone.0190808.s014.docx]

**S8 Table**. Assignment of values to the qualitative variables used to perform the Institutions, Services, and Infrastructure for Adaptation Index (AdapI).

| **Existence of security institutions - Fire Department, Municipal Civil Defense or Municipal Guard units** | | **Index Value** |
| --- | --- | --- |
| The municipality has all of the surveyed institutions | | 0 |
| The municipality has 2 of the surveyed institutions | | 0,33 |
| The municipality has 1 of the surveyed institutions | | 0,66 |
| The municipality does not have any of the surveyed institutions | | 1 |
| **Existence of risk management instruments for landslides and floods** | | |
| **Has the municipality been hit by disaster?** | **How many of risk management instruments does the municipality have?** | **Index Value** |
| **Landslides** | | |
| No information | 1 or more instruments | 0 |
| No | 1 or more instruments | 0 |
|  | None or not applicable | 0,5 |
| Yes | 3 or more instruments | 0 |
|  | 2 instruments | 0,25 |
|  | 1 instrument | 0,75 |
|  | None or not applicable | 1 |
| **Floodings** | | |
| No | 1 or more instruments | 0 |
|  | None or not applicable | 0,5 |
| Yes | 3 or more instruments | 0 |
|  | 2 instrumentss | 0,25 |
|  | 1 instrument | 0,75 |
|  | None or not applicable | 1 |
