# Supplementary material for: Mapping human vulnerability to climate change in the Brazilian Amazon: The construction of a municipal vulnerability index
Source: PLoS One. 2018 Feb 14;13(2):e0190808. doi: 10.1371/journal.pone.0190808 (PMC5812563; doi:10.1371/journal.pone.0190808)
Supplement: S1 Appendix — (DOCX) [file pone.0190808.s015.docx]

**S1 Appendix. A brief description of the microregions of the state of Amazonas**

1. **Alto Solimões:** Population of 224,094 according to the 2010 census [1]. It possesses nine municipalities – Amaturá, Tabatinga, Atalaia do Norte, Tonantins, Benjamim Constant, Fonte Boa, Jutaí, Santo Antônio do Içá and São Paulo de Olivença. The region possesses a triple-border with Brazil-Peru-Colombia and has the lowest rates of social development in the Amazonas [2]. It comprises predominantly rainforest (non-floodable upland forest), but also igapós (blackwater-flooded forests) along the course of the Solimões River [3]. Production in this microregion is destined for family consumption, so family farming, fishing and rearing small animals are significant and responsible for driving the local and regional economy [2]. Access is considered difficult and is primarily by river, so the municipalities are classified as isolated [2]. It possesses a large indigenous population, with emphasis on the Ticuna group [3];
2. **Boca do Acre:** Population of 48,798 according to the 2010 census [1]. It possesses two municipalities – Boca do Acre and Pauini. It is located close to the Purus microregion and, therefore, shares many characteristics with it, such as the coexistence of distinct Amazons: the “extractivism Amazon” and the “frontier agriculture and livestock Amazon” [4]. The region is strongly integrated with the Brazilian states of Acre and Rondônia and locomotion is primarily fluvial [4]. In the northern portion of the microregion, extractivism and riverine populations prevail, with practically all of the forest still intact, while in the southern portion the process of opening and occupation of the forest by the advancement of the agriculture and livestock frontier is taking place [4];
3. **Coari:** Population of 152,163 according to the 2010 census [1]. It possesses six municipalities – Anamã, Anori, Beruri, Caapiranga, Coari and Codajás. The economy is based on extractivism, agriculture, fishing and, since the 1990s, the exploitation of natural gas and oil in the municipality of Coari. The development of these latter two activities have promoted rapid economic growth for the region (from royalties), resulting in an improvement in the social, cultural and economic levels of the region. The municipality of Coari is considered one of the richest municipalities in North Brazil, and houses the third largest sedimentary basin in oil production and the second largest reserve of natural gas in the country;
4. **Itacoatiara:** Population of 152,027 according to the 2010 census [1]. It possesses five municipalities – Itacoatiara, Itapiranga, Nova Olinda do Norte, Silves and Urucurituba. Itacoatiara is the regional hub of the microregion and serves as a sub-regional center for the other municipalities [5]. Access is by river (Madeira/Amazonas waterway), by air and by state and federal highways, and the port serves a prominent function of connecting urban centers [5]. Although lacking technology and diversification, agriculture occupies a prominent place in the economy, with activities developed in upland areas, which are occupied by perennial crops, and in seasonal floodplains (“várzeas”), where crops are seasonal [5];
5. **Japurá:** Population of 24,854 according to the 2010 census [1]. It possesses two municipalities – Japurá and Maraã. It comprises extensive river valleys and vegetation that differs from traditional ombrophilous forest – the campinarana [3]. Urban centers are modest and articulate via waterways. There is strong extractive activity, high mineral potential and numerous indigenous populations. Access to other regions is via river and air transport [3];
6. **Juruá:** Population of 127,845 according to the 2010 census [1]. It possesses seven municipalities – Carauari, Eirunepé, Envira, Guajará, Ipixuna, Itamarati and Juruá. The municipality of Juruá possesses field production of natural gas and petrochemicals, although the basis of the economy of this microregion is vegetal extractivism [2]. It is predominantly upland forest covered, but there also exists forests of “várzea” along the entire Juruá River valley. The main access routes are via river and air. Some municipalities have greater connection with the state of Acre (bordering to the south) and its capital, the city of Rio Branco;
7. **Madeira:** Population of 165,663 according to the 2010 census [1]. It possesses five municipalities – Apuí, Borba, Humaitá, Manicoré and Novo Aripuanã. Access to the municipalities is mainly via river and road (important federal roads, such as the Transamazonian Highway, are cut into the region), which are also the main means of communication with the rest of the state and the country [6]. Agriculture and livestock are focused mainly on family production and extractivism, although there is a modest livestock industry, mainly in the southern portion of the microregion [7]. In the same region, illegal logging presents a severe environmental problem;
8. **Manaus:** Population of 2,039,536 according to the 2010 census [1]. It possesses seven municipalities – Autazes, Careiro, Careiro da Várzea, Iranduba, Manacapuru, Manaquiri and Manaus (capital). It is an important industrial and commercial center due to the presence of the Manaus Free Zone, concentrating a large part of the state services, the Gross Domestic Product and the population of the state. In addition to industry, the other municipalities of the microregion stand out for the collection of rubber and Brazil nut, hunting, fishing, extensive livestock farming in natural fields and the cultivation of jute and black pepper. The microregion is served by air, road and river transportation, which interconnects the state both nationally and internationally. However, road transportation is the main means used for access to other regions of the state, but depends on the crossing of large rivers, such as the Negro and Solimões/Amazonas Rivers, by boats or ferries;
9. **Parintins:** Population of 242,860 according to the 2010 census [1]. It possesses seven municipalities – Barreirinha, Boa Vista do Ramos, Maués, Nhamundá, Parintins, São Sebastião do Uatumã, and Urucará. The vegetation cover is varied, from fields and “várzea” to upland rainforest. It is the second largest agricultural area of the Amazon, with the cultivation of cassava being the most important, but with other important crops such as guaraná, in the municipality of Maués, as well as jute, malva (mallow), cacao and corn [8]. Parintins is the hub municipality, with the best infrastructure and location – linking the Brazilian states of Amazonas and Pará – and has the responsibility to meet the needs of other municipalities, together with the municipality of Maués, in both the services sector and in state assistance [8];
10. **Purus:** Population of 69,516 according to the 2010 census [1]. It possesses three municipalities – Canutama, Lábrea and Tapauá. The main means of communication is fluvial, although the region is crossed by federal highways. It is an important center of cattle activity (beef and milk), has a high concentration of land ownership and has a high rate of deforestation. Access to the municipalities of this microregion is essentially by river and air. In this microregion, the “extractivism Amazon” (the northern portion, which is more dependent of the fluvial rhythm) and the “frontier of agriculture and livestock Amazon” (the southern portion, where deforestation and roads are more integrated to the rest of the country) coexist [4];
11. **Rio Negro:** Population of 96,483 according to the 2010 census [1]. It possesses four municipalities – Barcelos, Novo Airão, Santa Isabel do Rio Negro and São Gabriel da Cachoeira. Vegetal extractivism is the basis of the economy of this microregion, mainly the exploitation of piaçava (palm), borracha (rubber) and Brazil nut. It possesses a concentration of one of the largest indigenous populations of the Amazon. São Gabriel da Cachoeira is the largest urban center and articulator of agricultural trade. Due to its proximity to the Japurá microregion, it shares many biophysical characteristics with it, including extensive river valleys and a differentiated vegetation – the campinarana [3];
12. **Rio Preto da Eva:** Population of 52,894 according to the 2010 census [1]. It possesses two municipalities – Presidente Figueiredo and Rio Preto da Eva. In Presidente Figueiredo, the focus is mineral extraction of cassiterite, but there is also family agriculture and wood extractivism for civil construction [9]. Rio Preto da Eva is one of the largest citrus producers in the Amazon [9]. In recent years, the region has experienced development of some economic sectors that have raised the income of the population, manly rural tourism. The microregion is crossed by federal highway BR-174, which links the Brazilian states of Amazonas and Roraima to Venezuela;
13. **Tefé:** Population of 87,432 according to the 2010 census [1]. It possesses three municipalities – Alvarães, Tefé and Uarini. Upland forest predominates. The source of income for the population is based on family agriculture, with emphasis on the production of cassava, artisanal fishing and extractivism of Brazil nut, açaí (palm fruit), andiroba, copaiba (essential oil) and wood. Tefé is the most important municipality with the concentration of educational and financial services of the microregion, as well as serving as a tourist center in the central region of the Amazon due to the presence of the Reserva de Desenvolvimento Sustentável de Mamirauá (Mamirauá Sustainable Development Unit), a UNESCO natural heritage site.

**References**

1. Instituto Brasileiro de Geografia e Estatística - IBGE. Censo Demográfico 2010 [Internet]. 2010 [cited 2016 May 11]. Available from: https://sidra.ibge.gov.br/pesquisa/censo-demografico/demografico-2010/inicial.

2. Schor T. Redes, fluxos e abastecimento de comida no Alto Solimões/AM: reflexões sobre o papel das cidades e da produção rural no desenvolvimento local. Terceira Margem Amazonica. 2015;1(5):89–110.

3. Rodrigues EA. Rede urbana do Amazonas: Tefé como cidade média de responsabilidade territorial na calha do Médio Solimões. Universidade Federal do Amazonas; 2011.

4. Secretaria de Estado do Meio Ambiente e Desenvolvimento Sustentável do Amazonas - SDS, Cooperação Internacional para o Desenvolvimento – GIZ. Zoneamento econômico-ecológico da Sub-região do Purus: volume 02: situação atual e proposta para zoneamento ecológico econômico. Manaus; 2011.

5. Oliveira CNS. Urbanização no médio Amazonas: a importância de Itacoatiara/AM como cidade intermediária. Universidade Federal do Amazonas; 2007.

6. Silva RS da, Silveira RLL da. Meios de transporte e desenvolvimento regional no estado do Amazonas: uma análise das microrregiões geográficas do Madeira e do Purus. Novos Cad NAEA. 2013;15(2):45–84.

7. Cenamo MC, Carrero GC, Soares PG. Redução de Emissões do Desmatamento e Degradação Florestal (REDD+): Estudo de Oportunidades para a Região Sul do Amazonas. Série Relatórios Técnicos. Manaus; 2011.

8. Ramos AN. Política de desenvolvimento rural do MDA: a ausência da tradução no reconhecimento da identidade social no território rural do baixo Amazonas (AM). Universidade Federal do Amazonas; 2015.

9. Rodrigues EP, Pinheiro E da S. The deforestation along the BR-174 road (Manaus/AM-Boa Vista/RR). Soc Nat. 2011;23(3):513–28.
